# Serum lipidomic analysis from mixed neutron/X-ray radiation fields reveals a hyperlipidemic and pro-inflammatory phenotype

**DOI:** 10.1038/s41598-019-41083-7

**Published:** 2019-03-14

**Authors:** Evagelia C. Laiakis, Monica Pujol Canadell, Veljko Grilj, Andrew D. Harken, Guy Y. Garty, Giuseppe Astarita, David J. Brenner, Lubomir Smilenov, Albert J. Fornace

**Affiliations:** 1 Department of Oncology, Lombardi Comprehensive Cancer Center, Georgetown University, Washington, DC USA; 2 Department of Biochemistry and Molecular & Cellular Biology, Georgetown University, Washington, DC USA; 3 Center for Radiological Research, Columbia University, New York, NY USA; 4 Radiological Research Accelerator Facility, Columbia University, Irvington, NY USA

## Abstract

Heightened threats for nuclear terrorism using improvised nuclear devices (IND) necessitate the development of biodosimetry assays that could rapidly assess thousands of individuals. However, the radiation exposures from an IND may be complex due to mixed fields of neutrons and photons (γ-rays), shielding from buildings, and proximity to the epicenter among others. In this study we utilized lipidomics to analyze serum samples from mice exposed to various percentages of neutrons and X-rays to a total dose of 3 Gy. Triacylglycerides, phosphatidylserines, lysophosphatidylethanolamines, lysophosphatidylcholines (LPCs), sphingolipids, and cholesteryl esters all showed delayed increases at day 7 compared to day 1 after irradiation, while diacylglycerides decreased in mixed field exposures and phosphatidylcholines (PCs) remained largely unchanged. Individual lipid molecules with a high degree of unsaturation exhibited the highest fold changes in mixed fields compared to photons alone. More importantly, the increased ratio of LPCs to PCs of each irradiation group compared to control could be used as a radiation biomarker and highlights the existence of a pro-inflammatory phenotype. The results showed that even a small percentage of neutrons in a mixed field can lead to high biological responses with implications for accurate biodosimetry, triage and medical managements of exposed populations.

## Introduction

Nuclear proliferation and the increased threat of nuclear terrorism with improvised nuclear devices (INDs) have accelerated the need for rapid and accurate radiation biodosimetry. Methods such as metabolomics^1,2^, lipidomics^2,3^, proteomics^4,5^, and transcriptomics or gene expression^6,7^ have been employed to develop panels of biomarkers in easily accessible biofluids to rapidly test thousands of individuals in case of an emergency in order to provide triage and appropriate medical treatment in a timely manner.

Radiation exposures encountered in an IND situation will be far more complex than single fields, i.e. γ-rays, as may be encountered in an accidental exposure or by a radiological dispersal device (RDD). It is estimated that even at 1.5 km from an IND an individual will be exposed to a mixed field of photons and neutrons. As neutrons have a high relative biological effectiveness (RBE), a low dose can lead to a significant biological effect. It has been calculated that the RBE in a mixed field may only be primarily dependent on the neutron dose and may be higher with decreasing neutron dose^8^. Therefore, a large population that is shielded in buildings or is situated at a distance from the epicenter where the neutrons may be less can still have a significant physiological response. It is therefore important to distinguish with the use of biological parameters the percentage of neutrons in an exposure to not only accurately estimate the absorbed dose but to further allow medical personnel to make informed decisions on a treatment course and to evaluate long term effects of radiation exposure. This is particularly important as evidence is emerging that radiation countermeasures are not universal for all types of exposures and their efficacy will depend on radiation quality^9^. In addition, the contribution of the more damaging radiation quality may lead to effects that can persist over years and can develop into systemic and organ specific issues, e.g. cardiovascular disease (CVD) or cancer^10,11^. The doses received by the atomic bomb survivors have previously been presented by Katayama *et al*. based on the ABD200D organ dose dosimetry system, reaching ≥4 Sv^12^, while Cullings *et al*. described in detail the evolution of the dosimetry system and the differences between Hiroshima and Nagasaki, including the neutron contribution^13^. Furthermore, astronauts in space and those that will take part in long term deep space missions encounter exposures to radiation particles such as neutrons, albeit different than the ones to be encountered in an IND, which can also be produced from secondary interactions of heavy ions with shielding material. As such, the neutron contribution may be significantly high and lead to biological effects such as central nervous system defects and development of CVD, to name a few. Therefore, understanding the effects of such exposures can help evaluate the risk of space radiation to missions.

We previously reported on comprehensive metabolomic and lipidomic analyses of rodent biofluids to distinguish between different radiation qualities, pure X-rays and neutron exposures^14^. Both metabolomics and lipidomics can provide a snapshot of the physiology at a given time point by assessing and quantifying, with relative or absolute parameters, nearly all metabolites and lipids under 1 kDa. Our previous results supported the higher damaging nature of neutrons compared to X-rays and the dyslipidemia resulting from such exposures^14^.

However, as mentioned earlier, real-life radiation exposure scenarios will be characterized by complexity, due to shielding and distance for example, and limitations on physical dosimetry will render accurate and rapid dosimetry difficult^15^. As such, it is estimated that neutrons may contribute 3% to 27% of the physical dose from an IND. Limited studies have analyzed such complicated scenarios to determine the effect of various neutron percentages in biological responses such as chromosomal aberrations^16^. A recent study on gene expression analysis of mouse blood determined that even small neutron contributions can have a profound effect^6^. Given the complexity of neutrons due to varying energies or travel patterns for example, biological responses may differ substantially depending on the energy used or percent contribution to a total dose. Additionally, the types of damage produced by the different types of irradiation are different in nature. Damage by neutrons leads to more clustered or complex lesions in DNA, as compared to electromagnetic radiation such as γ-rays and X-rays. Either of those types can be severely damaging and a mixed exposure may lead to higher levels of damage. We have therefore aimed to determine changes in the circulating lipidome following exposure to various neutron contributions to a total dose of 3 Gy and a comparable neutron total dose of 0.75 Gy with a 0.15 Gy γ-ray contribution, based on an assumed RBE of ~4^14,17^. This RBE is based on initial micronuclei studies by Xu *et al*.^17^, and has been implemented across our studies for our initial studies of neutron exposures on the metabolome and lipidome, although RBE calculations and especially for mixed fields can be far more complex. As previously presented, we utilized an accelerator neutron irradiation facility (Columbia University, Radiological Research Accelerator Facility RARAF)^14,17,18^, simulating neutrons similar to the Hiroshima atomic bomb spectrum, with an approximate 17–20% γ-ray component and energies between 0.2 and 9 MeV^14,17,18^. Lipidomics, defined as the collective assessment of all lipids in a sample with the use of modern liquid chromatography (LC) mass spectrometry (MS) techniques, showed delayed circulating increases in species from various lipid classes, with specific classes and individual lipids allowing for the distinction of the percentage of neutrons in the total dose. This is the first study to analyze mixed neutron/X-ray fields with lipidomics and can serve as the basis for dissecting complex biological interactions resulting from such irradiations. Potential long-term monitoring of such responses could also be correlated to biological effects and their severity.

## Materials and Methods

### Chemicals

All solvents were of the highest purity Optima LC-MS (Fisher Scientific). Internal standards used for lipid class retention time determination and normalization were included in the single mixture SPLASH® Lipidomix® mass spec standard (Avanti Polar Lipids, Inc).

### Experimental design and sample collection

Male C57BL/6J mice, 8–10 week old, were purchased from Charles River Laboratories and acclimated at Columbia University for one week prior to irradiation. All groups consisted of 5 mice per group. Mice were housed under standard housing conditions, with food and water *ad libitum* and 12:12 h light:dark cycle conditions. All experiments were approved by the Columbia University IACUC (#AC-AAAQ2410). All methods were performed in accordance with the relevant guidelines and regulations. Blood was collected at euthanasia at day 1 and 7 after irradiation via terminal cardiac puncture and serum was collected with serum separators (BD Microtainer® tubes, Becton Dickinson and Co, Franklin Lakes, NJ). Blood was allowed to clot for 30 minutes at room temperature, centrifuged at 4 °C for 5 min at 12,000 × *g*, transferred to Eppendorf tubes and immediately frozen and stored at −80 °C until shipment to Georgetown University.

### Irradiation and dosimetry

Irradiation and dosimetry have been previously described^14,18^ with use of custom holders in a Ferris wheel system to average the spatial inhomogeneity in the neutron beam. Mice were acclimated two times in their holders prior to irradiations for 15 min each. For mixed irradiations, mice were first irradiated with neutrons with an inherent γ-ray contribution of ~17%, followed immediately by X-irradiation for a total dose of 3 Gy. All mice that did not receive neutron irradiations were also subjected to the same treatment conditions, minus neutron exposure. Dose rates were as follows: neutrons 0.96 Gy/h, γ-rays 0.17 Gy/h, X-rays 1.23 Gy/min. The irradiation scheme per group is described in Table 1. No apparent radiotoxicity was observed in any of the mice over this 7-day study.

**Table 1:** Irradiation scheme in Gy.

Group designation	Neutron dose	Gamma ray dose	X-ray dose	Total dose	Neutron Fraction
Sham	0	0	0	0	0
0%	0	0	3	3	0%
83%	0.75	0.15	0	0.9	83%
**Mixed Field**					
5%	0.15	0.03	2.82	3	5%
15%	0.45	0.09	2.46	3	15%
25%	0.75	0.15	2.1	3	25%

Note: Doses are physical doses. 3 Gy X-rays and 0.75 Gy neutrons are considered equitoxic.

### Sample preparation and analysis

Twenty five μl of serum were diluted with cold chloroform:methanol (2:1) in a 1:4 v/v ratio in a siliconized tube. Samples were vortexed and allowed to stand at room temperature for 5 min. Samples were vortexed again and centrifuged for 5 min at 13,000 × *g* at room temperature. The bottom organic phase was carefully transferred to a separate siliconized tube and vacuumed dried with no heat. The lipids were dissolved in 150 μl of 50:25:25% isopropanol:acetonitrile:water and 10 μl of SPLASH® Lipidomix® mass spec standard mixture (Avanti Polar Lipids, Inc., Alabaster, Alabama) were added to each sample.

Two μl were injected into an Ultra Performance Liquid Chromatography (UPLC) system by Waters Corporation for chromatographic separation. A CSH C18 column (130 Å, 1.7 μm, 2.1 × 100 mm) was used for chromatography at 60 °C. Mobile phase A included 50:50 water:acetonitrile + 0.1% formic acid +10 mM HCOONH_4_ and mobile phase B included 90:10 isopropanol:acetonitrile +0.1% formic acid +10 mM HCOONH_4_. The chromatographic gradient was set as follows: 0–8 min 60% A and 40% B, 8–9 min 100% B, 9–13 min 60% A and 40% B, with a flow rate of 0.45 mL/min. The UPLC was coupled to a Xevo G2® QTOF-MS (Waters, Milford MA), operated in both positive and negative ionization mode (ESI^+^ and ESI^−^) in MS^E^ function. Accurate mass was obtained by intermittent injections of leucine enkephalin used as Lockspray®. Quality controls from pooled samples were injected every 10 samples in order to monitor for retention time drift and chromatographic integrity. Chromatographic data were deconvoluted with the software Progenesis QI (NonLinear Dynamics, Newcastle UK) and peak alignment was conducted based on the best quality control sample chosen by the software. Putative identities for lipid class assignment were assigned through the database LIPID MAPS^19^. Only ions that had a putative identity were further scrutinized. The list of potential metabolites was further reduced by including only ions whose retention time was close to their class’ internal standard. Furthermore, only ions that had theoretical fragmentation matching to a lipid identity through Progenesis QI or an identified characteristic neutral loss, i.e. 184 *m/z* for PCs and LPCs.

Upon reduction of the list of putative metabolites, LPCs were normalized to 18:1(d7) LPC [M + H]^+^ = 529.3992 at 1.31 min, sphingomyelins (SMs) were normalized to 18:1(d9) SM [M + H]^+^ = 738.6483 at 4.67 min, phosphatidylcholines (PCs) to 15:0–18:1(d7) PC [M + H]^+^ = 753.6483 at 4.96 min, triacylglycerides (TGs) to 15:0–18:1(d7)-15:0 TG [M + NH4]^+^ = 829.7988 at 7.66 min, lysophosphatidylethanolamines (LPEs) to 18:1(d7) LPE [M-H]^−^ = 485.4465 at 1.3 min, and phosphatidylserines (PSs) to 15:0–18:1(d7) PS [M + Na-2H]^−^ = 797.6041 at 4.9 min. The chromatographic separation of the internal standards is shown in Supplementary Fig. 1. All other lipid classes that did not have a specified internal standard were normalized with the function “normalize to all compounds” through the software Progenesis QI.

Normalized data from both ESI^+^ and ESI^−^ were combined to form a single spreadsheet. The data were analyzed with the statistical software MetaboAnalyst 4.0^20^. Features with >75% of missing values were removed and variables with missing data were excluded. Data was Pareto scaled and statistically significant ions across all groups [false discovery rate (FDR) p-value < 0.05] were identified with the non-parametric Kruskal Wallis test. Heatmaps with the Euclidean distance measure and the clustering algorithm Ward were constructed with group averages. Data were also analyzed with the statistical software package MetaboLyzer^21^. Briefly, complete-presence ions with at least 70% presence in both control and experimental groups, were analyzed with Welch’s t-test, while partial-presence ions (70% presence in only one group) were analyzed categorically with the Barnard’s test. Outliers were removed via 1.5 interquartile range (IQR) based. Values with zero abundance were excluded from the analysis. P-values were corrected with the Benjamini-Hochberg step-up FDR procedure (FDR < 0.1). Volcano plots were constructed from the complete presence ions, graphed as –log10 FDR corrected p-value versus log2 fold change.

Graphical representation was conducted with Prism 6 (GraphPad Software, Inc.). Outliers were identified with the ROUT method with Q set to 1%. P-values within each cluster of groups were calculated with the Kruskal Wallis test and multiple comparisons test through Dunn’s test, with a p < 0.05 considered statistically significant. Error bars are shown as standard error of the mean (SEM). Fold changes were calculated by dividing one experimental condition with another. Only fold changes of at least 1.5 (increased or decreased) were marked in supplementary tables.

## Results

Mixed neutron irradiation fields were achieved by first irradiating with varying doses of neutrons, as previously described^14,17,18^, followed shortly by X-ray irradiations to a total dose of 3 Gy. Groups were therefore designated as sham (controls), 0% (no neutrons) and an equitoxic dose of the neutron source (0.75 Gy) designated as 83%; the mixed field samples were designated 5% (0.15 Gy out of 3 Gy total), 15% (0.45 Gy out of 3 Gy total), and 25% (0.75 Gy out of 3 Gy total) (Table 1). For the neutron beam only (83%) relative toxicity has been previously assessed using a micronucleus approach^17^. Percent coefficient variance (%CV) and average values of the internal standards are presented in Supplementary Table 1. Initial analysis determined whether the neutron contribution can be detectable when compared to a pure X-ray dose. Volcano plots (Supplementary Fig. 2A) at day 1 after IR showed limited responses based on the neutron contribution, however at day 7 responses were augmented, with high levels of statistically significant ions at 15% neutrons and higher. Similar results were obtained when compared to sham irradiated (controls), as seen in Supplementary Fig. 2B; however, even a small percentage of neutrons (5%) was able to mount a more severe response indicating higher damage responses compared to pure photons.

**Figure 1:**
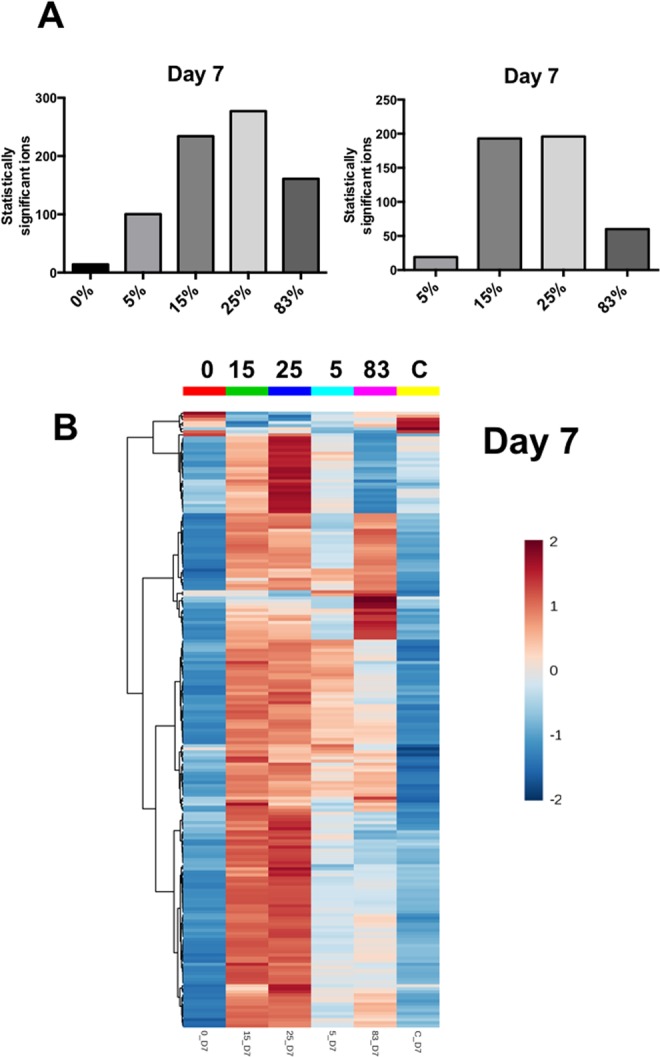
Panel (A) Number of statistically significant ions compared to control (left) and 0% neutrons at day 7 post irradiation. Panel (B) Heatmap depiction of the top 100 statistically significant ions as identified through the non-parametric Kruskal Wallis test.

Figure 1A shows the collective number of statistically significant features when comparing each exposure to controls (left panel) or to pure photons (right panel). A percentage dependent increase of lipidomic changes was evident compared to controls, although the 83% group showed a decrease in the total number of lipids, potentially signifying a higher degree of systemic damage that is associated with neutrons and has been cleared. The 5–25% groups therefore indicate the possibility of an additive response with the different radiation qualities compared to controls. Investigating the contribution of the neutrons to the overall signature compared to pure photons (Fig. 1A, right panel), 5% neutrons contributed minimally to lipidomic changes, while the 83% group showed a moderate response. The intermediate groups however (15 and 25%), highlighted the potential existence of a threshold for lipidomic responses and a synergistic effect of a mixed neutron/photon field to elicit a robust lipidomic response. A heatmap of day 7 data depicting the patterns of the top 100 lipids determined through a Kruskal Wallis test is shown in Fig. 1B, with the 25% group showing the highest changes compared to the other groups.

Statistically significant lipids were identified through the software MetaboAnalyst^20^ with a Kruskal Wallis test with FDR correction. All identities were based on searches through the database LIPID MAPS^19^, retention time proximity to the internal standard for each class, and existence of theoretical fragmentation pattern matching in Progenesis QI. Lipid classes that were identified in ESI^+^ include TGs, CEs, sphingolipids, DGs, LPCs, and PCs. Lipid classes that were perturbed in ESI^−^ include LPEs and PSs. In particular, 153 TGs were identified, 5 CEs, 6 sphingolipids, 7 DGs, 5 LPCs, 59 PCs, 4 LPEs, and 21 PSs (Supplementary Tables 2–13). Fold-change calculation was conducted by dividing mixed or pure neutrons (5–83%) to photons (0%) (Supplementary Tables 2, 3, 6, 7, 10, 11) or irradiated groups to controls (Supplementary Tables 4, 5, 8, 9, 12, 13). Fold changes were considered important if the ratio was ≥1.5 or ≤0.67 (representing a 1.5 fold decrease) for decreased levels and labeled with the symbol • in each Supplementary Table. The greatest fold changes were observed at day 7 [e.g. TG(63:10) reached >9 fold change compared to photons (Supplementary Table 3)]. The fold changes and increases were more pronounced in TGs with the higher carbon content and polyunsaturation levels, indicating the existence of processes such as cell damage and oxidative stress. Similar patterns were observed for PCs, while LPEs and PSs showed comparable changes at both days.

**Figure 2:**
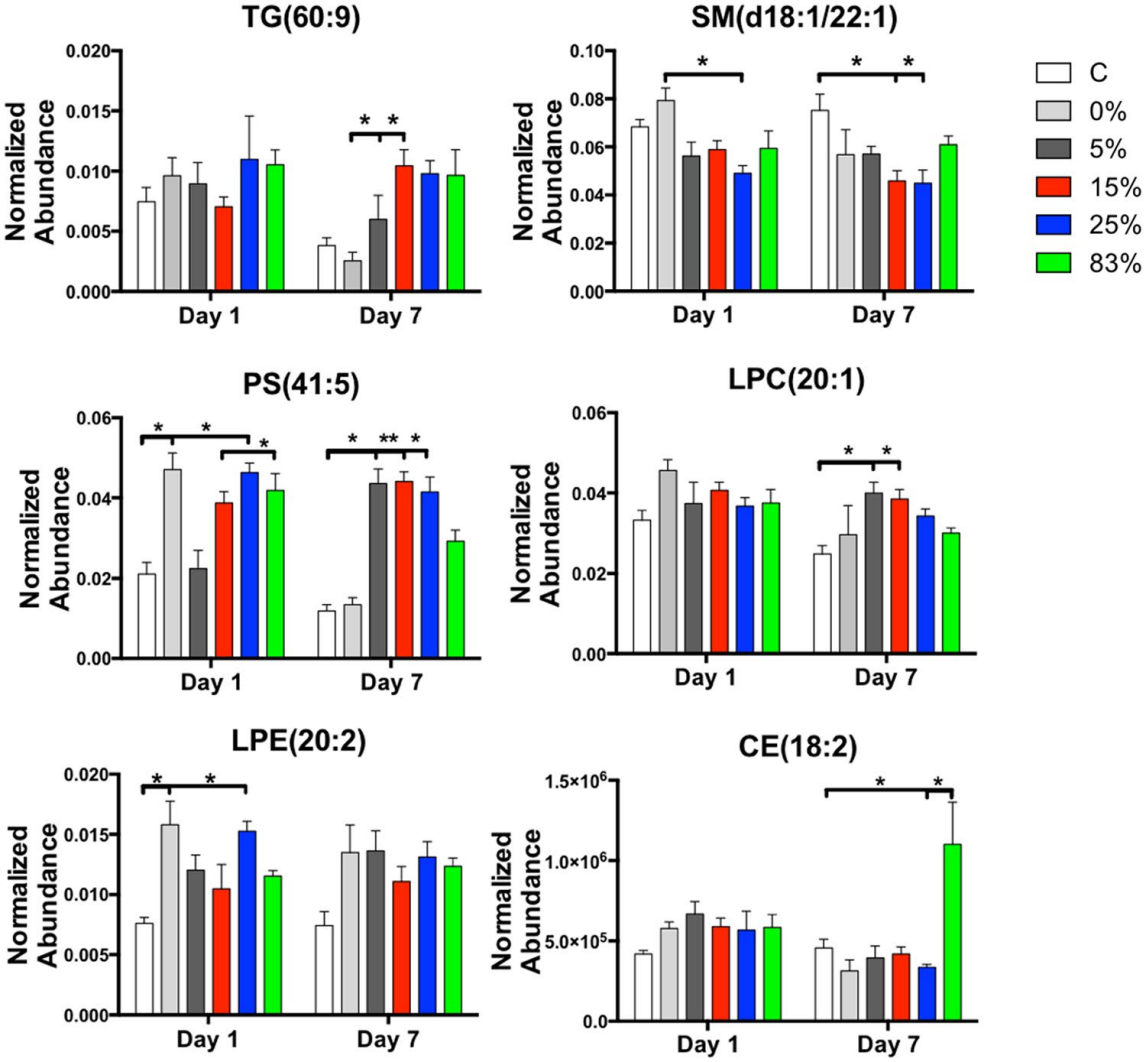
Representative lipids from each of the lipid classes that were identified in the analysis. Neutron specific changes or indiscriminate radiation changes can be observed, primarily at day 7 post irradiation. Data are presented as mean + standard error of the mean (n per group is 5).

Representative lipids from six different classes are shown in Fig. 2. TG(60:9), PS(41:5), and LPC(20:1) all showed mixed field increases when compared to controls or photons (0%) at day 7. LPE(20:2) showed early responses at day 1 that persisted in irradiated groups compared to control at day 7, while SM(d18:1/22:1) showed decreased changes in the mixed field groups. Finally, CE(18:2) showed a statistically significant increase in the 83% group at day 7, with no changes observed in the other groups.

**Figure 3:**
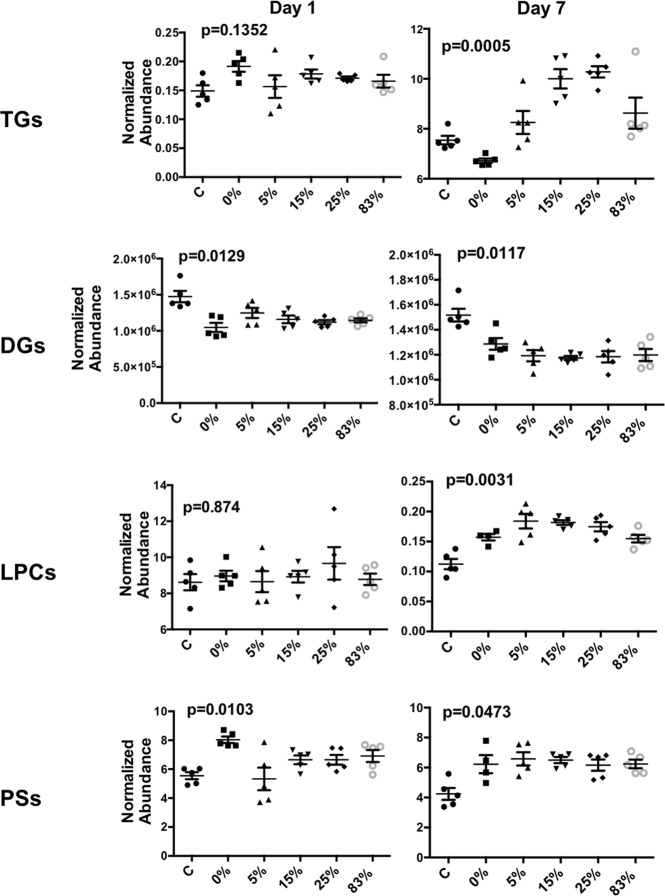
Total lipid abundance at day 1 and day 7 post irradiation. PSs, LPCs, and TGs all showed patterns of significant increases in the mixed neutron fields, which DGs mirrored the pattern of TGs and showed decreased overall levels with time. P-values were calculated with a Kruskal Wallis test with multiple comparison correction. Lines represent mean ± standard error of the mean (n per group is 5).

**Figure 4:**
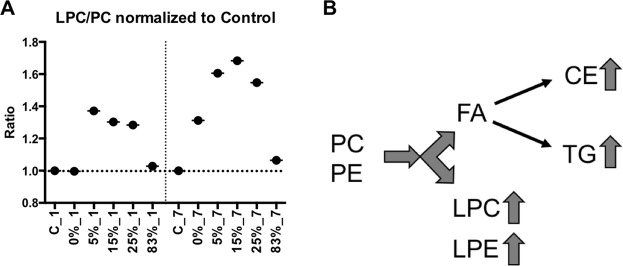
Panel (A) Ratio of total LPC to PC, normalized to mean control levels depicting the increased inflammatory nature of mixed field irradiations (n per group is 5). Panel (B) Lipid biosynthesis and altered patterns of neutron/X-ray exposures relative to control in the first week post irradiation. Lipid class abbreviations: CE, cholesteryl ester; DG, diacylglycerol; FA, fatty acid; LPC, lysophosphatidylcholine; LPE, lysophosphatidylethanolamine; PC, phosphatidylcholine; PE, phosphatidylethanolamine; TG, triacylglyceride.

To determine whether lipid classes exhibited differential levels based on the different experimental conditions, the normalized abundance levels of the identified lipids (Supplementary Tables 2, 3, 6, 7, 10, 11) were summed within each sample. This allowed for the collective assessment of each lipid class in each sample and group. Results for TGs, DGs, LPCs, and PSs are shown in Fig. 3. At day 1, only PSs and DGs showed statistically significant changes (p = 0.0129 for DGs and p = 0.0103 for PSs). However, at day 7 all four lipid classes showed significant changes with a p < 0.05, with TGs, LPCs, and PSs levels increasing with increasing neutron percentage. DGs on the other hand showed a pattern in the opposite direction, that mirrors the increases in TGs in each group. CEs, PEs, and sphingolipids total responses were less pronounced (data not shown). Since the ratio of LPCs to PCs is generally indicative of a pro-inflammatory response and phospholipase A activity, we calculated the ratio of LPC/PC between each irradiation condition compared to control (Fig. 4A). Results showed that mixed field irradiation had a dramatic contribution to this increased ratio, while pure photons only showed such a response at the later time point. The neutron beam only (83%) on the other had a very mild response compared to controls. Taken all together to identify patterns of changes in overall lipid classes, Fig. 4B shows a simplified version of the pathways of lipid biosynthesis in the first week after irradiation, as explored in this study and the interpathway connectivity.

## Discussion

This study focused on the effects of mixed neutron/photon irradiations on the mouse lipidome and whether such alterations can be used to estimate the percent of neutrons in the total absorbed dose for biodosimetry purposes. We previously reported on the radiation quality differences with equidose or equitoxic doses^14^, where we showed that neutrons exhibited a persistently higher degree of circulating lipids molecules compared to pure photons. Such collective changes were validated in this study (Fig. 1A, left panel). Lipid perturbations following whole body exposures have been well documented in multiple species and particularly in atomic bomb survivors^2,22–26^. However, specific lipids from different classes that could be used as biomarkers have been less characterized. Due to increased oxidative stress associated with ionizing radiation, lipid peroxidation and pro-inflammatory lipid intermediates can have immediate effects on physiology and even lead to longer term effects, such as development of CVD, lung injury, and even cancer^27,28^. Additionally, apoptosis from the direct action of radiation injury or even initiation of such processes from activation of lipid intermediates, as in activation of sphingomyelinase to generate ceramide from hydrolysis of sphingomyelin^29^, can lead to pathogenesis of normal tissue. Products of processes like these however can be mined as biomarkers to be used for assessment of exposures. Efforts such as this have been undertaken with gene expression studies with identical exposures as the ones we described here^6^. Pathways identified and related to metabolism included amino acids and fatty acid metabolism, as previously determined in metabolomic studies in urine and serum^14^, with significant downregulation of genes encoding for ribosomal proteins^6^. A future metabolomics specific analysis will address any perturbations in these pathways with a mixed field. These investigators also determined that the number of differentially expressed genes increased with the percentage of neutrons in the mixed field, except for the 25% group possibly reflecting increased damage^6^.

Our results showed that when compared to controls, there is a percentage mixed field dependent increase in statistically significant ions. Interestingly, the 83% group (0.75 Gy of neutrons) had a lower level of statistically significant ions compared to the 25% group (0.75 Gy of neutrons), although the collective number at the 25% vs. C suggests a synergistic rather than an additive interaction between the two types of radiation exposure. Possible explanations for this could include not only higher apoptotic products but also mechanisms resulting from increased oxidative stress that amplify the signal from a mixed field irradiation. When investigating the contribution of the neutron exposure to the total dose, as seen in Fig. 1A right panel, the 15 and 25% contributions appeared to have a similar effect when compared to photons alone (Fig. 1A). Compared to the 5% group, this suggests the existence of a possible threshold effect regarding lipidomic responses. Compared to pure photons (0%) and to “pure” neutrons (83%), the effect appears to be synergistic for both 15 and 25%, as when compared to controls.

The majority of the lipid responses showed the most pronounced changes at the later time point (day 7). Total TGs were severely increased at D7 compared to both control and pure photons (0%). Interestingly, TGs with high carbon and double bond content showed the highest fold changes at day 7. Such increases of species of TGs have previously been identified in non-human primates and attributed to increased oxidative stress^30^. Although the primary TG source is from dietary intake, the mirrored decrease in circulating DGs, coupled with no differences in body weight between the groups (Supplementary Fig. 3), suggests the possibility that such TG formation is of endogenous origin. Accumulation of TGs has been linked to increased macrophage apoptosis through mitochondrial dysfunction^31^ and increased risk of CVD, including atherosclerosis. Epidemiological studies of A-bomb survivors have shown increased risk of CVD and hypertension^10,32–34^. Increased levels of PSs also highlight the increased levels of apoptosis associated with radiation exposure, as PSs are used by cells as a clearance signal^35^, although the total levels are non-radiation quality specific. However, individual lipid molecules, such as PS(41:5) highlighted in Fig. 2 can be used as biomarkers associated with specific radiation qualities at later time points.

Along with increased TGs, high levels of circulating LPCs have also been linked to increased atherosclerosis^36,37^. LPCs are pro-inflammatory in nature and can be formed from oxidized low density lipoprotein (LDL)^36,37^. LPCs are also generated from hydrolysis of PCs through the enzymatic process of phospholipase A2. The ratio of LPC levels to PC levels has found utility as a good biomarker of increased inflammation^38^. In our study mixed field neutron exposures showed significantly higher levels compared to unirradiated controls, particularly at the later time point (Fig. 4A), highlighting the underlying inflammatory nature associated with mixed field irradiations, which is much higher than photons alone. As previously determined in our studies^14^, pure neutron irradiations are associated with a severe downregulation of metabolic processes and even pro-inflammatory lipid intermediates such as linoleic acid and arachidonic acid. A low ratio of LPC to PC has been associated with increased mortality in septic patients^39^, therefore it will be interesting to conduct long term studies and link the levels of these lipid classes to mortality associated with mixed field exposures.

Compared to our previously published study^14^, this study validated the patterns that were identified. Previous analysis of equidoses (1 Gy neutrons compared to 1 Gy X-rays) showed increased TGs, LPEs, and LPCs one week after irradiation, similar to what was observed in this more detailed analysis. Validation therefore of the results with an independent study highlights that neutron exposures can lead to higher responses that may potentially contribute to downstream effects. It remains to be seen what long-term effects will arise from mixed field irradiations and whether biomarkers for each % neutron contribution will persist overtime, as with the LPC/PC ratio. Nonetheless, early effects from mixed field irradiations can be identified through lipidomics and specific lipid species or classes utilized for determining the type of irradiation and potential health effects that can arise from such exposures. Furthermore, as this was an animal study with controlled experimental conditions, e.g. diet and genotype to name a few, it still remains to be seen whether similar results would be obtained in a real life scenario. Additionally, future research with lipidomics should also address the differences in populations such as underlying infections and increased stress, however our previous study on pure neutrons showed a significant downregulation of free fatty acids^14^, opposite of what is expected during an infection^40^. Nonetheless, this study provides valuable insight in the action of mixed beams on the lipidome.

## Supplementary information

Supplementary Figures and Tables
