# Supplementary material for: Serum lipidomic analysis from mixed neutron/X-ray radiation fields reveals a hyperlipidemic and pro-inflammatory phenotype
Source: Sci Rep. 2019 Mar 14;9:4539. doi: 10.1038/s41598-019-41083-7 (PMC6418166; doi:10.1038/s41598-019-41083-7)
Supplement: Supplementary file 1 — Supplementary Figures and Tables [file 41598_2019_41083_MOESM1_ESM.pdf]

Serum lipidomic analysis from mixed neutron/X-ray radiation fields reveals a hyperlipidemic and pro-inflammatory phenotype.

Evagelia C. Laiakis[1,2,*], Monica Pujol Canadell[3], Veljko Grilj[4], Andrew D. Harken[4], Guy Y. Garty[4], Giuseppe Astarita[2], David J. Brenner[3], Lubomir Smilenov[3], Albert J. Fornace Jr.[1,2]

1 Department of Oncology, Lombardi Comprehensive Cancer Center, Georgetown University, Washington DC USA.

2 Department of Biochemistry and Molecular & Cellular Biology, Georgetown University, Washington DC USA.

3 Center for Radiological Research, Columbia University, New York NY.

4 Radiological Research Accelerator Facility, Columbia University, Irvington NY.

Supplementary Figure 1: Chromatographic analysis of the internal standards included in the lipid mixture. Not all compounds ionized successfully with the method used in this study.

Supplementary Figure 2: Panel A: Volcano plots of contribution of varying mixed neutron fields compared to pure photons demonstrate the delayed response (day 7), particularly enhanced at the 15% and 25% groups. The red line signifies a 0.05 p-value cutoff. Panel B: Volcano plots of contribution of the different irradiation conditions to an altered lipidome at day 1 and day 7 after irradiation compared to control.

Supplementary Figure 3: Weights on mice immediately before placing in metabolic cages for a 24-hour urine collection. No statistically significant differences were calculated in either group analysis. C is for control (sham irradiated), the numbers refer to % of neutrons.

Supplementary Figure 1

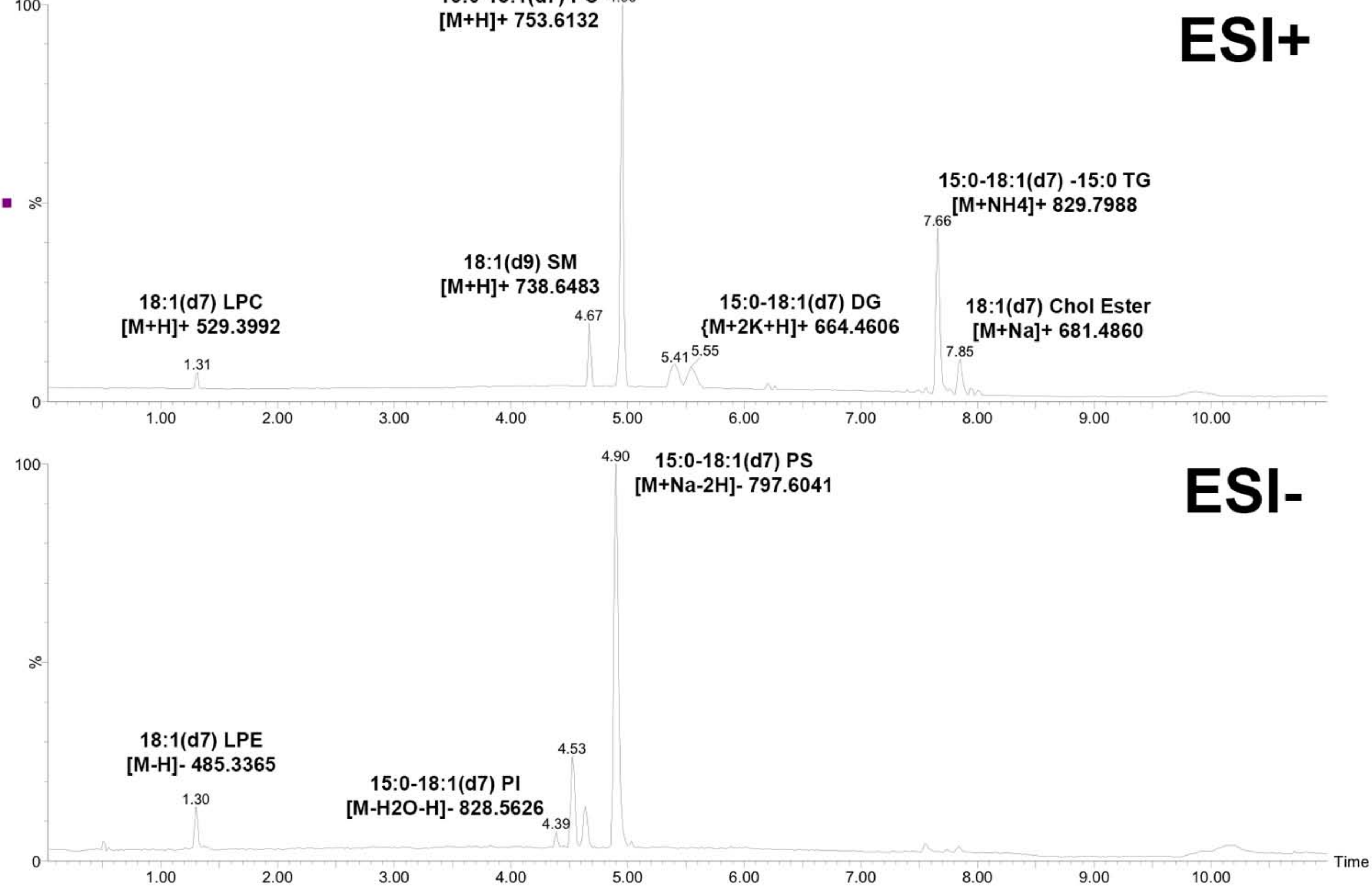

Supplementary Figure 2

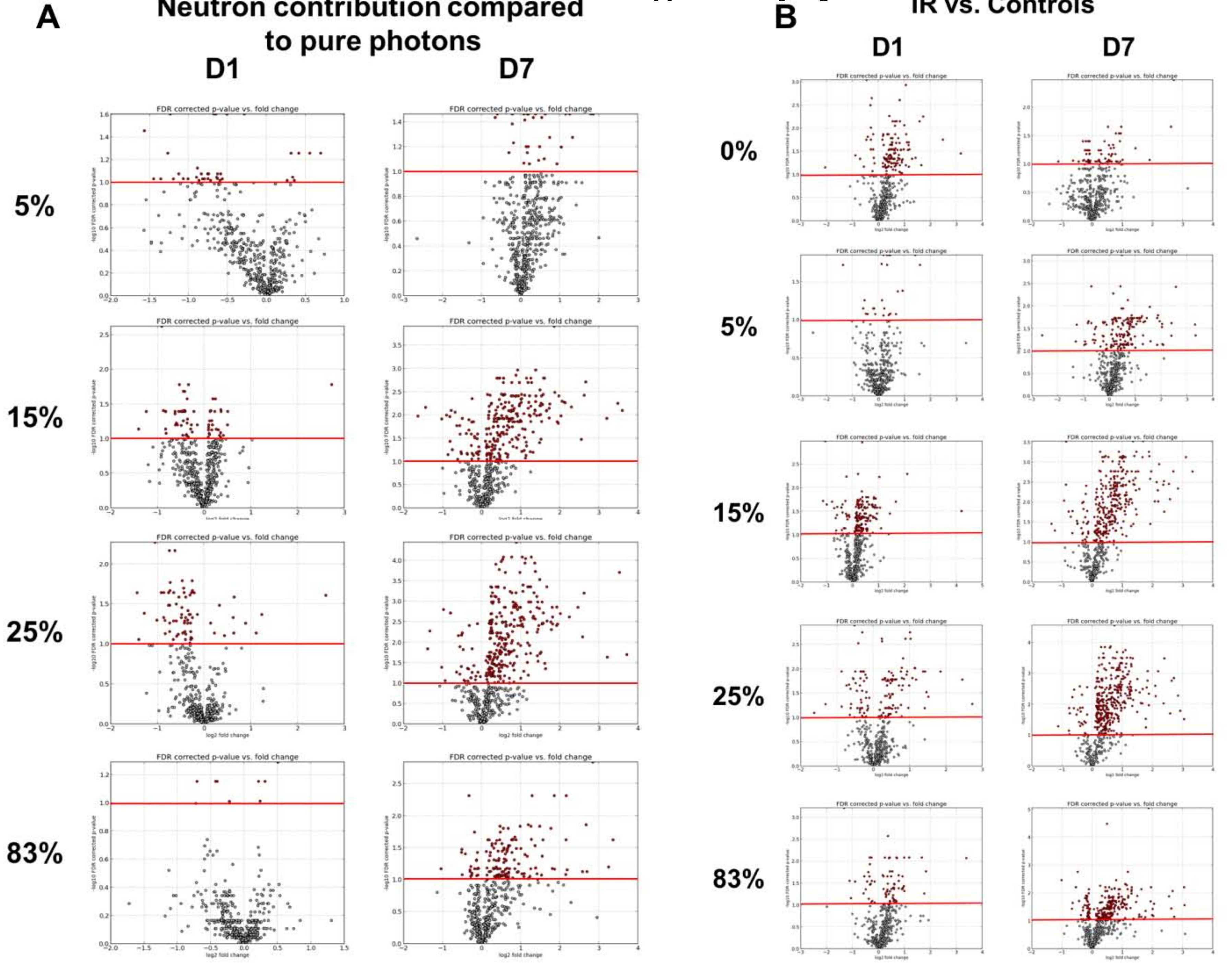

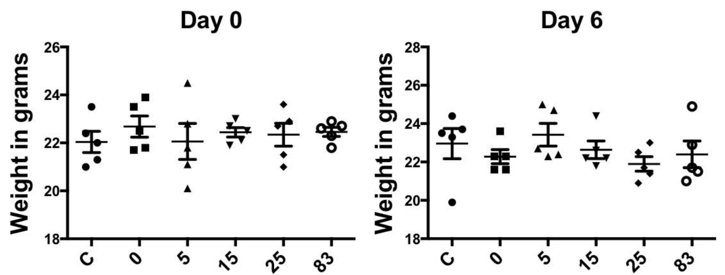

Supplementary Figure 3

Supplementary Table 1: Mean abundances and % coefficient variant of the internal standards used for normalization

	C_D1		0_D1		5_D1		15_D1		25_D1		83_D1	
	%CV	Mean	%CV	Mean	%CV	Mean	%CV	Mean	%CV	Mean	%CV	Mean
**LPC**	4.15	3.64E+06	6.45	4.38E+06	7.12	3.87E+06	8.77	4.15E+06	6.30	3.89E+06	5.10	4.06E+06
**SM**	7.26	1.04E+07	10.58	1.16E+07	3.95	1.35E+07	3.84	1.37E+07	4.91	1.36E+07	9.06	1.38E+07
**PC**	3.24	4.66E+07	14.42	5.44E+07	8.58	5.50E+07	6.37	5.41E+07	4.73	5.32E+07	11.70	5.34E+07
**TG**	5.03	2.73E+07	3.68	3.00E+07	5.29	3.12E+07	1.14	2.79E+07	12.99	3.01E+07	6.32	2.97E+07
**LPE**	3.43	2.22E+05	5.03	2.51E+05	5.27	2.26E+05	6.15	2.41E+05	3.82	2.42E+05	5.31	2.40E+05
**PS**	4.08	9.17E+06	3.04	1.05E+07	3.74	9.54E+06	5.69	9.69E+06	3.86	9.65E+06	3.58	9.91E+06
	**C_D7**		**0_D7**		**5_D7**		**15_D7**		**25_D7**		**83_D7**	
	**%CV**	**Mean**	**%CV**	**Mean**	**%CV**	**Mean**	**%CV**	**Mean**	**%CV**	**Mean**	**%CV**	**Mean**
**LPC**	4.30	3.60E+06	4.95	3.83E+06	6.58	3.75E+06	11.37	4.03E+06	5.24	4.33E+06	2.36	3.63E+06
**SM**	7.73	1.03E+07	19.00	1.08E+07	3.82	1.11E+07	7.53	1.17E+07	4.78	1.23E+07	1.40	1.09E+07
**PC**	5.61	4.72E+07	12.18	4.83E+07	6.23	5.02E+07	7.93	4.78E+07	12.97	5.11E+07	4.89	4.69E+07
**TG**	2.43	2.77E+07	3.33	3.15E+07	3.24	2.76E+07	6.13	2.76E+07	6.90	2.77E+07	5.54	3.10E+07
**LPE**	5.17	2.22E+05	3.60	2.33E+05	7.70	2.35E+05	3.30	2.27E+05	5.29	2.30E+05	3.19	2.38E+05
**PS**	4.91	9.32E+06	4.68	9.78E+06	2.19	9.48E+06	2.64	9.22E+06	3.53	9.17E+06	2.98	9.45E+06

Supplementary Table 2: Triacylglyceride levels Day 1 with fold changes of neutron contribution compared to photons.

m/z_Ret time	ID	C_D1		0_D1		5_D1		Fold change	15_D1		Fold change	25_D1		Fold change	83_D1		Fold change
		Average	SD	Average	SD	Average	SD	to X-ray (0%)	Average	SD	to X-ray (0%)	Average	SD	to X-ray (0%)	Average	SD	to X-ray (0%)
684.6134_6.79	TG(38:0)	0.03915	0.00227	0.04067	0.00174	0.04148	0.00178	1.02	0.04438	0.00249	1.09	0.03940	0.00463	0.97	0.04035	0.00483	0.99
712.6447_7.04	TG(40:0)	0.04016	0.00270	0.04050	0.00169	0.03802	0.00126	0.94	0.04154	0.00137	1.03	0.03749	0.00439	0.93	0.03940	0.00503	0.97
740.6753_7.27	TG(42:0)	0.04153	0.00274	0.04465	0.00283	0.04557	0.00134	1.02	0.04869	0.00092	1.09	0.04350	0.00519	0.97	0.04602	0.00506	1.03
738.6602_7.07	TG(42:1)	0.02113	0.00127	0.02152	0.00175	0.02175	0.00057	1.01	0.02340	0.00087	1.09	0.01998	0.00262	0.93	0.02245	0.00276	1.04
752.6753_7.18	TG(43:1)	0.01786	0.00084	0.01820	0.00109	0.01799	0.00042	0.99	0.01957	0.00040	1.08	0.01755	0.00229	0.96	0.01832	0.00197	1.01
768.7061_7.48	TG(44:0)	0.15072	0.00833	0.15449	0.00461	0.15088	0.00372	0.98	0.16118	0.00469	1.04	0.14610	0.01744	0.95	0.15180	0.00859	0.98
754.6905_7.36	TG(44:1)	0.02914	0.00185	0.02980	0.00091	0.02878	0.00034	0.97	0.03152	0.00053	1.06	0.02900	0.00410	0.97	0.02870	0.00340	0.96
766.6907_7.3	TG(44:1)	0.05195	0.00325	0.05497	0.00292	0.05399	0.00143	0.98	0.05813	0.00119	1.06	0.05262	0.00635	0.96	0.05463	0.00541	0.99
764.6761_7.12	TG(44:2)	0.01530	0.00078	0.01647	0.00126	0.01705	0.00045	1.04	0.01804	0.00075	1.10	0.01601	0.00178	0.97	0.01709	0.00184	1.04
762.6620_6.93	TG(44:3)	0.00184	0.00011	0.00192	0.00029	0.00200	0.00009	1.04	0.00207	0.00023	1.08	0.00190	0.00010	0.99	0.00199	0.00024	1.03
782.7216_7.56	TG(45:0)	0.13016	0.00717	0.13172	0.00471	0.12678	0.00374	0.96	0.13379	0.00434	1.02	0.12097	0.01402	0.92	0.13021	0.00560	0.99
780.7066_7.4	TG(45:1)	0.07367	0.00368	0.07426	0.00261	0.07166	0.00133	0.97	0.07812	0.00104	1.05	0.07076	0.00811	0.95	0.07397	0.00607	1.00
778.6909_7.23	TG(45:2)	0.01686	0.00075	0.01762	0.00114	0.01690	0.00018	0.96	0.01818	0.00068	1.03	0.01640	0.00219	0.93	0.01755	0.00191	1.00
796.7375_7.67	TG(46:0)	0.21243	0.01145	0.21594	0.01041	0.21183	0.00502	0.98	0.22412	0.00988	1.04	0.20468	0.02328	0.95	0.21439	0.00604	0.99
794.7222_7.51	TG(46:1)	0.26979	0.01521	0.27090	0.00854	0.26391	0.00715	0.97	0.28578	0.00776	1.05	0.26145	0.02925	0.97	0.26397	0.01506	0.97
792.7064_7.34	TG(46:2)	0.06729	0.00405	0.06784	0.00218	0.06610	0.00183	0.97	0.07186	0.00146	1.06	0.06631	0.00729	0.98	0.06724	0.00602	0.99
790.691_7.18	TG(46:3)	0.00913	0.00064	0.00963	0.00043	0.00953	0.00022	0.99	0.01040	0.00030	1.08	0.00931	0.00115	0.97	0.00955	0.00110	0.99
808.7380_7.59	TG(47:1)	0.29083	0.01605	0.29868	0.00991	0.29537	0.00898	0.99	0.31307	0.01273	1.05	0.28163	0.03120	0.94	0.30568	0.00954	1.02
818.7224_7.38	TG(47:1)	0.02350	0.00216	0.02465	0.00099	0.02323	0.00145	0.94	0.02500	0.00118	1.01	0.02395	0.00303	0.97	0.02376	0.00149	0.96
806.7227_7.43	TG(47:2)	0.06470	0.00367	0.06703	0.00218	0.06649	0.00235	0.99	0.07193	0.00227	1.07	0.06540	0.00720	0.98	0.06795	0.00459	1.01
804.7066_7.28	TG(47:3)	0.01071	0.00064	0.01157	0.00107	0.01177	0.00023	1.02	0.01241	0.00038	1.07	0.01111	0.00145	0.96	0.01192	0.00152	1.03
824.6723_7.85	TG(48:0)	0.00945	0.00054	0.00887	0.00079	0.00960	0.00070	1.08	0.00999	0.00058	1.13	0.00909	0.00102	1.02	0.00901	0.00074	1.02
822.7534_7.7	TG(48:1)	0.40609	0.02155	0.40607	0.01850	0.39300	0.01020	0.97	0.42209	0.01591	1.04	0.39237	0.03767	0.97	0.39612	0.01325	0.98
832.738_7.48	TG(48:1)	0.02542	0.00189	0.02664	0.00043	0.02633	0.00117	0.99	0.02845	0.00195	1.07	0.02638	0.00271	0.99	0.02693	0.00165	1.01
820.7382_7.55	TG(48:2)	0.17631	0.01140	0.17962	0.00852	0.17169	0.00723	0.96	0.17973	0.00834	1.00	0.17008	0.01519	0.95	0.17523	0.00528	0.98
816.706_7.16	TG(48:4)	0.00548	0.00043	0.00591	0.00076	0.00613	0.00053	1.04	0.00631	0.00010	1.07	0.00565	0.00089	0.96	0.00600	0.00106	1.01
838.7821_7.49	TG(49:0)	0.02601	0.00230	0.02640	0.00348	0.02881	0.00055	1.09	0.03171	0.00353	1.20	0.02743	0.00596	1.04	0.02815	0.00571	1.07
838.7821_7.92	TG(49:0)	0.15071	0.00587	0.15133	0.00878	0.15161	0.00506	1.00	0.16024	0.00518	1.06	0.14147	0.01750	0.93	0.15771	0.00703	1.04
813.6944_6.67	TG(49:4)	0.00241	0.00017	0.00250	0.00029	0.00257	0.00058	1.03	0.00309	0.00013	1.23	0.00261	0.00047	1.04	0.00254	0.00039	1.02
830.7187_7.3	TG(49:4)	0.00233	0.00035	0.00264	0.00035	0.00258	0.00021	0.98	0.00269	0.00050	1.02	0.00257	0.00051	0.97	0.00260	0.00045	0.98
822.6553_7.72	TG(49:8)	0.00165	0.00041	0.00210	0.00046	0.00193	0.00023	0.92	0.00222	0.00022	1.06	0.00178	0.00032	0.85	0.00207	0.00037	0.99
852.7989_8.02	TG(50:0)	0.13522	0.00544	0.14175	0.00953	0.13878	0.00302	0.98	0.14741	0.00629	1.04	0.13466	0.01119	0.95	0.14711	0.00690	1.04
850.785_7.87	TG(50:1)	0.48810	0.02434	0.48373	0.03808	0.46696	0.01519	0.97	0.50571	0.01450	1.05	0.49131	0.02189	1.02	0.45983	0.01758	0.95
848.7694_7.71	TG(50:2)	0.36508	0.03305	0.37116	0.02302	0.35068	0.03999	0.94	0.37194	0.02539	1.00	0.40896	0.08067	1.10	0.35839	0.01600	0.97
848.7698_7.22	TG(50:2)	0.00088	0.00025	0.00069	0.00015	0.00082	0.00007	1.18	0.00080	0.00010	1.16	0.00084	0.00037	1.23	0.00060	0.00022	0.87
846.7536_7.56	TG(50:3)	0.09831	0.01314	0.10163	0.01129	0.09644	0.01737	0.95	0.10118	0.01078	1.00	0.11234	0.02572	1.11	0.09916	0.00580	0.98
844.7376_7.37	TG(50:4)	0.01477	0.00306	0.01515	0.00301	0.01473	0.00524	0.97	0.01600	0.00270	1.06	0.01853	0.00545	1.22	0.01440	0.00238	0.95
825.6935_7.53	TG(50:5)	0.02862	0.00174	0.02796	0.00220	0.02795	0.00157	1.00	0.03082	0.00138	1.10	0.02737	0.00371	0.98	0.02702	0.00432	0.97
842.7155_7.20	TG(50:5)	0.01087	0.00165	0.01151	0.00126	0.01179	0.00269	1.02	0.01249	0.00101	1.08	0.01227	0.00197	1.07	0.01174	0.00192	1.02
823.6781_7.37	TG(50:6)	0.01024	0.00104	0.00975	0.00068	0.00962	0.00063	0.99	0.01088	0.00040	1.12	0.00971	0.00118	1.00	0.00945	0.00164	0.97
862.7845_7.79	TG(51:2)	0.23470	0.01061	0.24128	0.01422	0.24627	0.00820	1.02	0.26186	0.00741	1.09	0.23864	0.01802	0.99	0.25267	0.01011	1.05
860.7690_7.66	TG(51:3)	0.03810	0.00335	0.03955	0.00240	0.03851	0.00355	0.97	0.04122	0.00300	1.04	0.04139	0.00423	1.05	0.03943	0.00157	1.00
865.72_7.64	TG(51:3)	0.00973	0.00112	0.01014	0.00030	0.01032	0.00098	1.02	0.01123	0.00089	1.11	0.01073	0.00067	1.06	0.01024	0.00130	1.01
858.7526_7.48	TG(51:4)	0.00971	0.00139	0.01016	0.00107	0.01030	0.00221	1.01	0.01116	0.00121	1.10	0.01145	0.00264	1.13	0.01024	0.00114	1.01
863.7064_7.46	TG(51:4)	0.00338	0.00059	0.00346	0.00039	0.00360	0.00085	1.04	0.00400	0.00058	1.16	0.00375	0.00040	1.09	0.00361	0.00054	1.05
861.6860_7.31	TG(51:5)	0.00398	0.00084	0.00421	0.00082	0.00518	0.00057	1.23	0.00514	0.00065	1.22	0.00481	0.00076	1.14	0.00454	0.00068	1.08
880.8308_8.17	TG(52:0)	0.07877	0.00311	0.08697	0.01238	0.08966	0.00482	1.03	0.09075	0.00527	1.04	0.08027	0.00698	0.92	0.09308	0.00261	1.07
878.8152_8.03	TG(52:1)	0.16286	0.00794	0.16498	0.00574	0.16202	0.00469	0.98	0.17338	0.00800	1.05	0.17470	0.01827	1.06	0.16446	0.01027	1.00
876.8008_7.87	TG(52:2)	0.45744	0.03907	0.46770	0.05792	0.43447	0.04834	0.93	0.45975	0.02571	0.98	0.54875	0.19711	1.17	0.43167	0.01912	0.92
874.7852_7.71	TG(52:3)	0.41404	0.07702	0.44016	0.09740	0.37583	0.12709	0.85	0.37885	0.06116	0.86	0.58758	0.38154	1.33	0.38312	0.03304	0.87
872.7695_7.56	TG(52:4)	0.34355	0.08517	0.36568	0.08209	0.33754	0.15663	0.92	0.31631	0.07668	0.86	0.42195	0.20618	1.15	0.34402	0.05373	0.94
853.7274_6.77	TG(52:5)	0.00420	0.00073	0.00526	0.00070	0.00537	0.00107	1.02	0.00555	0.00078	1.06	0.00542	0.00176	1.03	0.00490	0.00101	0.93
870.7506_7.41	TG(52:5)	0.09186	0.02215	0.09091	0.01628	0.09196	0.03535	1.01	0.09484	0.01871	1.04	0.10809	0.03223	1.19	0.08994	0.01583	0.99
868.7359_7.23	TG(52:6)	0.02689	0.00502	0.02810	0.00414	0.02856	0.00874	1.02	0.03008	0.00381	1.07	0.03097	0.00577	1.10	0.02907	0.00494	1.03
849.6938_7.37	TG(52:7)	0.00733	0.00136	0.00729	0.00117	0.00703	0.00210	0.96	0.00813	0.00135	1.12	0.00880	0.00212	1.21	0.00683	0.00141	0.94
866.7223_7.13	TG(52:7)	0.00200	0.00041	0.00245	0.00036	0.00219	0.00065	0.90	0.00210	0.00029	0.86	0.00233	0.00061	0.95	0.00233	0.00058	0.95
866.7232_7.07	TG(52:7)	0.00259	0.00050	0.00286	0.00061	0.00307	0.00096	1.07	0.00342	0.00083	1.19	0.00328	0.00111	1.15	0.00300	0.00054	1.05
871.6738_7.13	TG(52:7)	0.00258	0.00060	0.00265	0.00060	0.00274	0.00083	1.03	0.00315	0.00043	1.19	0.00291	0.00058	1.10	0.00295	0.00064	1.11
894.7528_7.25	TG(52:7)	0.12764	0.02735	0.12868	0.02041	0.13327	0.04593	1.04	0.14182	0.02399	1.10	0.15711	0.03982	1.22	0.13469	0.02529	1.05
864.7126_6.92	TG(52:8)	0.00304	0.00062	0.00344	0.00066	0.00360	0.00082	1.05	0.00377	0.00076	1.10	0.00344	0.00082	1.00	0.00350	0.00097	1.02
890.8148_7.96	TG(53:2)	0.10277	0.00540	0.10323	0.00427	0.10727	0.00443	1.04	0.11157	0.00164	1.08	0.10158	0.01010	0.98	0.10884	0.00832	1.05
888.7989_7.37	TG(53:3)	0.00319	0.00036	0.00332	0.00036	0.00335	0.00044	1.01	0.00369	0.00023	1.11	0.00327	0.00083	0.98	0.00341	0.00065	1.03
888.7991_7.81	TG(53:3)	0.02882	0.00258	0.03066	0.00170	0.03120	0.00362	1.02	0.03292	0.00207	1.07	0.03346	0.00487	1.09	0.03164	0.00148	1.03
886.7846_7.66	TG(53:4)	0.01260	0.00224	0.01295	0.00188	0.01257	0.00289	0.97	0.01273	0.00164	0.98	0.01484	0.00464	1.15	0.01237	0.00094	0.96
882.7614_6.92	TG(53:6)	0.00319	0.00061	0.00375	0.00057	0.00405	0.00051	1.08	0.00381	0.00042	1.02	0.00393	0.00091	1.05	0.00381	0.00061	1.01
876.7090_7.88	TG(53:9)	0.00185	0.00039	0.00179	0.00037	0.00180	0.00036	1.01	0.00175	0.00038	0.98	0.00281	0.00213	1.57	0.00149	0.00026	0.84
906.8469_8.18	TG(54:1)	0.11034	0.00475	0.11495	0.01009	0.11907	0.00585	1.04	0.12490	0.00403	1.09	0.11343	0.00879	0.99	0.11818	0.00272	1.03
904.8307_8.03	TG(54:2)	0.13056	0.00774	0.13197	0.00690	0.13070	0.00644	0.99	0.13810	0.00750	1.05	0.14680	0.02798	1.11	0.13080	0.00831	0.99
902.8163_7.88	TG(54:3)	0.36768	0.02814	0.37088	0.03346	0.34766	0.02766	0.94	0.36850	0.02091	0.99	0.40924	0.09398	1.10	0.35033	0.01022	0.94
902.8238_7.46	TG(54:3)	0.01154	0.00153	0.01148	0.00120	0.01231	0.00202	1.07	0.01305	0.00138	1.14	0.01173	0.00370	1.02	0.01223	0.00246	1.07
900.8009_7.73	TG(54:4)	0.29218	0.05265	0.31557	0.06005	0.28152	0.09314	0.89	0.27926	0.04572	0.88	0.38019	0.19689	1.20	0.28824	0.02814	0.91
898.7852_7.56	TG(54:5)	0.38859	0.09652	0.43150	0.09097	0.42250	0.17829	0.98	0.40896	0.09880	0.95	0.52053	0.25171	1.21	0.42268	0.07300	0.98
879.7443_6.71	TG(54:6)	0.01113	0.00128	0.01399	0.00135	0.01418	0.00232	1.01	0.01459	0.00137	1.04	0.01379	0.00317	0.99	0.01383	0.00179	0.99
896.7684_7.41	TG(54:6)	0.39531	0.09199	0.40816	0.06389	0.41384	0.15473	1.01	0.41864	0.08730	1.03	0.46895	0.14211	1.15	0.41125	0.07713	1.01
877.7298_6.56	TG(54:7)	0.00384	0.00079	0.00405	0.00063	0.00408	0.00120	1.01	0.00425	0.00066	1.05	0.00397	0.00105	0.98	0.00388	0.00062	0.96
892.7406_7.08	TG(54:8)	0.02847	0.00618	0.02831	0.00448	0.02967	0.00909	1.05	0.03114	0.00457	1.10	0.03153	0.00692	1.11	0.02987	0.00497	1.06
920.8618_7.87	TG(55:1)	0.06260	0.00842	0.05990	0.00526	0.05743	0.00688	0.96	0.06895	0.00744	1.15	0.05782	0.01138	0.97	0.05398	0.01303	0.90
918.8466_8.12	TG(55:2)	0.02849	0.00150	0.02827	0.00184	0.02989	0.00180	1.06	0.03094	0.00080	1.09	0.02947	0.00174	1.04	0.02950	0.00277	1.04
908.7723_7.43	TG(55:5)	0.00369	0.00086	0.00437	0.00083	0.00419	0.00140	0.96	0.00396	0.00064	0.90	0.00478	0.00191	1.09	0.00471	0.00092	1.08
910.782_7.59	TG(55:6)	0.00615	0.00101	0.00696	0.00086	0.00671	0.00142	0.96	0.00668	0.00098	0.96	0.00749	0.00210	1.08	0.00751	0.00081	1.08
891.7352_7.64	TG(55:7)	0.00327	0.00052	0.00332	0.00034	0.00343	0.00062	1.03	0.00365	0.00037	1.10	0.00395	0.00057	1.19	0.00353	0.00038	1.06
889.7236_7.48	TG(55:8)	0.00182	0.00040	0.00170	0.00014	0.00181	0.00068	1.07	0.00207	0.00038	1.22	0.00200	0.00044	1.18	0.00167	0.00040	0.98
906.7594_7.33	TG(55:8)	0.00270	0.00062	0.00345	0.00059	0.00336	0.00105	0.97	0.00303	0.00017	0.88	0.00293	0.00088	0.85	0.00335	0.00082	0.97
887.7038_7.10	TG(55:9)	0.00093	0.00017	0.00144	0.00012	0.00155	0.00033	1.07	0.00158	0.00035	1.10	0.00135	0.00021	0.94	0.00175	0.00038	1.21
887.7120_7.33	TG(55:9)	0.00364	0.00054	0.00386	0.00055	0.00385	0.00048	1.00	0.00438	0.00050	1.14	0.00384	0.00060	0.99	0.00388	0.00071	1.01
934.8778_8.32	TG(56:0)	0.11408	0.00496	0.11734	0.00997	0.12050	0.00511	1.03	0.12644	0.00385	1.08	0.11271	0.01363	0.96	0.12273	0.00487	1.05
932.8621_8.2	TG(56:2)	0.04394	0.00246	0.04452	0.00179	0.04505	0.00176	1.01	0.04712	0.00092	1.06	0.04655	0.00401	1.05	0.04395	0.00309	0.99
913.8172_8.32	TG(56:3)	0.00434	0.00021	0.00580	0.00121	0.00538	0.00073	0.93	0.00663	0.00108	1.14	0.00559	0.00251	0.96	0.00565	0.00152	0.97
928.8301_7.89	TG(56:4)	0.02626	0.00444	0.02756	0.00477	0.02614	0.00542	0.95	0.02569	0.00211	0.93	0.04149	0.03308	1.51	• 0.02617	0.00307	0.95 •
926.8112_7.48	TG(56:5)	0.00285	0.00031	0.00287	0.00027	0.00291	0.00039	1.01	0.00334	0.00045	1.16	0.00272	0.00104	0.95	0.00303	0.00030	1.05
907.7817_6.65	TG(56:6)	0.00256	0.00044	0.00282	0.00067	0.00259	0.00083	0.92	0.00305	0.00052	1.08	0.00247	0.00043	0.88	0.00280	0.00044	0.99
924.8018_7.67	TG(56:6)	0.05953	0.01359	0.06668	0.01711	0.06022	0.02079	0.90	0.05303	0.01016	0.80	0.10034	0.09363	1.50	• 0.06236	0.01075	0.94 •
922.7852_7.59	TG(56:7)	0.03003	0.00886	0.03691	0.01431	0.02650	0.01245	0.72	0.01832	0.00700	0.50	0.04799	0.05038	1.30	0.03341	0.00808	0.91
920.7695_7.43	TG(56:8)	0.14125	0.03570	0.15568	0.02958	0.15011	0.06008	0.96	0.13073	0.03094	0.84	0.18027	0.08843	1.16	0.16080	0.04100	1.03
918.7549_7.20	TG(56:9)	0.03209	0.00914	0.03745	0.00807	0.03247	0.01311	0.87	0.02822	0.00648	0.75	0.03561	0.01125	0.95	0.03834	0.01064	1.02
916.7409_7.05	TG(56:10)	0.01224	0.00278	0.01319	0.00272	0.01288	0.00356	0.98	0.01204	0.00197	0.91	0.01292	0.00289	0.98	0.01380	0.00300	1.05
946.8773_7.87	TG(57:2)	0.05062	0.00822	0.04781	0.00372	0.04544	0.00647	0.95	0.05340	0.00497	1.12	0.05144	0.00667	1.08	0.04180	0.00950	0.87
944.8624_8.14	TG(57:3)	0.00902	0.00054	0.00922	0.00071	0.00980	0.00048	1.06	0.01027	0.00065	1.11	0.01256	0.00699	1.36	0.00976	0.00121	1.06
944.8632_7.71	TG(57:3)	0.02204	0.00409	0.02271	0.00285	0.01852	0.00425	0.82	0.02222	0.00277	0.98	0.02588	0.01079	1.14	0.01882	0.00669	0.83
942.8471_7.56	TG(57:4)	0.02166	0.00480	0.02241	0.00301	0.01865	0.00579	0.83	0.02042	0.00352	0.91	0.02345	0.00986	1.05	0.01992	0.00668	0.89
940.8291_7.41	TG(57:5)	0.00664	0.00204	0.00676	0.00143	0.00679	0.00283	1.00	0.00695	0.00111	1.03	0.00753	0.00320	1.11	0.00676	0.00199	1.00
938.8148_7.76	TG(57:6)	0.00054	0.00028	0.00067	0.00019	0.00080	0.00014	1.19	0.00061	0.00020	0.91	0.00080	0.00058	1.20	0.00076	0.00024	1.14
936.8054_7.67	TG(57:7)	0.00174	0.00038	0.00210	0.00033	0.00172	0.00061	0.82	0.00217	0.00035	1.04	0.00234	0.00113	1.11	0.00208	0.00040	0.99

934.7865_7.52	TG(57:8)	0.00466	0.00106	0.00572	0.00124	0.00583	0.00159	1.02	0.00560	0.00073	0.98	0.00639	0.00234	1.12	0.00607	0.00085	1.06
932.7702_7.27	TG(57:9)	0.00436	0.00088	0.00519	0.00055	0.00546	0.00107	1.05	0.00551	0.00068	1.06	0.00562	0.00142	1.08	0.00584	0.00074	1.13
913.7242_7.41	TG(57:10)	0.00078	0.00010	0.00082	0.00010	0.00072	0.00030	0.87	0.00084	0.00020	1.03	0.00084	0.00031	1.02	0.00091	0.00033	1.11
928.7461_7.59	TG(57:11)	0.00937	0.00234	0.00999	0.00240	0.00940	0.00379	0.94	0.00818	0.00151	0.82	0.01217	0.00703	1.22	0.01068	0.00212	1.07
931.6793_6.67	TG(57:12)	0.00146	0.00019	0.00165	0.00014	0.00186	0.00042	1.13	0.00188	0.00026	1.14	0.00161	0.00032	0.98	0.00166	0.00042	1.01
960.8938_8.35	TG(58:2)	0.03454	0.00180	0.03475	0.00219	0.03642	0.00112	1.05	0.03855	0.00204	1.11	0.03633	0.00140	1.05	0.03576	0.00176	1.03
960.8967_7.87	TG(58:2)	0.01315	0.00270	0.01280	0.00117	0.01201	0.00184	0.94	0.01399	0.00163	1.09	0.01359	0.00173	1.06	0.01093	0.00273	0.85
958.8776_8.2	TG(58:3)	0.01400	0.00147	0.01399	0.00074	0.01414	0.00100	1.01	0.01479	0.00034	1.06	0.01779	0.00717	1.27	0.01427	0.00134	1.02
958.8782_7.71	TG(58:3)	0.00845	0.00145	0.00876	0.00113	0.00751	0.00112	0.86	0.00927	0.00133	1.06	0.00950	0.00262	1.09	0.00737	0.00277	0.84
956.8618_8.06	TG(58:4)	0.00637	0.00109	0.00733	0.00151	0.00664	0.00159	0.91	0.00652	0.00096	0.89	0.01068	0.00861	1.46	0.00631	0.00096	0.86
954.8420_7.41	TG(58:5)	0.00267	0.00107	0.00254	0.00054	0.00270	0.00138	1.06	0.00277	0.00099	1.09	0.00302	0.00168	1.19	0.00249	0.00093	0.98
954.8437_7.89	TG(58:5)	0.00688	0.00129	0.00806	0.00113	0.00775	0.00163	0.96	0.00722	0.00119	0.90	0.00958	0.00552	1.19	0.00738	0.00085	0.91
950.8127_7.61	TG(58:7)	0.02258	0.00516	0.02670	0.00812	0.02374	0.00917	0.89	0.02145	0.00523	0.80	0.03383	0.02543	1.27	0.02518	0.00439	0.94
950.9093_7.81	TG(58:7)	0.00061	0.00011	0.00065	0.00011	0.00079	0.00007	1.23	0.00083	0.00020	1.28	0.00071	0.00023	1.10	0.00071	0.00016	1.10
931.7702_7.74	TG(58:8)	0.00852	0.00190	0.00857	0.00147	0.00836	0.00227	0.98	0.00809	0.00092	0.94	0.01126	0.00685	1.31	0.00836	0.00098	0.98
946.7835_7.45	TG(58:9)	0.06883	0.02113	0.08291	0.02185	0.08127	0.03616	0.98	0.06825	0.01941	0.82	0.10170	0.05843	1.23	0.08746	0.02202	1.05
944.7656_7.63	TG(58:10)	0.00044	0.00014	0.00055	0.00009	0.00049	0.00015	0.90	0.00048	0.00011	0.88	0.00101	0.00114	1.83	0.00059	0.00014	1.07
944.7687_7.28	TG(58:10)	0.04119	0.01356	0.05296	0.00778	0.05473	0.02102	1.03	0.04272	0.01182	0.81	0.05782	0.02411	1.09	0.06080	0.01958	1.15
972.8929_7.88	TG(59:3)	0.03136	0.00528	0.03068	0.00307	0.02884	0.00364	0.94	0.03251	0.00351	1.06	0.03194	0.00446	1.04	0.02573	0.00680	0.84
970.8774_8.14	TG(59:4)	0.00154	0.00021	0.00175	0.00031	0.00189	0.00060	1.08	0.00180	0.00037	1.03	0.00288	0.00211	1.65	0.00184	0.00036	1.06
970.8778_7.73	TG(59:4)	0.01530	0.00325	0.01633	0.00212	0.01364	0.00337	0.84	0.01569	0.00222	0.96	0.01802	0.00735	1.10	0.01362	0.00525	0.83
968.8579_7.92	TG(59:5)	0.00198	0.00046	0.00225	0.00028	0.00225	0.00053	1.00	0.00238	0.00041	1.06	0.00342	0.00259	1.52	0.00190	0.00036	0.85
968.8627_7.56	TG(59:5)	0.02122	0.00549	0.02330	0.00398	0.02012	0.00674	0.86	0.02207	0.00365	0.95	0.02352	0.00975	1.01	0.02051	0.00758	0.88
966.8475_7.41	TG(59:6)	0.01806	0.00537	0.01812	0.00385	0.01790	0.00722	0.99	0.01939	0.00302	1.07	0.02021	0.00876	1.12	0.01802	0.00661	0.99
964.8353_7.25	TG(59:7)	0.00697	0.00190	0.00675	0.00113	0.00738	0.00197	1.09	0.00751	0.00098	1.11	0.00758	0.00261	1.12	0.00671	0.00188	0.99
935.7183_7.04	TG(59:13)	0.00266	0.00025	0.00280	0.00040	0.00305	0.00025	1.09	0.00336	0.00025	1.20	0.00298	0.00056	1.07	0.00323	0.00059	1.15
1000.9265_8.04	TG(60:1)	0.00303	0.00051	0.00322	0.00038	0.00322	0.00081	1.00	0.00398	0.00054	1.24	0.00401	0.00148	1.25	0.00300	0.00080	0.93
986.9087_7.88	TG(60:3)	0.00766	0.00145	0.00771	0.00092	0.00687	0.00066	0.89	0.00802	0.00086	1.04	0.00745	0.00126	0.97	0.00609	0.00206	0.79
984.8931_7.73	TG(60:4)	0.00392	0.00106	0.00404	0.00072	0.00309	0.00072	0.76	0.00389	0.00075	0.96	0.00429	0.00198	1.06	0.00318	0.00171	0.79
982.875_8.06	TG(60:5)	0.00385	0.00050	0.00444	0.00039	0.00424	0.00033	0.95	0.00442	0.00059	1.00	0.00506	0.00198	1.14	0.00425	0.00073	0.96
982.8786_7.58	TG(60:5)	0.00471	0.00147	0.00521	0.00132	0.00437	0.00169	0.84	0.00497	0.00103	0.95	0.00514	0.00242	0.99	0.00440	0.00204	0.84
980.8636_7.41	TG(60:6)	0.00341	0.00161	0.00344	0.00097	0.00347	0.00190	1.01	0.00396	0.00084	1.15	0.00376	0.00240	1.09	0.00339	0.00179	0.99
978.8475_7.25	TG(60:7)	0.00097	0.00060	0.00064	0.00038	0.00081	0.00053	1.28	0.00101	0.00032	1.58	0.00087	0.00072	1.37	0.00056	0.00051	0.88
976.8321_7.78	TG(60:8)	0.00306	0.00103	0.00494	0.00179	0.00461	0.00145	0.93	0.00380	0.00108	0.77	0.00562	0.00449	1.14	0.00415	0.00085	0.84
974.8151_7.61	TG(60:9)	0.00746	0.00266	0.00963	0.00333	0.00895	0.00392	0.93	0.00705	0.00178	0.73	0.01098	0.00803	1.14	0.01053	0.00274	1.09
998.907_7.91	TG(61:4)	0.00070	0.00051	0.00102	0.00038	0.00078	0.00055	0.76	0.00062	0.00026	0.60	0.00148	0.00157	1.44	0.00046	0.00037	0.45
992.8649_7.59	TG(61:7)	0.00196	0.00097	0.00215	0.00053	0.00190	0.00124	0.88	0.00141	0.00052	0.66	0.00285	0.00276	1.33	0.00182	0.00106	0.85
988.8400_7.20	TG(61:9)	0.00156	0.00045	0.00172	0.00047	0.00165	0.00061	0.96	0.00136	0.00030	0.79	0.00152	0.00046	0.88	0.00159	0.00085	0.93
1004.8645_7.43	TG(62:8)	0.00050	0.00013	0.00050	0.00020	0.00041	0.00030	0.82	0.00032	0.00015	0.64	0.00056	0.00048	1.14	0.00045	0.00034	0.91
953.7559_7.61	TG(60:11)	0.00584	0.00175	0.00700	0.00252	0.00642	0.00329	0.92	0.00544	0.00148	0.78	0.00784	0.00498	1.12	0.00693	0.00182	0.99
970.7852_7.38	TG(60:11)	0.01247	0.00488	0.01917	0.00563	0.01703	0.00620	0.89	0.01088	0.00436	0.57	0.01819	0.01274	0.95	0.02092	0.00788	1.09
937.7624_7.4	TG(60:12)	0.00132	0.00019	0.00141	0.00021	0.00139	0.00066	0.99	0.00152	0.00055	1.08	0.00156	0.00078	1.11	0.00159	0.00034	1.13
968.7694_7.22	TG(60:12)	0.03150	0.01363	0.04544	0.00803	0.04656	0.01627	1.02	0.02792	0.00987	0.61	0.03696	0.01653	0.81	0.05465	0.02338	1.20
994.7850_7.31	TG(62:13)	0.01057	0.00428	0.01589	0.00679	0.01477	0.00708	0.93	0.00909	0.00412	0.57	0.01445	0.00923	0.91	0.01690	0.00773	1.06
992.7691_7.13	TG(62:14)	0.01811	0.00645	0.02265	0.00553	0.02326	0.00842	1.03	0.01586	0.00496	0.70	0.01957	0.00863	0.86	0.02613	0.01168	1.15
1016.8628_7.45	TG(63:9)	0.00112	0.00065	0.00137	0.00046	0.00150	0.00097	1.09	0.00104	0.00040	0.76	0.00167	0.00131	1.22	0.00153	0.00078	1.12
1014.8462_7.28	TG(63:10)	0.00094	0.00059	0.00142	0.00032	0.00174	0.00100	1.22	0.00122	0.00063	0.85	0.00175	0.00112	1.23	0.00165	0.00093	1.16

Supplementary Table 3: Triacylglyceride levels Day 7 with fold changes of neutron contribution compared to photons.

m/z_Ret time	ID	C_D7		0_D7		5_D7		Fold change		15_D7		Fold change		25_D7		Fold change		83_D7		Fold change	
		Average	SD	Average	SD	Average	SD	to X-ray (0%)		Average	SD	to X-ray (0%)		Average	SD	to X-ray (0%)		Average	SD	to X-ray (0%)	
684.6134_6.79	TG(38:0)	0.03853	0.00291	0.04111	0.00185	0.04170	0.00216	1.01		0.04319	0.00437	1.05		0.04563	0.00391	1.11		0.03830	0.00225	0.93	
712.6447_7.04	TG(40:0)	0.04094	0.00249	0.03906	0.00205	0.04159	0.00212	1.06		0.04340	0.00501	1.11		0.04694	0.00290	1.20		0.03678	0.00225	0.94	
740.6753_7.27	TG(42:0)	0.04177	0.00217	0.04410	0.00216	0.04489	0.00192	1.02		0.04585	0.00502	1.04		0.04874	0.00295	1.11		0.04149	0.00248	0.94	
738.6602_7.07	TG(42:1)	0.02129	0.00138	0.01993	0.00099	0.02097	0.00096	1.05		0.02187	0.00240	1.10		0.02468	0.00153	1.24		0.01908	0.00117	0.96	
752.6753_7.18	TG(43:1)	0.01793	0.00076	0.01772	0.00068	0.01822	0.00079	1.03		0.01910	0.00194	1.08		0.02065	0.00116	1.17		0.01682	0.00092	0.95	
768.7061_7.48	TG(44:0)	0.15049	0.00437	0.14275	0.00721	0.15265	0.00328	1.07		0.15743	0.01492	1.10		0.17193	0.00790	1.20		0.14034	0.00810	0.98	
754.6905_7.36	TG(44:1)	0.02953	0.00131	0.02845	0.00146	0.03004	0.00178	1.06		0.03108	0.00333	1.09		0.03413	0.00177	1.20		0.02684	0.00156	0.94	
766.6907_7.3	TG(44:1)	0.05233	0.00154	0.05156	0.00268	0.05400	0.00205	1.05		0.05644	0.00590	1.09		0.06084	0.00409	1.18		0.04910	0.00268	0.95	
764.6761_7.12	TG(44:2)	0.01583	0.00076	0.01599	0.00104	0.01642	0.00080	1.03		0.01699	0.00164	1.06		0.01816	0.00110	1.14		0.01492	0.00070	0.93	
762.6620_6.93	TG(44:3)	0.00178	0.00021	0.00181	0.00018	0.00192	0.00028	1.06		0.00219	0.00034	1.21		0.00227	0.00025	1.26		0.00158	0.00018	0.88	
782.7216_7.56	TG(45:0)	0.13021	0.00344	0.11874	0.00625	0.12929	0.00233	1.09		0.13510	0.01008	1.14		0.14429	0.00696	1.22		0.11833	0.00738	1.00	
780.7066_7.4	TG(45:1)	0.07416	0.00260	0.06768	0.00343	0.07275	0.00254	1.07		0.07589	0.00726	1.12		0.08320	0.00461	1.23		0.06532	0.00329	0.97	
778.6909_7.23	TG(45:2)	0.01712	0.00097	0.01634	0.00086	0.01700	0.00083	1.04		0.01780	0.00160	1.09		0.01965	0.00115	1.20		0.01537	0.00093	0.94	
796.7375_7.67	TG(46:0)	0.20941	0.00413	0.19832	0.00771	0.21625	0.00625	1.09		0.22130	0.01603	1.12		0.23627	0.01052	1.19		0.19808	0.01136	1.00	
794.7222_7.51	TG(46:1)	0.27050	0.00664	0.25071	0.01474	0.26683	0.00554	1.06		0.27814	0.02546	1.11		0.30512	0.01398	1.22		0.24452	0.01296	0.98	
792.7064_7.34	TG(46:2)	0.06688	0.00247	0.06374	0.00241	0.06921	0.00423	1.09		0.07122	0.00687	1.12		0.07723	0.00485	1.21		0.06161	0.00284	0.97	
790.691_7.18	TG(46:3)	0.00909	0.00045	0.00920	0.00055	0.00961	0.00054	1.04		0.01025	0.00081	1.11		0.01117	0.00062	1.21		0.00881	0.00033	0.96	
808.7380_7.59	TG(47:1)	0.29052	0.00655	0.26714	0.01276	0.28743	0.00531	1.08		0.29927	0.02303	1.12		0.32303	0.01518	1.21		0.26471	0.01445	0.99	
818.7224_7.38	TG(47:1)	0.02294	0.00094	0.02064	0.00102	0.02301	0.00128	1.11		0.02592	0.00296	1.26		0.02863	0.00110	1.39		0.02114	0.00084	1.02	
806.7227_7.43	TG(47:2)	0.06395	0.00234	0.06122	0.00378	0.06447	0.00173	1.05		0.06783	0.00679	1.11		0.07396	0.00368	1.21		0.06030	0.00257	0.98	
804.7066_7.28	TG(47:3)	0.01081	0.00061	0.01067	0.00053	0.01124	0.00044	1.05		0.01159	0.00109	1.09		0.01242	0.00101	1.16		0.01017	0.00074	0.95	
824.6723_7.85	TG(48:0)	0.00921	0.00032	0.00926	0.00055	0.01012	0.00018	1.09		0.00982	0.00127	1.06		0.00891	0.00031	0.96		0.00900	0.00059	0.97	
822.7534_7.7	TG(48:1)	0.40014	0.00743	0.36952	0.01457	0.40284	0.00968	1.09		0.42127	0.03231	1.14		0.45396	0.02196	1.23		0.37160	0.02005	1.01	
832.738_7.48	TG(48:1)	0.02492	0.00063	0.02335	0.00154	0.02552	0.00115	1.09		0.02726	0.00300	1.17		0.03034	0.00082	1.30		0.02385	0.00072	1.02	
820.7382_7.55	TG(48:2)	0.17211	0.00337	0.15617	0.00633	0.17498	0.00554	1.12		0.18747	0.01476	1.20		0.20034	0.00800	1.28		0.16181	0.00554	1.04	
816.706_7.16	TG(48:4)	0.00491	0.00057	0.00495	0.00043	0.00563	0.00067	1.14		0.00673	0.00054	1.36		0.00718	0.00038	1.45	•	0.00523	0.00019	1.06	
838.7821_7.49	TG(49:0)	0.02553	0.00217	0.02515	0.00331	0.02416	0.00271	0.96		0.02691	0.00332	1.07		0.02981	0.00162	1.19		0.02293	0.00324	0.91	
838.7821_7.92	TG(49:0)	0.15155	0.00460	0.14100	0.00678	0.15447	0.00419	1.10		0.15458	0.01019	1.10		0.16134	0.00889	1.14		0.13813	0.00946	0.98	
813.6944_6.67	TG(49:4)	0.00225	0.00029	0.00239	0.00030	0.00258	0.00023	1.08		0.00278	0.00028	1.16		0.00292	0.00032	1.22		0.00238	0.00029	0.99	
830.7187_7.3	TG(49:4)	0.00210	0.00032	0.00204	0.00024	0.00213	0.00033	1.04		0.00270	0.00046	1.32		0.00316	0.00018	1.55	•	0.00233	0.00034	1.14	
822.6553_7.72	TG(49:8)	0.00159	0.00023	0.00171	0.00024	0.00177	0.00019	1.03		0.00213	0.00038	1.25		0.00240	0.00021	1.40		0.00175	0.00034	1.02	
852.7989_8.02	TG(50:0)	0.13341	0.00250	0.13434	0.00692	0.14734	0.00477	1.10		0.14368	0.00724	1.07		0.14707	0.00759	1.09		0.13486	0.00861	1.00	
850.785_7.87	TG(50:1)	0.47981	0.01389	0.44787	0.02263	0.49646	0.01493	1.11		0.52549	0.04652	1.17		0.55157	0.03081	1.23		0.46532	0.01878	1.04	
848.7694_7.71	TG(50:2)	0.33039	0.00968	0.29710	0.00989	0.34987	0.02936	1.18		0.41753	0.04325	1.41		0.43115	0.02048	1.45		0.36144	0.05174	1.22	
848.7698_7.22	TG(50:2)	0.00064	0.00008	0.00049	0.00016	0.00073	0.00013	1.49		0.00077	0.00019	1.58	•	0.00101	0.00006	2.07	•	0.00075	0.00012	1.54	•
846.7536_7.56	TG(50:3)	0.08570	0.00487	0.07328	0.00454	0.09061	0.01197	1.24		0.12077	0.01791	1.65	•	0.12456	0.00587	1.70	•	0.09436	0.01492	1.29	
844.7376_7.37	TG(50:4)	0.01037	0.00162	0.00891	0.00112	0.01354	0.00374	1.52		0.02130	0.00352	2.39	•	0.02146	0.00145	2.41	•	0.01348	0.00284	1.51	•
825.6935_7.53	TG(50:5)	0.03023	0.00149	0.02879	0.00164	0.02916	0.00171	1.01		0.02994	0.00336	1.04		0.03246	0.00184	1.13		0.02649	0.00190	0.92	
842.7155_7.20	TG(50:5)	0.00890	0.00098	0.00870	0.00024	0.01070	0.00191	1.23		0.01448	0.00165	1.66		0.01502	0.00092	1.73	•	0.00968	0.00107	1.11	
823.6781_7.37	TG(50:6)	0.01026	0.00058	0.00978	0.00064	0.01009	0.00032	1.03		0.01080	0.00097	1.10		0.01168	0.00083	1.19		0.00918	0.00055	0.94	
862.7845_7.79	TG(51:2)	0.23136	0.00573	0.22448	0.01002	0.24098	0.00679	1.07		0.24484	0.01412	1.09		0.25542	0.01342	1.14		0.22383	0.01000	1.00	
860.7690_7.66	TG(51:3)	0.03526	0.00097	0.03227	0.00126	0.03720	0.00278	1.15		0.04288	0.00369	1.33		0.04554	0.00182	1.41		0.03709	0.00279	1.15	
865.72_7.64	TG(51:3)	0.00898	0.00032	0.00867	0.00026	0.00976	0.00088	1.13		0.01110	0.00062	1.28		0.01129	0.00066	1.30		0.00931	0.00044	1.07	
858.7526_7.48	TG(51:4)	0.00799	0.00044	0.00721	0.00050	0.00945	0.00204	1.31		0.01247	0.00187	1.73	•	0.01333	0.00035	1.85	•	0.00932	0.00153	1.29	
863.7064_7.46	TG(51:4)	0.00322	0.00033	0.00275	0.00029	0.00334	0.00077	1.22		0.00418	0.00043	1.52	•	0.00416	0.00050	1.51	•	0.00317	0.00028	1.15	
861.6860_7.31	TG(51:5)	0.00323	0.00042	0.00382	0.00035	0.00419	0.00084	1.10		0.00527	0.00074	1.38		0.00541	0.00070	1.41		0.00405	0.00047	1.06	
880.8308_8.17	TG(52:0)	0.07611	0.00150	0.07883	0.00722	0.08715	0.00345	1.11		0.08456	0.00444	1.07		0.08460	0.00448	1.07		0.08314	0.00434	1.05	
878.8152_8.03	TG(52:1)	0.15978	0.00312	0.15441	0.00587	0.17166	0.00519	1.11		0.17561	0.01007	1.14		0.18177	0.00884	1.18		0.16744	0.01495	1.08	
876.8008_7.87	TG(52:2)	0.42219	0.01325	0.38380	0.01500	0.44917	0.03279	1.17		0.54162	0.07541	1.41		0.54068	0.03469	1.41		0.47644	0.08904	1.24	
874.7852_7.71	TG(52:3)	0.31158	0.04769	0.24197	0.02317	0.36664	0.10684	1.52		0.56190	0.10315	2.32	•	0.54787	0.04790	2.26	•	0.48658	0.22108	2.01	•
872.7695_7.56	TG(52:4)	0.23050	0.05499	0.15189	0.02582	0.28848	0.11696	1.90		0.48048	0.09839	3.16	•	0.48197	0.05269	3.17	•	0.33701	0.13008	2.22	•
853.7274_6.77	TG(52:5)	0.00340	0.00036	0.00399	0.00064	0.00415	0.00086	1.04		0.00545	0.00101	1.37	•	0.00632	0.00114	1.58	•	0.00343	0.00056	0.86	
870.7506_7.41	TG(52:5)	0.06314	0.01124	0.04938	0.00638	0.08025	0.02573	1.63		0.12689	0.02302	2.57	•	0.12772	0.01081	2.59	•	0.07963	0.01948	1.61	•
868.7359_7.23	TG(52:6)	0.02116	0.00247	0.01908	0.00182	0.02613	0.00616	1.37		0.03708	0.00515	1.94	•	0.03757	0.00277	1.97	•	0.02395	0.00313	1.26	
849.6938_7.37	TG(52:7)	0.00530	0.00087	0.00463	0.00062	0.00726	0.00185	1.57	•	0.01055	0.00178	2.28	•	0.01007	0.00067	2.18		0.00697	0.00143	1.51	•
866.7223_7.13	TG(52:7)	0.00149	0.00038	0.00152	0.00030	0.00210	0.00056	1.38		0.00307	0.00039	2.02	•	0.00321	0.00050	2.12		0.00213	0.00047	1.41	
866.7232_7.07	TG(52:7)	0.00184	0.00027	0.00172	0.00046	0.00252	0.00054	1.46		0.00346	0.00047	2.01	•	0.00359	0.00039	2.08		0.00206	0.00031	1.20	
871.6738_7.13	TG(52:7)	0.00198	0.00019	0.00148	0.00032	0.00238	0.00072	1.61	•	0.00351	0.00057	2.37	•	0.00341	0.00034	2.31	•	0.00210	0.00056	1.42	
894.7528_7.25	TG(52:7)	0.08993	0.01486	0.07570	0.01072	0.11036	0.03322	1.46		0.16311	0.02744	2.15	•	0.16308	0.01178	2.15	•	0.11157	0.02069	1.47	
864.7126_6.92	TG(52:8)	0.00243	0.00030	0.00226	0.00007	0.00273	0.00072	1.21		0.00391	0.00046	1.73	•	0.00395	0.00044	1.75	•	0.00215	0.00016	0.95	
890.8148_7.96	TG(53:2)	0.10151	0.00258	0.09677	0.00458	0.10581	0.00220	1.09		0.10618	0.00626	1.10		0.11121	0.00568	1.15		0.09745	0.00477	1.01	
888.7989_7.37	TG(53:3)	0.00322	0.00049	0.00296	0.00013	0.00320	0.00058	1.08		0.00348	0.00044	1.18		0.00397	0.00032	1.34		0.00320	0.00042	1.08	
888.7991_7.81	TG(53:3)	0.02685	0.00093	0.02527	0.00104	0.02941	0.00244	1.16		0.03334	0.00230	1.32		0.03416	0.00147	1.35		0.02920	0.00284	1.16	
886.7846_7.66	TG(53:4)	0.00985	0.00097	0.00814	0.00037	0.01159	0.00241	1.43		0.01503	0.00211	1.85	•	0.01583	0.00144	1.95	•	0.01272	0.00331	1.56	•
882.7614_6.92	TG(53:6)	0.00295	0.00050	0.00322	0.00024	0.00347	0.00062	1.08		0.00444	0.00090	1.38		0.00461	0.00054	1.43		0.00319	0.00035	0.99	
876.7090_7.88	TG(53:9)	0.00168	0.00036	0.00132	0.00017	0.00188	0.00040	1.42		0.00276	0.00083	2.09	•	0.00233	0.00025	1.77	•	0.00287	0.00189	2.17	
906.8469_8.18	TG(54:1)	0.10863	0.00221	0.10909	0.00525	0.11846	0.00406	1.09		0.11719	0.00571	1.07		0.11803	0.00606	1.08		0.10944	0.00399	1.00	
904.8307_8.03	TG(54:2)	0.12484	0.00138	0.12041	0.00400	0.13505	0.00609	1.12		0.14220	0.00680	1.18		0.14427	0.00728	1.20		0.13773	0.01968	1.14	
902.8163_7.88	TG(54:3)	0.34101	0.01007	0.31487	0.01233	0.36629	0.02372	1.16		0.40460	0.02924	1.28		0.41204	0.02301	1.31		0.37332	0.05825	1.19	
902.8238_7.46	TG(54:3)	0.01042	0.00116	0.00964	0.00164	0.00967	0.00102	1.00		0.01152	0.00149	1.20		0.01239	0.00088	1.29		0.00949	0.00180	0.98	
900.8009_7.73	TG(54:4)	0.20972	0.02523	0.17458	0.00970	0.26161	0.06265	1.50	•	0.36832	0.05904	2.11	•	0.36244	0.02766	2.08	•	0.31609	0.13237	1.81	
898.7852_7.56	TG(54:5)	0.24918	0.04687	0.18555	0.02083	0.32798	0.10352	1.77	•	0.52691	0.10732	2.84	•	0.51938	0.04499	2.80	•	0.38085	0.17395	2.05	•
879.7443_6.71	TG(54:6)	0.00961	0.00126	0.01079	0.00086	0.01207	0.00128	1.12		0.01448	0.00191	1.34		0.01553	0.00181	1.44		0.01065	0.00089	0.99	
896.7684_7.41	TG(54:6)	0.26191	0.04379	0.20428	0.02198	0.33260	0.10023	1.63	•	0.51775	0.09883	2.53	•	0.52289	0.03836	2.56	•	0.33109	0.08761	1.62	•
877.7298_6.56	TG(54:7)	0.00285	0.00043	0.00279	0.00028	0.00369	0.00050	1.32		0.00435	0.00044	1.56	•	0.00470	0.00067	1.68	•	0.00281	0.00032	1.01	
892.7406_7.08	TG(54:8)	0.02075	0.00321	0.01777	0.00322	0.02553	0.00671	1.44		0.03690	0.00557	2.08	•	0.03695	0.00321	2.08	•	0.02441	0.00417	1.37	
920.8618_7.87	TG(55:1)	0.06225	0.00274	0.05906	0.00660	0.06293	0.00746	1.07		0.06351	0.00866	1.08		0.07255	0.00612	1.23		0.05590	0.00658	0.95	
918.8466_8.12	TG(55:2)	0.02784	0.00074	0.02700	0.00123	0.02939	0.00091	1.09		0.02960	0.00198	1.10		0.03075	0.00172	1.14		0.02860	0.00131	1.06	
908.7723_7.43	TG(55:5)	0.00280	0.00055	0.00211	0.00027	0.00297	0.00157	1.41		0.00499	0.00099	2.37	•	0.00500	0.00067	2.37	•	0.00465	0.00114	2.21	•
910.782_7.59	TG(55:6)	0.00502	0.00064	0.00467	0.00030	0.00603	0.00141	1.29		0.00776	0.00097	1.66	•	0.00773	0.00056	1.65	•	0.00647	0.00109	1.38	
891.7352_7.64	TG(55:7)	0.00282	0.00018	0.00282	0.00014	0.00328	0.00054	1.17		0.00384	0.00013	1.36		0.00392	0.00012	1.39		0.00350	0.00081	1.24	
889.7236_7.48	TG(55:8)	0.00120	0.00033	0.00114	0.00013	0.00178	0.00056	1.57	•	0.00213	0.00034	1.87	•	0.00233	0.00012	2.05	•	0.00152	0.00021	1.33	
906.7594_7.33	TG(55:8)	0.00209	0.00051	0.00196	0.00040	0.00265	0.00134	1.35		0.00385	0.00046	1.97	•	0.00388	0.00064	1.98	•	0.00299	0.00032	1.53	•
887.7038_7.10	TG(55:9)	0.00090	0.00015	0.00120	0.00043	0.00144	0.00046	1.20		0.00184	0.00020	1.53	•	0.00180	0.00015	1.50	•	0.00121	0.00013	1.01	
887.7120_7.33	TG(55:9)	0.00324	0.00033	0.00308	0.00030	0.00340	0.00068	1.10		0.00413	0.00049	1.34		0.00441	0.00039	1.43		0.00335	0.00021	1.09	
934.8778_8.32	TG(56:0)	0.11246	0.00226	0.11218	0.00601	0.12089	0.00298	1.08		0.11955	0.00567	1.07		0.12016	0.00589	1.07		0.10874	0.00681	0.97	
932.8621_8.2	TG(56:2)	0.04242	0.00085	0.04117	0.00208	0.04619	0.00171	1.12		0.04706	0.00171	1.14		0.04774	0.00229	1.16		0.04496	0.00277	1.09	
913.8172_8.32	TG(56:3)	0.00540	0.00021	0.00547	0.00077	0.00595	0.00045	1.09		0.00552	0.00051	1.01		0.00487	0.00019	0.89		0.00659	0.00114	1.21	
928.8301_7.89	TG(56:4)	0.01988	0.00217	0.01829	0.00163	0.02445	0.00401	1.34		0.03139	0.00416	1.72	•	0.02996	0.00509	1.64	•	0.03583	0.01909	1.96	•
926.8112_7.48	TG(56:5)	0.00245	0.00016	0.00216	0.00054	0.00258	0.00041	1.19		0.00288	0.00034	1.33		0.00300	0.00020	1.39		0.00231	0.00038	1.07	
907.7817_6.65	TG(56:6)	0.00224	0.00026	0.00242	0.00027	0.00240	0.00024	0.99		0.00281	0.00051	1.16		0.00317	0.00039	1.31		0.00222	0.00041	0.92	
924.8018_7.67	TG(56:6)	0.03957	0.00768	0.03106	0.00597	0.05100	0.01791	1.64	•	0.08003	0.01588	2.58	•	0.07546	0.01111	2.43	•	0.09532	0.06441	3.07	•
922.7852_7.59	TG(56:7)	0.01691	0.00700	0.00864	0.00494	0.02290	0.01511	2.65	•	0.04237	0.01061	4.90	•	0.04089	0.00902	4.73	•	0.06156	0.05124	7.12	•
920.7695_7.43	TG(56:8)	0.09936	0.03300	0.05909	0.02636	0.10799	0.06701	1.83	•	0.16934	0.03399	2.87	•	0.17606	0.03159	2.98	•	0.17200	0.06767	2.91	•

918.7549_7.20	TG(56:9)	0.02186	0.00817	0.01405	0.00857	0.02554	0.01614	1.82	•	0.04136	0.00993	2.94	•	0.04176	0.00746	2.97	•	0.03649	0.01152	2.60	•
916.7409_7.05	TG(56:10)	0.01077	0.00265	0.00795	0.00298	0.01036	0.00435	1.30		0.01458	0.00224	1.83	•	0.01515	0.00230	1.91	•	0.01239	0.00288	1.56	•
946.8773_7.87	TG(57:2)	0.04742	0.00318	0.04351	0.00577	0.04862	0.00669	1.12		0.05450	0.00657	1.25		0.05968	0.00495	1.37		0.04710	0.00224	1.08	
944.8624_8.14	TG(57:3)	0.00867	0.00061	0.00865	0.00041	0.00944	0.00059	1.09		0.00973	0.00070	1.13		0.00998	0.00081	1.15		0.01245	0.00494	1.44	
944.8632_7.71	TG(57:3)	0.01957	0.00303	0.01518	0.00282	0.02194	0.00608	1.44		0.02866	0.00519	1.89	•	0.02731	0.00252	1.80	•	0.02497	0.00571	1.64	•
942.8471_7.56	TG(57:4)	0.01510	0.00299	0.01146	0.00292	0.01906	0.00707	1.66	•	0.02877	0.00619	2.51	•	0.02762	0.00303	2.41	•	0.02105	0.00325	1.84	•
940.8291_7.41	TG(57:5)	0.00445	0.00101	0.00324	0.00110	0.00533	0.00240	1.65	•	0.00892	0.00108	2.75	•	0.00913	0.00143	2.82	•	0.00584	0.00081	1.80	•
938.8148_7.76	TG(57:6)	0.00047	0.00009	0.00031	0.00013	0.00054	0.00022	1.75	•	0.00092	0.00030	2.97	•	0.00091	0.00013	2.92	•	0.00073	0.00031	2.34	•
936.8054_7.67	TG(57:7)	0.00135	0.00022	0.00108	0.00022	0.00178	0.00068	1.64	•	0.00231	0.00064	2.13	•	0.00241	0.00035	2.23	•	0.00247	0.00091	2.28	•
934.7865_7.52	TG(57:8)	0.00370	0.00072	0.00333	0.00067	0.00449	0.00170	1.35		0.00593	0.00088	1.78	•	0.00607	0.00061	1.82	•	0.00557	0.00183	1.67	•
932.7702_7.27	TG(57:9)	0.00359	0.00043	0.00389	0.00035	0.00466	0.00111	1.20		0.00572	0.00041	1.47		0.00597	0.00039	1.54	•	0.00508	0.00063	1.31	
913.7242_7.41	TG(57:10)	0.00050	0.00022	0.00055	0.00005	0.00071	0.00027	1.30		0.00106	0.00020	1.92	•	0.00095	0.00015	1.72	•	0.00092	0.00029	1.67	•
928.7461_7.59	TG(57:11)	0.00641	0.00183	0.00416	0.00123	0.00704	0.00355	1.69	•	0.01166	0.00207	2.80	•	0.01125	0.00165	2.70	•	0.01139	0.00424	2.74	•
931.6793_6.67	TG(57:12)	0.00138	0.00020	0.00161	0.00010	0.00164	0.00023	1.01		0.00188	0.00021	1.17		0.00182	0.00018	1.13		0.00148	0.00023	0.92	
960.8938_8.35	TG(58:2)	0.03358	0.00069	0.03344	0.00238	0.03720	0.00128	1.11		0.03632	0.00184	1.09		0.03667	0.00169	1.10		0.03398	0.00156	1.02	
960.8967_7.87	TG(58:2)	0.01239	0.00085	0.01216	0.00151	0.01319	0.00151	1.08		0.01426	0.00169	1.17		0.01581	0.00151	1.30		0.01292	0.00069	1.06	
958.8776_8.2	TG(58:3)	0.01273	0.00061	0.01237	0.00072	0.01387	0.00122	1.12		0.01445	0.00057	1.17		0.01508	0.00059	1.22		0.01587	0.00405	1.28	
958.8782_7.71	TG(58:3)	0.00783	0.00107	0.00675	0.00121	0.00874	0.00202	1.29		0.01049	0.00190	1.55	•	0.01038	0.00094	1.54	•	0.00915	0.00120	1.35	
956.8618_8.06	TG(58:4)	0.00476	0.00054	0.00458	0.00068	0.00617	0.00115	1.35		0.00758	0.00081	1.65	•	0.00781	0.00163	1.70	•	0.00912	0.00480	1.99	•
954.8420_7.41	TG(58:5)	0.00157	0.00034	0.00089	0.00045	0.00196	0.00101	2.20	•	0.00350	0.00072	3.93	•	0.00337	0.00062	3.79	•	0.00229	0.00052	2.57	•
954.8437_7.89	TG(58:5)	0.00499	0.00062	0.00485	0.00080	0.00653	0.00151	1.35		0.00833	0.00113	1.72	•	0.00845	0.00172	1.74	•	0.00830	0.00254	1.71	•
950.8127_7.61	TG(58:7)	0.01407	0.00363	0.01011	0.00258	0.01842	0.00825	1.82	•	0.02978	0.00582	2.94	•	0.02723	0.00373	2.69	•	0.03124	0.01588	3.09	•
950.9093_7.81	TG(58:7)	0.00049	0.00005	0.00066	0.00019	0.00067	0.00013	1.02		0.00073	0.00020	1.11		0.00075	0.00014	1.15		0.00054	0.00019	0.83	
931.7702_7.74	TG(58:8)	0.00600	0.00106	0.00516	0.00068	0.00726	0.00159	1.41		0.00990	0.00126	1.92	•	0.00936	0.00133	1.81	•	0.00919	0.00365	1.78	•
946.7835_7.45	TG(58:9)	0.03832	0.01524	0.02178	0.01414	0.05476	0.03940	2.51	•	0.09060	0.02042	4.16	•	0.08775	0.01825	4.03	•	0.09495	0.04721	4.36	•
944.7656_7.63	TG(58:10)	0.00029	0.00014	0.00022	0.00006	0.00038	0.00016	1.71		0.00063	0.00012	2.81	•	0.00062	0.00012	2.75	•	0.00198	0.00233	8.86	•
944.7687_7.28	TG(58:10)	0.02684	0.01100	0.01636	0.01225	0.03769	0.02918	2.30	•	0.05742	0.01451	3.51	•	0.05751	0.01226	3.52	•	0.05722	0.02196	3.50	•
972.8929_7.88	TG(59:3)	0.02837	0.00216	0.02621	0.00294	0.03076	0.00471	1.17		0.03359	0.00371	1.28		0.03682	0.00329	1.40		0.03013	0.00163	1.15	
970.8774_8.14	TG(59:4)	0.00127	0.00024	0.00107	0.00037	0.00186	0.00033	1.74	•	0.00191	0.00024	1.79	•	0.00189	0.00030	1.77	•	0.00286	0.00172	2.68	•
970.8778_7.73	TG(59:4)	0.01270	0.00156	0.01023	0.00156	0.01523	0.00389	1.49		0.01919	0.00370	1.88	•	0.01864	0.00221	1.82	•	0.01729	0.00508	1.69	•
968.8579_7.92	TG(59:5)	0.00197	0.00038	0.00158	0.00032	0.00200	0.00050	1.27		0.00248	0.00034	1.57	•	0.00248	0.00027	1.57	•	0.00382	0.00189	2.42	•
968.8627_7.56	TG(59:5)	0.01313	0.00256	0.01023	0.00234	0.01830	0.00616	1.79	•	0.02705	0.00614	2.64	•	0.02581	0.00337	2.52	•	0.01927	0.00370	1.88	•
966.8475_7.41	TG(59:6)	0.01118	0.00247	0.00861	0.00193	0.01516	0.00555	1.76	•	0.02371	0.00390	2.75	•	0.02297	0.00351	2.67	•	0.01549	0.00228	1.80	•
964.8353_7.25	TG(59:7)	0.00474	0.00073	0.00435	0.00054	0.00580	0.00186	1.33		0.00800	0.00095	1.84	•	0.00857	0.00115	1.97	•	0.00644	0.00083	1.48	
935.7183_7.04	TG(59:13)	0.00241	0.00029	0.00266	0.00029	0.00278	0.00037	1.05		0.00314	0.00034	1.18		0.00321	0.00027	1.21		0.00278	0.00018	1.05	
1000.9265_8.04	TG(60:1)	0.00279	0.00046	0.00264	0.00077	0.00268	0.00015	1.02		0.00363	0.00038	1.38		0.00373	0.00032	1.42		0.00295	0.00021	1.12	
986.9087_7.88	TG(60:3)	0.00693	0.00022	0.00634	0.00122	0.00771	0.00120	1.22		0.00807	0.00109	1.27		0.00895	0.00076	1.41		0.00778	0.00053	1.23	
984.8931_7.73	TG(60:4)	0.00333	0.00036	0.00236	0.00052	0.00390	0.00134	1.65	•	0.00475	0.00102	2.02	•	0.00470	0.00061	1.99	•	0.00402	0.00097	1.70	•
982.875_8.06	TG(60:5)	0.00325	0.00019	0.00348	0.00061	0.00424	0.00052	1.22		0.00480	0.00043	1.38		0.00489	0.00057	1.40		0.00463	0.00098	1.33	
982.8786_7.58	TG(60:5)	0.00276	0.00078	0.00214	0.00086	0.00423	0.00184	1.97	•	0.00628	0.00165	2.93	•	0.00581	0.00128	2.71	•	0.00413	0.00091	1.93	•
980.8636_7.41	TG(60:6)	0.00196	0.00061	0.00163	0.00033	0.00296	0.00133	1.82	•	0.00451	0.00088	2.77	•	0.00476	0.00090	2.92	•	0.00283	0.00040	1.74	•
978.8475_7.25	TG(60:7)	0.00026	0.00019	0.00025	0.00012	0.00054	0.00032	2.14	•	0.00105	0.00029	4.16	•	0.00085	0.00035	3.38	•	0.00050	0.00012	1.96	•
976.8321_7.78	TG(60:8)	0.00226	0.00034	0.00185	0.00054	0.00312	0.00150	1.68	•	0.00507	0.00083	2.74	•	0.00449	0.00097	2.42	•	0.00504	0.00269	2.72	•
974.8151_7.61	TG(60:9)	0.00383	0.00140	0.00256	0.00157	0.00600	0.00442	2.35	•	0.01044	0.00300	4.08	•	0.00980	0.00235	3.83	•	0.00965	0.00475	3.78	•
998.907_7.91	TG(61:4)	0.00032	0.00034	0.00014	0.00008	0.00041	0.00041	3.04	•	0.00126	0.00028	9.30	•	0.00136	0.00056	9.98	•	0.00106	0.00096	7.81	•
992.8649_7.59	TG(61:7)	0.00089	0.00044	0.00040	0.00035	0.00132	0.00135	3.34	•	0.00253	0.00103	6.40	•	0.00245	0.00053	6.19	•	0.00326	0.00169	8.24	•
988.8400_7.20	TG(61:9)	0.00100	0.00044	0.00062	0.00034	0.00110	0.00082	1.76	•	0.00179	0.00035	2.86		0.00182	0.00036	2.93	•	0.00149	0.00046	2.39	•
1004.8645_7.43	TG(62:8)	0.00017	0.00018	0.00010	0.00010	0.00029	0.00033	2.89	•	0.00064	0.00027	6.36	•	0.00061	0.00025	6.03	•	0.00075	0.00026	7.36	•
953.7559_7.61	TG(60:11)	0.00286	0.00118	0.00194	0.00089	0.00467	0.00305	2.40	•	0.00843	0.00186	4.35	•	0.00737	0.00157	3.80	•	0.00858	0.00353	4.42	•
970.7852_7.38	TG(60:11)	0.00755	0.00388	0.00368	0.00318	0.00955	0.00990	2.60	•	0.01617	0.00376	4.40	•	0.01566	0.00441	4.26	•	0.02186	0.01252	5.94	•
937.7624_7.4	TG(60:12)	0.00091	0.00026	0.00102	0.00028	0.00155	0.00024	1.52	•	0.00200	0.00046	1.96	•	0.00193	0.00028	1.89	•	0.00177	0.00049	1.73	•
968.7694_7.22	TG(60:12)	0.02450	0.01197	0.01395	0.01192	0.02326	0.02408	1.67	•	0.03441	0.00969	2.47	•	0.03611	0.01057	2.59	•	0.05069	0.02041	3.63	•
994.7850_7.31	TG(62:13)	0.00536	0.00371	0.00277	0.00324	0.00669	0.00915	2.41	•	0.01235	0.00414	4.46	•	0.01079	0.00531	3.89	•	0.02333	0.01606	8.42	•
992.7691_7.13	TG(62:14)	0.01273	0.00654	0.00739	0.00661	0.01353	0.01418	1.83	•	0.01877	0.00767	2.54	•	0.01854	0.00772	2.51	•	0.03283	0.01547	4.44	•
1016.8628_7.45	TG(63:9)	0.00040	0.00026	0.00024	0.00025	0.00089	0.00091	3.67	•	0.00177	0.00054	7.28	•	0.00145	0.00046	5.99	•	0.00157	0.00075	6.47	•
1014.8462_7.28	TG(63:10)	0.00030	0.00026	0.00020	0.00012	0.00097	0.00125	4.93	•	0.00181	0.00057	9.20	•	0.00183	0.00078	9.27	•	0.00187	0.00080	9.50	•

Supplementary Table 4: Triacylglyceride levels Day 1 with IR fold changes compared to control

m/z_Ret time	ID	C_D1 Average	0_D1 Average	Fold change to Control	5_D1 Average	Fold change to Control	15_D1 Average	Fold change to Control	25_D1 Average	Fold change to Control	83_D1 Average	Fold change to Control
684.6134_6.79	TG(38:0)	0.03915	0.04067	1.04	0.04148	1.06	0.04438	1.13	0.03940	1.01	0.04035	1.03
712.6447_7.04	TG(40:0)	0.04016	0.04050	1.01	0.03802	0.95	0.04154	1.03	0.03749	0.93	0.03940	0.98
740.6753_7.27	TG(42:0)	0.04153	0.04465	1.08	0.04557	1.10	0.04869	1.17	0.04350	1.05	0.04602	1.11
738.6602_7.07	TG(42:1)	0.02113	0.02152	1.02	0.02175	1.03	0.02340	1.11	0.01998	0.95	0.02245	1.06
752.6753_7.18	TG(43:1)	0.01786	0.01820	1.02	0.01799	1.01	0.01957	1.10	0.01755	0.98	0.01832	1.03
768.7061_7.48	TG(44:0)	0.15072	0.15449	1.03	0.15088	1.00	0.16118	1.07	0.14610	0.97	0.15180	1.01
754.6905_7.36	TG(44:1)	0.02914	0.02980	1.02	0.02878	0.99	0.03152	1.08	0.02900	1.00	0.02870	0.98
766.6907_7.3	TG(44:1)	0.05195	0.05497	1.06	0.05399	1.04	0.05813	1.12	0.05262	1.01	0.05463	1.05
764.6761_7.12	TG(44:2)	0.01530	0.01647	1.08	0.01705	1.11	0.01804	1.18	0.01601	1.05	0.01709	1.12
762.6620_6.93	TG(44:3)	0.00184	0.00192	1.04	0.00200	1.09	0.00207	1.12	0.00190	1.03	0.00199	1.08
782.7216_7.56	TG(45:0)	0.13016	0.13172	1.01	0.12678	0.97	0.13379	1.03	0.12097	0.93	0.13021	1.00
780.7066_7.4	TG(45:1)	0.07367	0.07426	1.01	0.07166	0.97	0.07812	1.06	0.07076	0.96	0.07397	1.00
778.6909_7.23	TG(45:2)	0.01686	0.01762	1.05	0.01690	1.00	0.01818	1.08	0.01640	0.97	0.01755	1.04
796.7375_7.67	TG(46:0)	0.21243	0.21594	1.02	0.21183	1.00	0.22412	1.06	0.20468	0.96	0.21439	1.01
794.7222_7.51	TG(46:1)	0.26979	0.27090	1.00	0.26391	0.98	0.28578	1.06	0.26145	0.97	0.26397	0.98
792.7064_7.34	TG(46:2)	0.06729	0.06784	1.01	0.06610	0.98	0.07186	1.07	0.06631	0.99	0.06724	1.00
790.691_7.18	TG(46:3)	0.00913	0.00963	1.06	0.00953	1.04	0.01040	1.14	0.00931	1.02	0.00955	1.05
808.7380_7.59	TG(47:1)	0.29083	0.29868	1.03	0.29537	1.02	0.31307	1.08	0.28163	0.97	0.30568	1.05
818.7224_7.38	TG(47:1)	0.02350	0.02465	1.05	0.02323	0.99	0.02500	1.06	0.02395	1.02	0.02376	1.01
806.7227_7.43	TG(47:2)	0.06470	0.06703	1.04	0.06649	1.03	0.07193	1.11	0.06540	1.01	0.06795	1.05
804.7066_7.28	TG(47:3)	0.01071	0.01157	1.08	0.01177	1.10	0.01241	1.16	0.01111	1.04	0.01192	1.11
824.6723_7.85	TG(48:0)	0.00945	0.00887	0.94	0.00960	1.02	0.00999	1.06	0.00909	0.96	0.00901	0.95
822.7534_7.7	TG(48:1)	0.40609	0.40607	1.00	0.39300	0.97	0.42209	1.04	0.39237	0.97	0.39612	0.98
832.738_7.48	TG(48:1)	0.02542	0.02664	1.05	0.02633	1.04	0.02845	1.12	0.02638	1.04	0.02693	1.06
820.7382_7.55	TG(48:2)	0.17631	0.17962	1.02	0.17169	0.97	0.17973	1.02	0.17008	0.96	0.17523	0.99
816.706_7.16	TG(48:4)	0.00548	0.00591	1.08	0.00613	1.12	0.00631	1.15	0.00565	1.03	0.00600	1.09
838.7821_7.49	TG(49:0)	0.02601	0.02640	1.01	0.02881	1.11	0.03171	1.22	0.02743	1.05	0.02815	1.08
838.7821_7.92	TG(49:0)	0.15071	0.15133	1.00	0.15161	1.01	0.16024	1.06	0.14147	0.94	0.15771	1.05
813.6944_6.67	TG(49:4)	0.00241	0.00250	1.04	0.00257	1.06	0.00309	1.28	0.00261	1.08	0.00254	1.05
830.7187_7.3	TG(49:4)	0.00233	0.00264	1.13	0.00258	1.11	0.00269	1.16	0.00257	1.11	0.00260	1.12
822.6553_7.72	TG(49:8)	0.00165	0.00210	1.28	0.00193	1.17	0.00222	1.35	0.00178	1.08	0.00207	1.26
852.7989_8.02	TG(50:0)	0.13522	0.14175	1.05	0.13878	1.03	0.14741	1.09	0.13466	1.00	0.14711	1.09
850.785_7.87	TG(50:1)	0.48810	0.48373	0.99	0.46696	0.96	0.50571	1.04	0.49131	1.01	0.45983	0.94
848.7694_7.71	TG(50:2)	0.36508	0.37116	1.02	0.35068	0.96	0.37194	1.02	0.40896	1.12	0.35839	0.98
848.7698_7.22	TG(50:2)	0.00088	0.00069	0.78	0.00082	0.93	0.00080	0.91	0.00084	0.96	0.00060	0.68 •
846.7536_7.56	TG(50:3)	0.09831	0.10163	1.03	0.09644	0.98	0.10118	1.03	0.11234	1.14	0.09916	1.01
844.7376_7.37	TG(50:4)	0.01477	0.01515	1.03	0.01473	1.00	0.01600	1.08	0.01853	1.25	0.01440	0.98
825.6935_7.53	TG(50:5)	0.02862	0.02796	0.98	0.02795	0.98	0.03082	1.08	0.02737	0.96	0.02702	0.94
842.7155_7.20	TG(50:5)	0.01087	0.01151	1.06	0.01179	1.08	0.01249	1.15	0.01227	1.13	0.01174	1.08
823.6781_7.37	TG(50:6)	0.01024	0.00975	0.95	0.00962	0.94	0.01088	1.06	0.00971	0.95	0.00945	0.92
862.7845_7.79	TG(51:2)	0.23470	0.24128	1.03	0.24627	1.05	0.26186	1.12	0.23864	1.02	0.25267	1.08
860.7690_7.66	TG(51:3)	0.03810	0.03955	1.04	0.03851	1.01	0.04122	1.08	0.04139	1.09	0.03943	1.03
865.72_7.64	TG(51:3)	0.00973	0.01014	1.04	0.01032	1.06	0.01123	1.15	0.01073	1.10	0.01024	1.05
858.7526_7.48	TG(51:4)	0.00971	0.01016	1.05	0.01030	1.06	0.01116	1.15	0.01145	1.18	0.01024	1.05
863.7064_7.46	TG(51:4)	0.00338	0.00346	1.02	0.00360	1.07	0.00400	1.18	0.00375	1.11	0.00361	1.07
861.6860_7.31	TG(51:5)	0.00398	0.00421	1.06	0.00518	1.30	0.00514	1.29	0.00481	1.21	0.00454	1.14
880.8308_8.17	TG(52:0)	0.07877	0.08697	1.10	0.08966	1.14	0.09075	1.15	0.08027	1.02	0.09308	1.18
878.8152_8.03	TG(52:1)	0.16286	0.16498	1.01	0.16202	0.99	0.17338	1.06	0.17470	1.07	0.16446	1.01
876.8008_7.87	TG(52:2)	0.45744	0.46770	1.02	0.43447	0.95	0.45975	1.01	0.54875	1.20	0.43167	0.94
874.7852_7.71	TG(52:3)	0.41404	0.44016	1.06	0.37583	0.91	0.37885	0.92	0.58758	1.42	0.38312	0.93
872.7695_7.56	TG(52:4)	0.34355	0.36568	1.06	0.33754	0.98	0.31631	0.92	0.42195	1.23	0.34402	1.00
853.7274_6.77	TG(52:5)	0.00420	0.00526	1.25	0.00537	1.28	0.00555	1.32	0.00542	1.29	0.00490	1.17
870.7506_7.41	TG(52:5)	0.09186	0.09091	0.99	0.09196	1.00	0.09484	1.03	0.10809	1.18	0.08994	0.98
868.7359_7.23	TG(52:6)	0.02689	0.02810	1.04	0.02856	1.06	0.03008	1.12	0.03097	1.15	0.02907	1.08
849.6938_7.37	TG(52:7)	0.00733	0.00729	0.99	0.00703	0.96	0.00813	1.11	0.00880	1.20	0.00683	0.93
866.7223_7.13	TG(52:7)	0.00200	0.00245	1.22	0.00219	1.09	0.00210	1.05	0.00233	1.16	0.00233	1.16
866.7232_7.07	TG(52:7)	0.00259	0.00286	1.10	0.00307	1.18	0.00342	1.32	0.00328	1.26	0.00300	1.16
871.6738_7.13	TG(52:7)	0.00258	0.00265	1.03	0.00274	1.06	0.00315	1.22	0.00291	1.13	0.00295	1.14
894.7528_7.25	TG(52:7)	0.12764	0.12868	1.01	0.13327	1.04	0.14182	1.11	0.15711	1.23	0.13469	1.06
864.7126_6.92	TG(52:8)	0.00304	0.00344	1.13	0.00360	1.18	0.00377	1.24	0.00344	1.13	0.00350	1.15
890.8148_7.96	TG(53:2)	0.10277	0.10323	1.00	0.10727	1.04	0.11157	1.09	0.10158	0.99	0.10884	1.06
888.7989_7.37	TG(53:3)	0.00319	0.00332	1.04	0.00335	1.05	0.00369	1.16	0.00327	1.02	0.00341	1.07
888.7991_7.81	TG(53:3)	0.02882	0.03066	1.06	0.03120	1.08	0.03292	1.14	0.03346	1.16	0.03164	1.10
886.7846_7.66	TG(53:4)	0.01260	0.01295	1.03	0.01257	1.00	0.01273	1.01	0.01484	1.18	0.01237	0.98
882.7614_6.92	TG(53:6)	0.00319	0.00375	1.18	0.00405	1.27	0.00381	1.19	0.00393	1.23	0.00381	1.19
876.7090_7.88	TG(53:9)	0.00185	0.00179	0.96	0.00180	0.97	0.00175	0.94	0.00281	1.51	• 0.00149	0.81
906.8469_8.18	TG(54:1)	0.11034	0.11495	1.04	0.11907	1.08	0.12490	1.13	0.11343	1.03	0.11818	1.07
904.8307_8.03	TG(54:2)	0.13056	0.13197	1.01	0.13070	1.00	0.13810	1.06	0.14680	1.12	0.13080	1.00
902.8163_7.88	TG(54:3)	0.36768	0.37088	1.01	0.34766	0.95	0.36850	1.00	0.40924	1.11	0.35033	0.95
902.8238_7.46	TG(54:3)	0.01154	0.01148	0.99	0.01231	1.07	0.01305	1.13	0.01173	1.02	0.01223	1.06
900.8009_7.73	TG(54:4)	0.29218	0.31557	1.08	0.28152	0.96	0.27926	0.96	0.38019	1.30	0.28824	0.99
898.7852_7.56	TG(54:5)	0.38859	0.43150	1.11	0.42250	1.09	0.40896	1.05	0.52053	1.34	0.42268	1.09
879.7443_6.71	TG(54:6)	0.01113	0.01399	1.26	0.01418	1.27	0.01459	1.31	0.01379	1.24	0.01383	1.24
896.7684_7.41	TG(54:6)	0.39531	0.40816	1.03	0.41384	1.05	0.41864	1.06	0.46895	1.19	0.41125	1.04
877.7298_6.56	TG(54:7)	0.00384	0.00405	1.06	0.00408	1.06	0.00425	1.11	0.00397	1.04	0.00388	1.01
892.7406_7.08	TG(54:8)	0.02847	0.02831	0.99	0.02967	1.04	0.03114	1.09	0.03153	1.11	0.02987	1.05
920.8618_7.87	TG(55:1)	0.06260	0.05990	0.96	0.05743	0.92	0.06895	1.10	0.05782	0.92	0.05398	0.86
918.8466_8.12	TG(55:2)	0.02849	0.02827	0.99	0.02989	1.05	0.03094	1.09	0.02947	1.03	0.02950	1.04
908.7723_7.43	TG(55:5)	0.00369	0.00437	1.18	0.00419	1.13	0.00396	1.07	0.00478	1.29	0.00471	1.27
910.782_7.59	TG(55:6)	0.00615	0.00696	1.13	0.00671	1.09	0.00668	1.09	0.00749	1.22	0.00751	1.22
891.7352_7.64	TG(55:7)	0.00327	0.00332	1.02	0.00343	1.05	0.00365	1.12	0.00395	1.21	0.00353	1.08
889.7236_7.48	TG(55:8)	0.00182	0.00170	0.93	0.00181	0.99	0.00207	1.13	0.00200	1.10	0.00167	0.92
906.7594_7.33	TG(55:8)	0.00270	0.00345	1.27	0.00336	1.24	0.00303	1.12	0.00293	1.08	0.00335	1.24
887.7038_7.10	TG(55:9)	0.00093	0.00144	1.55	• 0.00155	1.67	• 0.00158	1.70	• 0.00135	1.45	0.00175	1.88
887.7120_7.33	TG(55:9)	0.00364	0.00386	1.06	0.00385	1.06	0.00438	1.21	0.00384	1.06	0.00388	1.07
934.8778_8.32	TG(56:0)	0.11408	0.11734	1.03	0.12050	1.06	0.12644	1.11	0.11271	0.99	0.12273	1.08
932.8621_8.2	TG(56:2)	0.04394	0.04452	1.01	0.04505	1.03	0.04712	1.07	0.04655	1.06	0.04395	1.00
913.8172_8.32	TG(56:3)	0.00434	0.00580	1.34	0.00538	1.24	0.00663	1.53	• 0.00559	1.29	0.00565	1.30
928.8301_7.89	TG(56:4)	0.02626	0.02756	1.05	0.02614	1.00	0.02569	0.98	0.04149	1.58	• 0.02617	1.00
926.8112_7.48	TG(56:5)	0.00285	0.00287	1.01	0.00291	1.02	0.00334	1.17	0.00272	0.95	0.00303	1.06
907.7817_6.65	TG(56:6)	0.00256	0.00282	1.10	0.00259	1.01	0.00305	1.19	0.00247	0.97	0.00280	1.10

924.8018_7.67	TG(56:6)	0.05953	0.06668	1.12	0.06022	1.01	0.05303	0.89	0.10034	1.69	• 0.06236	1.05	
922.7852_7.59	TG(56:7)	0.03003	0.03691	1.23	0.02650	0.88	0.01832	0.61	• 0.04799	1.60	• 0.03341	1.11	
920.7695_7.43	TG(56:8)	0.14125	0.15568	1.10	0.15011	1.06	0.13073	0.93	0.18027	1.28	0.16080	1.14	
918.7549_7.20	TG(56:9)	0.03209	0.03745	1.17	0.03247	1.01	0.02822	0.88	0.03561	1.11	0.03834	1.19	
916.7409_7.05	TG(56:10)	0.01224	0.01319	1.08	0.01288	1.05	0.01204	0.98	0.01292	1.05	0.01380	1.13	
946.8773_7.87	TG(57:2)	0.05062	0.04781	0.94	0.04544	0.90	0.05340	1.06	0.05144	1.02	0.04180	0.83	
944.8624_8.14	TG(57:3)	0.00902	0.00922	1.02	0.00980	1.09	0.01027	1.14	0.01256	1.39	0.00976	1.08	
944.8632_7.71	TG(57:3)	0.02204	0.02271	1.03	0.01852	0.84	0.02222	1.01	0.02588	1.17	0.01882	0.85	
942.8471_7.56	TG(57:4)	0.02166	0.02241	1.03	0.01865	0.86	0.02042	0.94	0.02345	1.08	0.01992	0.92	
940.8291_7.41	TG(57:5)	0.00664	0.00676	1.02	0.00679	1.02	0.00695	1.05	0.00753	1.13	0.00676	1.02	
938.8148_7.76	TG(57:6)	0.00054	0.00067	1.24	0.00080	1.48	0.00061	1.13	0.00080	1.49	0.00076	1.41	
936.8054_7.67	TG(57:7)	0.00174	0.00210	1.20	0.00172	0.99	0.00217	1.25	0.00234	1.34	0.00208	1.19	
934.7865_7.52	TG(57:8)	0.00466	0.00572	1.23	0.00583	1.25	0.00560	1.20	0.00639	1.37	0.00607	1.30	
932.7702_7.27	TG(57:9)	0.00436	0.00519	1.19	0.00546	1.25	0.00551	1.26	0.00562	1.29	0.00584	1.34	
913.7242_7.41	TG(57:10)	0.00078	0.00082	1.05	0.00072	0.91	0.00084	1.07	0.00084	1.07	0.00091	1.16	
928.7461_7.59	TG(57:11)	0.00937	0.00999	1.07	0.00940	1.00	0.00818	0.87	0.01217	1.30	0.01068	1.14	
931.6793_6.67	TG(57:12)	0.00146	0.00165	1.13	0.00186	1.28	0.00188	1.29	0.00161	1.11	0.00166	1.14	
960.8938_8.35	TG(58:2)	0.03454	0.03475	1.01	0.03642	1.05	0.03855	1.12	0.03633	1.05	0.03576	1.04	
960.8967_7.87	TG(58:2)	0.01315	0.01280	0.97	0.01201	0.91	0.01399	1.06	0.01359	1.03	0.01093	0.83	
958.8776_8.2	TG(58:3)	0.01400	0.01399	1.00	0.01414	1.01	0.01479	1.06	0.01779	1.27	0.01427	1.02	
958.8782_7.71	TG(58:3)	0.00845	0.00876	1.04	0.00751	0.89	0.00927	1.10	0.00950	1.12	0.00737	0.87	
956.8618_8.06	TG(58:4)	0.00637	0.00733	1.15	0.00664	1.04	0.00652	1.02	0.01068	1.68	• 0.00631	0.99	
954.8420_7.41	TG(58:5)	0.00267	0.00254	0.95	0.00270	1.01	0.00277	1.04	0.00302	1.13	0.00249	0.93	
954.8437_7.89	TG(58:5)	0.00688	0.00806	1.17	0.00775	1.13	0.00722	1.05	0.00958	1.39	0.00738	1.07	
950.8127_7.61	TG(58:7)	0.02258	0.02670	1.18	0.02374	1.05	0.02145	0.95	0.03383	1.50	• 0.02518	1.11	
950.9093_7.81	TG(58:7)	0.00061	0.00065	1.05	0.00079	1.29	0.00083	1.35	0.00071	1.16	0.00071	1.16	
931.7702_7.74	TG(58:8)	0.00852	0.00857	1.01	0.00836	0.98	0.00809	0.95	0.01126	1.32	0.00836	0.98	
946.7835_7.45	TG(58:9)	0.06883	0.08291	1.20	0.08127	1.18	0.06825	0.99	0.10170	1.48	0.08746	1.27	
944.7656_7.63	TG(58:10)	0.00044	0.00055	1.24	0.00049	1.12	0.00048	1.09	0.00101	2.27	• 0.00059	1.33	
944.7687_7.28	TG(58:10)	0.04119	0.05296	1.29	0.05473	1.33	0.04272	1.04	0.05782	1.40	0.06080	1.48	
972.8929_7.88	TG(59:3)	0.03136	0.03068	0.98	0.02884	0.92	0.03251	1.04	0.03194	1.02	0.02573	0.82	
970.8774_8.14	TG(59:4)	0.00154	0.00175	1.13	0.00189	1.22	0.00180	1.17	0.00288	1.87	• 0.00184	1.20	
970.8778_7.73	TG(59:4)	0.01530	0.01633	1.07	0.01364	0.89	0.01569	1.03	0.01802	1.18	0.01362	0.89	
968.8579_7.92	TG(59:5)	0.00198	0.00225	1.13	0.00225	1.14	0.00238	1.20	0.00342	1.72	• 0.00190	0.96	
968.8627_7.56	TG(59:5)	0.02122	0.02330	1.10	0.02012	0.95	0.02207	1.04	0.02352	1.11	0.02051	0.97	
966.8475_7.41	TG(59:6)	0.01806	0.01812	1.00	0.01790	0.99	0.01939	1.07	0.02021	1.12	0.01802	1.00	
964.8353_7.25	TG(59:7)	0.00697	0.00675	0.97	0.00738	1.06	0.00751	1.08	0.00758	1.09	0.00671	0.96	
935.7183_7.04	TG(59:13)	0.00266	0.00280	1.05	0.00305	1.15	0.00336	1.26	0.00298	1.12	0.00323	1.21	
1000.9265_8.04	TG(60:1)	0.00303	0.00322	1.06	0.00322	1.06	0.00398	1.31	0.00401	1.32	0.00300	0.99	
986.9087_7.88	TG(60:3)	0.00766	0.00771	1.01	0.00687	0.90	0.00802	1.05	0.00745	0.97	0.00609	0.79	
984.8931_7.73	TG(60:4)	0.00392	0.00404	1.03	0.00309	0.79	0.00389	0.99	0.00429	1.10	0.00318	0.81	
982.875_8.06	TG(60:5)	0.00385	0.00444	1.15	0.00424	1.10	0.00442	1.15	0.00506	1.31	0.00425	1.10	
982.8786_7.58	TG(60:5)	0.00471	0.00521	1.10	0.00437	0.93	0.00497	1.05	0.00514	1.09	0.00440	0.93	
980.8636_7.41	TG(60:6)	0.00341	0.00344	1.01	0.00347	1.02	0.00396	1.16	0.00376	1.10	0.00339	1.00	
978.8475_7.25	TG(60:7)	0.00097	0.00064	0.65	• 0.00081	0.84	0.00101	1.04	0.00087	0.89	0.00056	0.58	•
976.8321_7.78	TG(60:8)	0.00306	0.00494	1.61	• 0.00461	1.50	• 0.00380	1.24	0.00562	1.84	0.00415	1.36	
974.8151_7.61	TG(60:9)	0.00746	0.00963	1.29	0.00895	1.20	0.00705	0.94	0.01098	1.47	0.01053	1.41	
998.907_7.91	TG(61:4)	0.00070	0.00102	1.47	0.00078	1.12	0.00062	0.88	0.00148	2.12	• 0.00046	0.66	•
992.8649_7.59	TG(61:7)	0.00196	0.00215	1.10	0.00190	0.97	0.00141	0.72	0.00285	1.46	0.00182	0.93	
988.8400_7.20	TG(61:9)	0.00156	0.00172	1.11	0.00165	1.06	0.00136	0.88	0.00152	0.98	0.00159	1.02	
1004.8645_7.43	TG(62:8)	0.00050	0.00050	0.99	0.00041	0.82	0.00032	0.64	• 0.00056	1.13	0.00045	0.91	
953.7559_7.61	TG(60:11)	0.00584	0.00700	1.20	0.00642	1.10	0.00544	0.93	0.00784	1.34	0.00693	1.19	
970.7852_7.38	TG(60:11)	0.01247	0.01917	1.54	• 0.01703	1.37	0.01088	0.87	0.01819	1.46	0.02092	1.68	•
937.7624_7.4	TG(60:12)	0.00132	0.00141	1.07	0.00139	1.06	0.00152	1.15	0.00156	1.19	0.00159	1.21	
968.7694_7.22	TG(60:12)	0.03150	0.04544	1.44	0.04656	1.48	0.02792	0.89	0.03696	1.17	0.05465	1.74	•
994.7850_7.31	TG(62:13)	0.01057	0.01589	1.50	• 0.01477	1.40	0.00909	0.86	0.01445	1.37	0.01690	1.60	•
992.7691_7.13	TG(62:14)	0.01811	0.02265	1.25	0.02326	1.28	0.01586	0.88	0.01957	1.08	0.02613	1.44	
1016.8628_7.45	TG(63:9)	0.00112	0.00137	1.22	0.00150	1.33	0.00104	0.93	0.00167	1.48	0.00153	1.36	
1014.8462_7.28	TG(63:10)	0.00094	0.00142	1.51	• 0.00174	1.84	• 0.00122	1.29	0.00175	1.85	• 0.00165	1.75	•

Supplementary Table 5: Triacylglyceride levels Day 7 with IR fold changes compared to control

m/z_Ret time	ID	C_D7 Average	0_D7 Average	Fold change to Control	5_D7 Average	Fold change to Control	15_D7 Average	Fold change to Control	25_D7 Average	Fold change to Control	83_D7 Average	Fold change to Control	
684.6134_6.79	TG(38:0)	0.03853	0.04111	1.07	0.04170	1.08	0.04319	1.12	0.04563	1.18	0.03830	0.99	
712.6447_7.04	TG(40:0)	0.04094	0.03906	0.95	0.04159	1.02	0.04340	1.06	0.04694	1.15	0.03678	0.90	
740.6753_7.27	TG(42:0)	0.04177	0.04410	1.06	0.04489	1.07	0.04585	1.10	0.04874	1.17	0.04149	0.99	
738.6602_7.07	TG(42:1)	0.02129	0.01993	0.94	0.02097	0.99	0.02187	1.03	0.02468	1.16	0.01908	0.90	
752.6753_7.18	TG(43:1)	0.01793	0.01772	0.99	0.01822	1.02	0.01910	1.06	0.02065	1.15	0.01682	0.94	
768.7061_7.48	TG(44:0)	0.15049	0.14275	0.95	0.15265	1.01	0.15743	1.05	0.17193	1.14	0.14034	0.93	
754.6905_7.36	TG(44:1)	0.02953	0.02845	0.96	0.03004	1.02	0.03108	1.05	0.03413	1.16	0.02684	0.91	
766.6907_7.3	TG(44:1)	0.05233	0.05156	0.99	0.05400	1.03	0.05644	1.08	0.06084	1.16	0.04910	0.94	
764.6761_7.12	TG(44:2)	0.01583	0.01599	1.01	0.01642	1.04	0.01699	1.07	0.01816	1.15	0.01492	0.94	
762.6620_6.93	TG(44:3)	0.00178	0.00181	1.02	0.00192	1.08	0.00219	1.23	0.00227	1.28	0.00158	0.89	
782.7216_7.56	TG(45:0)	0.13021	0.11874	0.91	0.12929	0.99	0.13510	1.04	0.14429	1.11	0.11833	0.91	
780.7066_7.4	TG(45:1)	0.07416	0.06768	0.91	0.07275	0.98	0.07589	1.02	0.08320	1.12	0.06532	0.88	
778.6909_7.23	TG(45:2)	0.01712	0.01634	0.95	0.01700	0.99	0.01780	1.04	0.01965	1.15	0.01537	0.90	
796.7375_7.67	TG(46:0)	0.20941	0.19832	0.95	0.21625	1.03	0.22130	1.06	0.23627	1.13	0.19808	0.95	
794.7222_7.51	TG(46:1)	0.27050	0.25071	0.93	0.26683	0.99	0.27814	1.03	0.30512	1.13	0.24452	0.90	
792.7064_7.34	TG(46:2)	0.06688	0.06374	0.95	0.06921	1.03	0.07122	1.06	0.07723	1.15	0.06161	0.92	
790.691_7.18	TG(46:3)	0.00909	0.00920	1.01	0.00961	1.06	0.01025	1.13	0.01117	1.23	0.00881	0.97	
808.7380_7.59	TG(47:1)	0.29052	0.26714	0.92	0.28743	0.99	0.29927	1.03	0.32303	1.11	0.26471	0.91	
818.7224_7.38	TG(47:1)	0.02294	0.02064	0.90	0.02301	1.00	0.02592	1.13	0.02863	1.25	0.02114	0.92	
806.7227_7.43	TG(47:2)	0.06395	0.06122	0.96	0.06447	1.01	0.06783	1.06	0.07396	1.16	0.06030	0.94	
804.7066_7.28	TG(47:3)	0.01081	0.01067	0.99	0.01124	1.04	0.01159	1.07	0.01242	1.15	0.01017	0.94	
824.6723_7.85	TG(48:0)	0.00921	0.00926	1.01	0.01012	1.10	0.00982	1.07	0.00891	0.97	0.00900	0.98	
822.7534_7.7	TG(48:1)	0.40014	0.36952	0.92	0.40284	1.01	0.42127	1.05	0.45396	1.13	0.37160	0.93	
832.738_7.48	TG(48:1)	0.02492	0.02335	0.94	0.02552	1.02	0.02726	1.09	0.03034	1.22	0.02385	0.96	
820.7382_7.55	TG(48:2)	0.17211	0.15617	0.91	0.17498	1.02	0.18747	1.09	0.20034	1.16	0.16181	0.94	
816.706_7.16	TG(48:4)	0.00491	0.00495	1.01	0.00563	1.15	0.00673	1.37	0.00718	1.46	0.00523	1.07	
838.7821_7.49	TG(49:0)	0.02553	0.02515	0.99	0.02416	0.95	0.02691	1.05	0.02981	1.17	0.02293	0.90	
838.7821_7.92	TG(49:0)	0.15155	0.14100	0.93	0.15447	1.02	0.15458	1.02	0.16134	1.06	0.13813	0.91	
813.6944_6.67	TG(49:4)	0.00225	0.00239	1.07	0.00258	1.15	0.00278	1.24	0.00292	1.30	0.00238	1.06	
830.7187_7.3	TG(49:4)	0.00210	0.00204	0.97	0.00213	1.01	0.00270	1.29	0.00316	1.51	• 0.00233	1.11	
822.6553_7.72	TG(49:8)	0.00159	0.00171	1.08	0.00177	1.11	0.00213	1.34	0.00240	1.51	• 0.00175	1.10	
852.7989_8.02	TG(50:0)	0.13341	0.13434	1.01	0.14734	1.10	0.14368	1.08	0.14707	1.10	0.13486	1.01	
850.785_7.87	TG(50:1)	0.47981	0.44787	0.93	0.49646	1.03	0.52549	1.10	0.55157	1.15	0.46532	0.97	
848.7694_7.71	TG(50:2)	0.33039	0.29710	0.90	0.34987	1.06	0.41753	1.26	0.43115	1.30	0.36144	1.09	
848.7698_7.22	TG(50:2)	0.00064	0.00049	0.76	0.00073	1.13	0.00077	1.19	0.00101	1.57	• 0.00075	1.17	
846.7536_7.56	TG(50:3)	0.08570	0.07328	0.86	0.09061	1.06	0.12077	1.41	0.12456	1.45	0.09436	1.10	
844.7376_7.37	TG(50:4)	0.01037	0.00891	0.86	0.01354	1.31	0.02130	2.05	• 0.02146	2.07	• 0.01348	1.30	
825.6935_7.53	TG(50:5)	0.03023	0.02879	0.95	0.02916	0.96	0.02994	0.99	0.03246	1.07	0.02649	0.88	
842.7155_7.20	TG(50:5)	0.00890	0.00870	0.98	0.01070	1.20	0.01448	1.63	• 0.01502	1.69	• 0.00968	1.09	
823.6781_7.37	TG(50:6)	0.01026	0.00978	0.95	0.01009	0.98	0.01080	1.05	0.01168	1.14	0.00918	0.90	
862.7845_7.79	TG(51:2)	0.23136	0.22448	0.97	0.24098	1.04	0.24484	1.06	0.25542	1.10	0.22383	0.97	
860.7690_7.66	TG(51:3)	0.03526	0.03227	0.92	0.03720	1.05	0.04288	1.22	0.04554	1.29	0.03709	1.05	
865.72_7.64	TG(51:3)	0.00898	0.00867	0.97	0.00976	1.09	0.01110	1.24	0.01129	1.26	0.00931	1.04	
858.7526_7.48	TG(51:4)	0.00799	0.00721	0.90	0.00945	1.18	0.01247	1.56	• 0.01333	1.67	• 0.00932	1.17	
863.7064_7.46	TG(51:4)	0.00322	0.00275	0.85	0.00334	1.04	0.00418	1.30	0.00416	1.29	0.00317	0.98	
861.6860_7.31	TG(51:5)	0.00323	0.00382	1.18	0.00419	1.30	0.00527	1.63	• 0.00541	1.67	• 0.00405	1.25	
880.8308_8.17	TG(52:0)	0.07611	0.07883	1.04	0.08715	1.15	0.08456	1.11	0.08460	1.11	0.08314	1.09	
878.8152_8.03	TG(52:1)	0.15978	0.15441	0.97	0.17166	1.07	0.17561	1.10	0.18177	1.14	0.16744	1.05	
876.8008_7.87	TG(52:2)	0.42219	0.38380	0.91	0.44917	1.06	0.54162	1.28	0.54068	1.28	0.47644	1.13	
874.7852_7.71	TG(52:3)	0.31158	0.24197	0.78	0.36664	1.18	0.56190	1.80	• 0.54787	1.76	• 0.48658	1.56	•
872.7695_7.56	TG(52:4)	0.23050	0.15189	0.66	• 0.28848	1.25	0.48048	2.08	• 0.48197	2.09	• 0.33701	1.46	
853.7274_6.77	TG(52:5)	0.00340	0.00399	1.17	0.00415	1.22	0.00545	1.60	• 0.00632	1.86	• 0.00343	1.01	
870.7506_7.41	TG(52:5)	0.06314	0.04938	0.78	0.08025	1.27	0.12689	2.01	• 0.12772	2.02	• 0.07963	1.26	
868.7359_7.23	TG(52:6)	0.02116	0.01908	0.90	0.02613	1.23	0.03708	1.75	• 0.03757	1.78	• 0.02395	1.13	
849.6938_7.37	TG(52:7)	0.00530	0.00463	0.87	0.00726	1.37	0.01055	1.99	• 0.01007	1.90	• 0.00697	1.31	
866.7223_7.13	TG(52:7)	0.00149	0.00152	1.02	0.00210	1.41	0.00307	2.06	• 0.00321	2.15	• 0.00213	1.43	
866.7232_7.07	TG(52:7)	0.00184	0.00172	0.94	0.00252	1.37	0.00346	1.88	• 0.00359	1.95	• 0.00206	1.12	
871.6738_7.13	TG(52:7)	0.00198	0.00148	0.74	0.00238	1.20	0.00351	1.77	• 0.00341	1.72	• 0.00210	1.06	
894.7528_7.25	TG(52:7)	0.08993	0.07570	0.84	0.11036	1.23	0.16311	1.81	• 0.16308	1.81	• 0.11157	1.24	
864.7126_6.92	TG(52:8)	0.00243	0.00226	0.93	0.00273	1.12	0.00391	1.61	0.00395	1.62	• 0.00215	0.88	
890.8148_7.96	TG(53:2)	0.10151	0.09677	0.95	0.10581	1.04	0.10618	1.05	0.11121	1.10	0.09745	0.96	
888.7989_7.37	TG(53:3)	0.00322	0.00296	0.92	0.00320	0.99	0.00348	1.08	0.00397	1.23	0.00320	0.99	
888.7991_7.81	TG(53:3)	0.02685	0.02527	0.94	0.02941	1.10	0.03334	1.24	0.03416	1.27	0.02920	1.09	
886.7846_7.66	TG(53:4)	0.00985	0.00814	0.83	0.01159	1.18	0.01503	1.53	• 0.01583	1.61	• 0.01272	1.29	
882.7614_6.92	TG(53:6)	0.00295	0.00322	1.09	0.00347	1.18	0.00444	1.50	• 0.00461	1.56	• 0.00319	1.08	
876.7090_7.88	TG(53:9)	0.00168	0.00132	0.79	0.00188	1.12	0.00276	1.64	• 0.00233	1.39	0.00287	1.71	•
906.8469_8.18	TG(54:1)	0.10863	0.10909	1.00	0.11846	1.09	0.11719	1.08	0.11803	1.09	0.10944	1.01	
904.8307_8.03	TG(54:2)	0.12484	0.12041	0.96	0.13505	1.08	0.14220	1.14	0.14427	1.16	0.13773	1.10	
902.8163_7.88	TG(54:3)	0.34101	0.31487	0.92	0.36629	1.07	0.40460	1.19	0.41204	1.21	0.37332	1.09	
902.8238_7.46	TG(54:3)	0.01042	0.00964	0.93	0.00967	0.93	0.01152	1.11	0.01239	1.19	0.00949	0.91	
900.8009_7.73	TG(54:4)	0.20972	0.17458	0.83	0.26161	1.25	0.36832	1.76	• 0.36244	1.73	• 0.31609	1.51	•
898.7852_7.56	TG(54:5)	0.24918	0.18555	0.74	0.32798	1.32	0.52691	2.11	• 0.51938	2.08	• 0.38085	1.53	•
879.7443_6.71	TG(54:6)	0.00961	0.01079	1.12	0.01207	1.26	0.01448	1.51	• 0.01553	1.62	• 0.01065	1.11	
896.7684_7.41	TG(54:6)	0.26191	0.20428	0.78	0.33260	1.27	0.51775	1.98	• 0.52289	2.00	• 0.33109	1.26	
877.7298_6.56	TG(54:7)	0.00285	0.00279	0.98	0.00369	1.29	0.00435	1.53	• 0.00470	1.65	• 0.00281	0.99	
892.7406_7.08	TG(54:8)	0.02075	0.01777	0.86	0.02553	1.23	0.03690	1.78	• 0.03695	1.78	• 0.02441	1.18	
920.8618_7.87	TG(55:1)	0.06225	0.05906	0.95	0.06293	1.01	0.06351	1.02	0.07255	1.17	0.05590	0.90	
918.8466_8.12	TG(55:2)	0.02784	0.02700	0.97	0.02939	1.06	0.02960	1.06	0.03075	1.10	0.02860	1.03	
908.7723_7.43	TG(55:5)	0.00280	0.00211	0.75	0.00297	1.06	0.00499	1.78	• 0.00500	1.79	• 0.00465	1.66	•
910.782_7.59	TG(55:6)	0.00502	0.00467	0.93	0.00603	1.20	0.00776	1.55	• 0.00773	1.54	• 0.00647	1.29	
891.7352_7.64	TG(55:7)	0.00282	0.00282	1.00	0.00328	1.16	0.00384	1.36	0.00392	1.39	0.00350	1.24	
889.7236_7.48	TG(55:8)	0.00120	0.00114	0.95	0.00178	1.49	0.00213	1.78	• 0.00233	1.94	• 0.00152	1.26	
906.7594_7.33	TG(55:8)	0.00209	0.00196	0.94	0.00265	1.27	0.00385	1.84	• 0.00388	1.86	• 0.00299	1.43	
887.7038_7.10	TG(55:9)	0.00090	0.00120	1.33	0.00144	1.60	0.00184	2.03	• 0.00180	1.99	• 0.00121	1.34	
887.7120_7.33	TG(55:9)	0.00324	0.00308	0.95	0.00340	1.05	0.00413	1.27	0.00441	1.36	0.00335	1.03	
934.8778_8.32	TG(56:0)	0.11246	0.11218	1.00	0.12089	1.07	0.11955	1.06	0.12016	1.07	0.10874	0.97	
932.8621_8.2	TG(56:2)	0.04242	0.04117	0.97	0.04619	1.09	0.04706	1.11	0.04774	1.13	0.04496	1.06	
913.8172_8.32	TG(56:3)	0.00450	0.00547	1.21	0.00595	1.32	0.00552	1.23	0.00487	1.08	0.00659	1.46	
928.8301_7.89	TG(56:4)	0.01988	0.01829	0.92	0.02445	1.23	0.03139	1.58	• 0.02996	1.51	• 0.03583	1.80	•
926.8112_7.48	TG(56:5)	0.00245	0.00216	0.88	0.00258	1.05	0.00288	1.17	0.00300	1.22	0.00231	0.94	
907.7817_6.65	TG(56:6)	0.00224	0.00242	1.08	0.00240	1.07	0.00281	1.25	0.00317	1.42	0.00222	0.99	

924.8018_7.67	TG(56:6)	0.03957	0.03106	0.78		0.05100	1.29		0.08003	2.02	•	0.07546	1.91	•	0.09532	2.41	•
922.7852_7.59	TG(56:7)	0.01691	0.00864	0.51	•	0.02290	1.35		0.04237	2.51	•	0.04089	2.42	•	0.06156	3.64	•
920.7695_7.43	TG(56:8)	0.09936	0.05909	0.59		0.10799	1.09		0.16934	1.70	•	0.17606	1.77	•	0.17200	1.73	•
918.7549_7.20	TG(56:9)	0.02186	0.01405	0.64	•	0.02554	1.17		0.04136	1.89	•	0.04176	1.91	•	0.03649	1.67	•
916.7409_7.05	TG(56:10)	0.01077	0.00795	0.74		0.01036	0.96		0.01458	1.35		0.01515	1.41		0.01239	1.15	
946.8773_7.87	TG(57:2)	0.04742	0.04351	0.92		0.04862	1.03		0.05450	1.15		0.05968	1.26		0.04710	0.99	
944.8624_8.14	TG(57:3)	0.00867	0.00865	1.00		0.00944	1.09		0.00973	1.12		0.00998	1.15		0.01245	1.44	
944.8632_7.71	TG(57:3)	0.01957	0.01518	0.78		0.02194	1.12		0.02866	1.46		0.02731	1.40		0.02497	1.28	
942.8471_7.56	TG(57:4)	0.01510	0.01146	0.76		0.01906	1.26		0.02877	1.91	•	0.02762	1.83	•	0.02105	1.39	
940.8291_7.41	TG(57:5)	0.00445	0.00324	0.73		0.00533	1.20		0.00892	2.01	•	0.00913	2.05	•	0.00584	1.31	
938.8148_7.76	TG(57:6)	0.00047	0.00031	0.67	•	0.00054	1.17		0.00092	1.98	•	0.00091	1.95	•	0.00073	1.56	•
936.8054_7.67	TG(57:7)	0.00135	0.00108	0.80		0.00178	1.31		0.00231	1.71	•	0.00241	1.78	•	0.00247	1.83	•
934.7865_7.52	TG(57:8)	0.00370	0.00333	0.90		0.00449	1.21		0.00593	1.60	•	0.00607	1.64	•	0.00557	1.51	•
932.7702_7.27	TG(57:9)	0.00359	0.00389	1.08		0.00466	1.30		0.00572	1.59	•	0.00597	1.67	•	0.00508	1.42	
913.7242_7.41	TG(57:10)	0.00050	0.00055	1.09		0.00071	1.42		0.00106	2.11	•	0.00095	1.88	•	0.00092	1.83	•
928.7461_7.59	TG(57:11)	0.00641	0.00416	0.65	•	0.00704	1.10		0.01166	1.82	•	0.01125	1.76	•	0.01139	1.78	•
931.6793_6.67	TG(57:12)	0.00138	0.00161	1.17		0.00164	1.18		0.00188	1.36		0.00182	1.32		0.00148	1.07	
960.8938_8.35	TG(58:2)	0.03358	0.03344	1.00		0.03720	1.11		0.03632	1.08		0.03667	1.09		0.03398	1.01	
960.8967_7.87	TG(58:2)	0.01239	0.01216	0.98		0.01319	1.06		0.01426	1.15		0.01581	1.28		0.01292	1.04	
958.8776_8.2	TG(58:3)	0.01273	0.01237	0.97		0.01387	1.09		0.01445	1.13		0.01508	1.18		0.01587	1.25	
958.8782_7.71	TG(58:3)	0.00783	0.00675	0.86		0.00874	1.12		0.01049	1.34		0.01038	1.33		0.00915	1.17	
956.8618_8.06	TG(58:4)	0.00476	0.00458	0.96		0.00617	1.30		0.00758	1.59	•	0.00781	1.64	•	0.00912	1.92	•
954.8420_7.41	TG(58:5)	0.00157	0.00089	0.57	•	0.00196	1.25		0.00350	2.24	•	0.00337	2.15	•	0.00229	1.46	
954.8437_7.89	TG(58:5)	0.00499	0.00485	0.97		0.00653	1.31		0.00833	1.67	•	0.00845	1.69	•	0.00830	1.66	•
950.8127_7.61	TG(58:7)	0.01407	0.01011	0.72		0.01842	1.31		0.02978	2.12	•	0.02723	1.94	•	0.03124	2.22	•
950.9093_7.81	TG(58:7)	0.00049	0.00066	1.34		0.00067	1.36		0.00073	1.49		0.00075	1.54	•	0.00054	1.11	
931.7702_7.74	TG(58:8)	0.00600	0.00516	0.86		0.00726	1.21		0.00990	1.65	•	0.00936	1.56	•	0.00919	1.53	•
946.7835_7.45	TG(58:9)	0.03832	0.02178	0.57	•	0.05476	1.43		0.09060	2.36	•	0.08775	2.29	•	0.09495	2.48	•
944.7656_7.63	TG(58:10)	0.00029	0.00022	0.77		0.00038	1.31		0.00063	2.16	•	0.00062	2.11	•	0.00198	6.80	•
944.7687_7.28	TG(58:10)	0.02684	0.01636	0.61	•	0.03769	1.40		0.05742	2.14	•	0.05751	2.14	•	0.05722	2.13	•
972.8929_7.88	TG(59:3)	0.02837	0.02621	0.92		0.03076	1.08		0.03359	1.18		0.03682	1.30		0.03013	1.06	
970.8774_8.14	TG(59:4)	0.00127	0.00107	0.84		0.00186	1.46		0.00191	1.51	•	0.00189	1.49		0.00286	2.25	•
970.8778_7.73	TG(59:4)	0.01270	0.01023	0.81		0.01523	1.20		0.01919	1.51	•	0.01864	1.47		0.01729	1.36	
968.8579_7.92	TG(59:5)	0.00197	0.00158	0.80		0.00200	1.01		0.00248	1.26		0.00248	1.26		0.00382	1.94	•
968.8627_7.56	TG(59:5)	0.01313	0.01023	0.78		0.01830	1.39		0.02705	2.06		0.02581	1.97	•	0.01927	1.47	
966.8475_7.41	TG(59:6)	0.01118	0.00861	0.77		0.01516	1.36		0.02371	2.12	•	0.02297	2.05	•	0.01549	1.39	
964.8353_7.25	TG(59:7)	0.00474	0.00435	0.92		0.00580	1.22		0.00800	1.69	•	0.00857	1.81	•	0.00644	1.36	
935.7183_7.04	TG(59:13)	0.00241	0.00266	1.10		0.00278	1.16		0.00314	1.30		0.00321	1.33		0.00278	1.15	
1000.9265_8.04	TG(60:1)	0.00279	0.00264	0.95		0.00268	0.96		0.00363	1.30		0.00373	1.34		0.00295	1.06	
986.9087_7.88	TG(60:3)	0.00693	0.00634	0.92		0.00771	1.11		0.00807	1.16		0.00895	1.29		0.00778	1.12	
984.8931_7.73	TG(60:4)	0.00333	0.00236	0.71		0.00390	1.17		0.00475	1.42		0.00470	1.41		0.00402	1.21	
982.875_8.06	TG(60:5)	0.00325	0.00348	1.07		0.00424	1.30		0.00480	1.47		0.00489	1.50	•	0.00463	1.42	
982.8786_7.58	TG(60:5)	0.00276	0.00214	0.78		0.00423	1.53	•	0.00628	2.28	•	0.00581	2.11	•	0.00413	1.50	•
980.8636_7.41	TG(60:6)	0.00196	0.00163	0.83		0.00296	1.52	•	0.00451	2.31	•	0.00476	2.43	•	0.00283	1.45	
978.8475_7.25	TG(60:7)	0.00026	0.00025	0.99		0.00054	2.10	•	0.00105	4.10	•	0.00085	3.33	•	0.00050	1.93	•
976.8321_7.78	TG(60:8)	0.00226	0.00185	0.82		0.00312	1.38		0.00507	2.25	•	0.00449	1.99		0.00504	2.24	•
974.8151_7.61	TG(60:9)	0.00383	0.00256	0.67	•	0.00600	1.57	•	0.01044	2.73	•	0.00980	2.56	•	0.00965	2.52	•
998.907_7.91	TG(61:4)	0.00032	0.00014	0.43	•	0.00041	1.29		0.00126	3.95	•	0.00136	4.24	•	0.00106	3.32	•
992.8649_7.59	TG(61:7)	0.00089	0.00040	0.44	•	0.00132	1.48		0.00253	2.84	•	0.00245	2.74	•	0.00326	3.65	•
988.8400_7.20	TG(61:9)	0.00100	0.00062	0.62	•	0.00110	1.10		0.00179	1.79	•	0.00182	1.83	•	0.00149	1.49	
1004.8645_7.43	TG(62:8)	0.00017	0.00010	0.61	•	0.00029	1.75	•	0.00064	3.85	•	0.00061	3.65	•	0.00075	4.46	•
953.7559_7.61	TG(60:11)	0.00286	0.00194	0.68		0.00467	1.63	•	0.00843	2.95	•	0.00737	2.58	•	0.00858	3.00	•
970.7852_7.38	TG(60:11)	0.00755	0.00368	0.49	•	0.00955	1.27		0.01617	2.14	•	0.01566	2.07	•	0.02186	2.89	•
937.7624_7.4	TG(60:12)	0.00091	0.00102	1.12		0.00155	1.70	•	0.00200	2.19	•	0.00193	2.11	•	0.00177	1.94	•
968.7694_7.22	TG(60:12)	0.02450	0.01395	0.57	•	0.02326	0.95		0.03441	1.40		0.03611	1.47		0.05069	2.07	•
994.7850_7.31	TG(62:13)	0.00536	0.00277	0.52	•	0.00669	1.25		0.01235	2.30	•	0.01079	2.01	•	0.02333	4.35	•
992.7691_7.13	TG(62:14)	0.01273	0.00739	0.58	•	0.01353	1.06		0.01877	1.47		0.01854	1.46		0.03283	2.58	•
1016.8628_7.45	TG(63:9)	0.00040	0.00024	0.61	•	0.00089	2.25	•	0.00177	4.46	•	0.00145	3.67	•	0.00157	3.96	•
1014.8462_7.28	TG(63:10)	0.00030	0.00020	0.65	•	0.00097	3.19	•	0.00181	5.96	•	0.00183	6.01	•	0.00187	6.15	•

Supplementary Table 6: ESI+ mode lipids at Day 1 after IR with fold changes of neutron contribution compared to photons.

		C_D1		0_D1		5_D1		Fold change		15_D1		Fold change		25_D1		Fold change		83_D1		Fold change	
m/z_Ret time	ID	Average	SD	Average	SD	Average	SD	to X-ray (0%)		Average	SD	to X-ray (0%)		Average	SD	to X-ray (0%)		Average	SD	to X-ray (0%)	
666.6184_7.8	CE(18:2)	417580.61	51511.33	578318.43	87894.36	667761.12	171111.43	1.15		588924.87	119728.89	1.02		1186368.91	1396685.51	2.05	•	584241.36	178076.84	1.01	
690.6185_7.67	CE(20:4)	593469.68	132665.83	723371.02	80907.59	897870.62	300897.36	1.24		704063.21	210078.14	0.97		1798903.91	2465303.47	2.49	•	724667.61	208028.04	1.00	
671.5729_7.97	CE(20:5)	3766.19	2881.89	4107.61	2293.66	4496.47	1109.12	1.09		4089.26	2647.01	1.00		44189.15	88402.12	10.76	•	4718.04	2506.57	1.15	
688.6024_7.51	CE(20:5)	107699.38	29804.78	148245.70	51109.11	131654.44	46438.52	0.89		123603.66	36875.13	0.83		213726.43	192653.02	1.44		115866.16	30585.26	0.78	
719.5759_7.99	CE(22:6)	648.53	668.42	1046.09	658.04	1748.47	865.26	1.67		1861.91	861.74	1.78		12488.77	24545.53	11.94	•	1651.04	256.57	1.58	•
570.5478_4.71	Cer(d18:0/h17:0)	0.47632	0.00661	0.48555	0.12008	0.43755	0.02906	0.90		0.51359	0.01666	1.06		0.56172	0.01187	1.16		0.39282	0.04432	0.81	
570.5466_5.08	Cer(d18:0/h17:0)	0.00891	0.00083	0.00860	0.00058	0.01028	0.00110	1.20		0.00877	0.00078	1.02		0.00807	0.00033	0.94		0.00988	0.00080	1.15	
750.6474_5.56	SM(d18:1/18:0)	0.02923	0.00175	0.03734	0.00423	0.03600	0.00362	0.96		0.03396	0.00307	0.91		0.03088	0.00403	0.83		0.03797	0.00529	1.02	
759.6369_5.52	SM(d18:1/20:0)	0.01766	0.00310	0.02279	0.00471	0.01997	0.00313	0.88		0.01678	0.00293	0.74		0.01511	0.00222	0.66	•	0.01980	0.00413	0.87	
785.6524_5.59	SM(d18:1/22:1)	0.06827	0.00692	0.07930	0.01165	0.05624	0.01280	0.71		0.05891	0.00833	0.74		0.04907	0.00709	0.62	•	0.05937	0.01619	0.75	
752.6711_7.71	GlcCer(d18:2/20:1)	0.00415	0.00021	0.00414	0.00034	0.00421	0.00025	1.02		0.00464	0.00015	1.12		0.00422	0.00029	1.02		0.00446	0.00037	1.08	
605.5498_5.23	DG(34:0)	23427.47	15532.74	25344.34	19486.11	29385.98	14490.81	1.16		33344.98	6939.41	1.32		36857.98	2953.34	1.45		27525.91	3918.99	1.09	
610.5403_5.87	DG(34:2)	727875.15	38486.64	596816.61	119988.11	716532.39	92963.98	1.20		677802.49	74404.48	1.14		657609.87	28151.61	1.10		648172.93	29498.72	1.09	
633.543_6.62	DG(35:0)	51083.32	5562.29	49755.38	6657.20	57274.53	10022.85	1.15		50984.32	6011.37	1.02		51631.71	9744.30	1.04		55324.41	6050.05	1.11	
619.5268_6.38	DG(36:3)	541210.69	169162.78	285191.77	8659.72	339046.02	58208.89	1.19		308508.07	35199.78	1.08		292413.26	34324.25	1.03		314505.44	43992.30	1.10	
661.5736_6.92	DG(37:0)	0.00418	0.00020	0.00436	0.00058	0.00437	0.00031	1.00		0.00507	0.00068	1.16		0.00433	0.00053	0.99		0.00455	0.00097	1.04	
637.4824_5.36	DG(38:8)	26052.92	6075.73	20020.14	5529.41	26396.71	5612.55	1.32		21644.98	4134.40	1.08		24408.18	4039.79	1.22		23159.86	3669.09	1.16	
682.5424_5.33	DG(40:8)	103991.57	24432.22	69972.88	21709.71	78577.92	9394.79	1.12		66922.49	9545.78	0.96		56785.81	8387.36	0.81		77183.43	9965.19	1.10	
482.3241_1.06	LPC(15:0)	0.02386	0.00352	0.03042	0.00318	0.02465	0.00685	0.81		0.02726	0.00214	0.90		0.02561	0.00157	0.84		0.02657	0.00378	0.87	
482.3242_1.9	LPC(15:0)	0.01605	0.00248	0.01704	0.00330	0.01354	0.00334	0.79		0.01510	0.00180	0.89		0.01531	0.00097	0.90		0.01367	0.00278	0.80	
518.3242_0.9	LPC(18:3)	0.03215	0.00553	0.04543	0.00563	0.03773	0.01491	0.83		0.04752	0.00609	1.05		0.04804	0.00282	1.06		0.04298	0.00527	0.95	
550.3871_1.88	LPC(20:1)	0.03320	0.00547	0.04560	0.00599	0.03737	0.01188	0.82		0.04065	0.00451	0.89		0.03670	0.00470	0.80		0.03746	0.00762	0.82	
572.3694_1.88	LPC(22:4)	0.00380	0.00034	0.00556	0.00082	0.00508	0.00054	0.91		0.00547	0.00089	0.98		0.00464	0.00060	0.83		0.00500	0.00030	0.90	
709.5803_4.6	PC(30:0)	0.00183	0.00025	0.00204	0.00051	0.00185	0.00046	0.90		0.00193	0.00023	0.95		0.00199	0.00024	0.98		0.00196	0.00035	0.96	
720.5539_4.9	PC(31:0)	0.00231	0.00032	0.00237	0.00043	0.00248	0.00079	1.05		0.00288	0.00039	1.22		0.00320	0.00030	1.35		0.00280	0.00062	1.18	
716.5230_4.97	PC(31:2)	0.00779	0.00085	0.00810	0.00278	0.00635	0.00142	0.78		0.00691	0.00053	0.85		0.00690	0.00066	0.85		0.00620	0.00080	0.77	
716.562_4.70	PC(32:2)	0.00485	0.00074	0.00574	0.00102	0.00609	0.00198	1.06		0.00663	0.00048	1.15		0.00697	0.00044	1.21		0.00624	0.00072	1.09	
730.5381_4.36	PC(32:2)	0.00421	0.00089	0.00667	0.00225	0.00396	0.00176	0.59	•	0.00511	0.00054	0.77		0.00564	0.00031	0.84		0.00587	0.00135	0.88	
748.5831_5.33	PC(33:0)	0.00065	0.00008	0.00071	0.00022	0.00055	0.00021	0.77		0.00065	0.00008	0.92		0.00078	0.00011	1.10		0.00065	0.00015	0.91	
730.5758_4.9	PC(33:1)	0.00068	0.00014	0.00073	0.00038	0.00064	0.00018	0.89		0.00068	0.00014	0.94		0.00080	0.00009	1.11		0.00071	0.00022	0.98	
746.5689_4.96	PC(33:1)	0.00728	0.00089	0.00759	0.00241	0.00522	0.00159	0.69		0.00625	0.00064	0.82		0.00617	0.00051	0.81		0.00630	0.00109	0.83	
728.5605_5.62	PC(33:2)	0.00130	0.00021	0.00136	0.00042	0.00118	0.00026	0.87		0.00112	0.00012	0.82		0.00118	0.00007	0.86		0.00112	0.00028	0.82	
744.5558_5.41	PC(33:2)	0.01059	0.00179	0.01062	0.00226	0.00992	0.00338	0.93		0.00983	0.00126	0.93		0.01035	0.00105	0.98		0.00968	0.00201	0.91	
742.5373_4.36	PC(33:3)	0.00221	0.00029	0.00252	0.00018	0.00262	0.00045	1.04		0.00275	0.00021	1.09		0.00265	0.00015	1.05		0.00290	0.00037	1.15	
740.5237_4.69	PC(33:4)	0.00319	0.00090	0.00457	0.00108	0.00382	0.00162	0.84		0.00530	0.00093	1.16		0.00548	0.00084	1.20		0.00469	0.00110	1.03	
762.5997_5.55	PC(34:0)	0.00883	0.00102	0.01145	0.00117	0.01078	0.00408	0.94		0.01196	0.00250	1.04		0.01240	0.00150	1.08		0.01303	0.00288	1.14	
742.5691_5.06	PC(34:2)	0.00320	0.00077	0.00418	0.00125	0.00290	0.00157	0.69		0.00377	0.00066	0.90		0.00360	0.00055	0.86		0.00305	0.00091	0.73	
740.5593_4.6	PC(34:3)	0.00174	0.00028	0.00243	0.00022	0.00235	0.00072	0.97		0.00242	0.00062	1.00		0.00249	0.00042	1.03		0.00221	0.00049	0.91	
756.5543_4.49	PC(34:3)	0.07544	0.01576	0.10045	0.03756	0.05945	0.02979	0.59	•	0.07944	0.00734	0.79		0.08368	0.00720	0.83		0.08179	0.01719	0.81	
738.546_4.25	PC(34:4)	0.00088	0.00011	0.00105	0.00025	0.00093	0.00022	0.89		0.00093	0.00009	0.89		0.00095	0.00006	0.91		0.00094	0.00015	0.90	
754.5333_4.29	PC(34:4)	0.00313	0.00055	0.00428	0.00154	0.00277	0.00084	0.65	•	0.00342	0.00034	0.80		0.00370	0.00034	0.86		0.00382	0.00085	0.89	
772.5851_5.08	PC(35:2)	0.05168	0.01003	0.05856	0.00938	0.04719	0.01813	0.81		0.05827	0.00741	1.00		0.06062	0.00499	1.04		0.05709	0.01004	0.97	
770.5692_4.70	PC(35:3)	0.02050	0.00604	0.02625	0.00729	0.01965	0.00965	0.75		0.02513	0.00243	0.96		0.02540	0.00196	0.97		0.02376	0.00463	0.91	
754.5719_5.62	PC(35:4)	0.00507	0.00080	0.00537	0.00089	0.00579	0.00112	1.08		0.00527	0.00037	0.98		0.00532	0.00071	0.99		0.00564	0.00074	1.05	
768.5540_5.36	PC(35:4)	0.00947	0.00108	0.00975	0.00180	0.00833	0.00260	0.85		0.00711	0.00092	0.73		0.00750	0.00096	0.77		0.00901	0.00178	0.92	
766.5427_5.09	PC(35:5)	0.00152	0.00026	0.00148	0.00035	0.00125	0.00046	0.84		0.00122	0.00020	0.82		0.00129	0.00020	0.87		0.00145	0.00043	0.98	
764.5231_4.83	PC(35:6)	0.02714	0.00441	0.03144	0.01208	0.01672	0.00553	0.53	•	0.01661	0.00288	0.53	•	0.01624	0.00270	0.52	•	0.02357	0.00522	0.75	
786.6002_5.30	PC(36:2)	2.84450	0.55491	3.20032	0.90563	2.55958	1.18901	0.80		3.35955	0.40179	1.05		3.73882	0.33108	1.17		2.98701	0.70135	0.93	
766.57_4.99	PC(36:4)	0.01202	0.00088	0.01245	0.00312	0.01104	0.00276	0.89		0.01201	0.00158	0.96		0.01122	0.00127	0.90		0.01170	0.00194	0.94	
766.5719_4.78	PC(36:4)	0.00753	0.00131	0.01034	0.00237	0.00688	0.00305	0.67	•	0.00882	0.00052	0.85		0.00826	0.00065	0.80		0.00941	0.00125	0.91	
782.56939_4.57	PC(36:4)	0.16983	0.07166	0.27152	0.08326	0.17590	0.11852	0.65	•	0.28261	0.04541	1.04		0.34853	0.03328	1.28		0.26843	0.05172	0.99	
782.5694_4.82	PC(36:4)	1.67834	0.28089	2.35094	0.35270	1.34471	0.66824	0.57	•	1.70500	0.35236	0.73		1.97907	0.26050	0.84		2.14849	0.62533	0.91	
788.5521_4.99	PC(36:4)	0.00060	0.00010	0.00069	0.00039	0.00029	0.00015	0.43	•	0.00035	0.00013	0.52	•	0.00044	0.00011	0.64	•	0.00035	0.00009	0.52	•
780.5540_4.22	PC(36:5)	0.01384	0.00367	0.02439	0.00596	0.01285	0.00567	0.53	•	0.02035	0.00341	0.83		0.02356	0.00161	0.97		0.02238	0.00495	0.92	
781.5896_5.93	PC(36:5)	0.00805	0.00082	0.00777	0.00112	0.00776	0.00123	1.00		0.00739	0.00049	0.95		0.00781	0.00062	1.00		0.00787	0.00079	1.01	
778.5362_4.54	PC(36:6)	0.00280	0.00096	0.00422	0.00209	0.00199	0.00129	0.47	•	0.00295	0.00063	0.70		0.00352	0.00051	0.83		0.00293	0.00102	0.69	
798.60152_5.12	PC(37:3)	0.00887	0.00117	0.01059	0.00209	0.00805	0.00241	0.76		0.01070	0.00180	1.01		0.01095	0.00065	1.03		0.00974	0.00181	0.92	
813.6184_5.33	PC(37:4)	0.04112	0.00252	0.04938	0.01217	0.03936	0.01271	0.80		0.04337	0.00593	0.88		0.04505	0.00324	0.91		0.04573	0.00914	0.93	
794.5709_4.72	PC(37:5)	0.01161	0.00162	0.01450	0.00205	0.00974	0.00314	0.67	•	0.01193	0.00095	0.82		0.01242	0.00121	0.86		0.01394	0.00153	0.96	
790.5744_4.89	PC(38:6)	0.01703	0.00257	0.02321	0.00517	0.01599	0.00773	0.69		0.02106	0.00335	0.91		0.02130	0.00405	0.92		0.01982	0.00398	0.85	
806.5698_4.70	PC(38:6)	15.96005	2.44189	20.84641	2.12053	11.65760	3.70209	0.56	•	13.52152	2.67269	0.65	•	12.28556	2.62678	0.59	•	16.93467	2.95291	0.81	
804.552_4.57	PC(38:7)	0.03121	0.00464	0.04268	0.01112	0.02494	0.01093	0.58	•	0.03606	0.00506	0.85		0.04106	0.00383	0.96		0.03761	0.00765	0.88	
802.5358_4.49	PC(38:8)	0.00417	0.00090	0.00560	0.00158	0.00277	0.00173	0.50	•	0.00417	0.00055	0.74		0.00485	0.00093	0.87		0.00520	0.00185	0.93	
824.6168_5.45	PC(39:4)	0.00780	0.00072	0.00934	0.00119	0.00872	0.00158	0.93		0.00848	0.00140	0.91		0.00879	0.00098	0.94		0.01053	0.00201	1.13	
830.5662_4.97	PC(40:8)	0.01044	0.00114	0.01077	0.00183	0.00745	0.00113	0.69		0.00883	0.00076	0.82		0.00914	0.00136	0.85		0.01025	0.00230	0.95	
830.5694_4.39	PC(40:8)	0.02277	0.00171	0.03179	0.00300	0.02052	0.00665	0.65	•	0.02622	0.00411	0.82		0.02699	0.00350	0.85		0.02999	0.00628	0.94	
847.6036_4.55	PC(40:8)	0.00343	0.00011	0.00381	0.00020	0.00382	0.00048	1.00		0.00406	0.00045	1.06		0.00366	0.00033	0.96		0.00423	0.00039	1.11	
828.5589_4.1	PC(40:9)	0.00149	0.00014	0.00246	0.00025	0.00185	0.00031	0.75		0.00220	0.00036	0.89		0.00201	0.00033	0.82		0.00254	0.00066	1.03	
873.7503_7.15	PC(42:1)	0.00588	0.00080	0.00592	0.00054	0.00636	0.00196	1.07		0.00617	0.00053	1.04		0.00699	0.00198	1.18		0.00626	0.00182	1.06	
852.6819_6.16	PC(42:4)	0.00329	0.00014	0.00405	0.00031	0.00412	0.00062	1.02		0.00391	0.00035	0.97		0.00376	0.00026	0.93		0.00410	0.00045	1.01	
822.5994_4.67	PC(42:5)	0.00798	0.00158	0.00987	0.00185	0.00863	0.00357	0.87		0.00963	0.00141	0.98		0.00855	0.00133	0.87		0.00902	0.00163	0.91	
852.5592_4.46	PC(42:5)	0.00615	0.00115	0.00915	0.00205	0.00573	0.00252	0.63	•	0.00700	0.00121	0.77		0.00707	0.00070	0.77		0.00765	0.00138	0.84	
846.6366_5.72	PC(42:6)	0.00110	0.00007	0.00124	0.00016	0.00136	0.00041	1.10		0.00119	0.00018	0.96		0.00115	0.00030	0.92		0.00139	0.00029	1.12	
858.6002_4.83	PC(42:8)	0.00256	0.00035	0.00305	0.00058	0.00233	0.00065	0.76		0.00267	0.00030	0.88		0.00230	0.00057	0.75		0.00307	0.00079	1.01	
880.5906_4.91	PC(42:8)	0.00818	0.00233	0.00983	0.00439	0.00817	0.00411	0.83		0.01055	0.00144	1.07		0.01051	0.00205	1.07		0.00777	0.00217	0.79	
856.5836_4.47	PC(42:9)	0.00160	0.00016	0.00232	0.00036	0.00159	0.00049	0.68		0.00207	0.00032	0.89		0.00200	0.00022	0.86		0.00215	0.00048	0.92	
854.5698_4.31	PC(42:10)	0.00383	0.00050	0.00561	0.00050	0.00333	0.00100	0.59	•	0.00408	0.00081	0.73		0.00462	0.00061	0.82		0.00559	0.00128	1.00	
904.5912_4.88	PC(44:10)	0.04943	0.01071	0.06321	0.01285	0.06150	0.01911	0.97		0.06696	0.01219	1.06		0.06209	0.01215	0.98		0.07856	0.02082	1.24	
902.5751_4.6	PC(44:11)	0.00188	0.00034	0.00312	0.00068	0.00215	0.00103	0.69		0.00273	0.00043	0.88		0.00243	0.00029	0.78		0.00292	0.00091	0.93	
902.5758_4.47	PC(44:11)	0.00237	0.00057	0.00305	0.00027	0.00202	0.00065	0.66	•	0.00201	0.00039	0.66	•	0.00183	0.00026	0.60	•	0.00254	0.00099	0.83	
878.5758_4.53	PC(44:12)	0.00203	0.00046	0.00323	0.00049	0.00240	0.00102	0.74		0.00296	0.00045	0.92		0.00247	0.00036	0.77		0.00286	0.00088	0.89	
935.7295_6.47	PC(46:6)	0.00653	0.00050	0.00680	0.00100	0.00682	0.00119	1.00		0.00675	0.00063	0.99		0.00643	0.00024	0.95		0.00668	0.00121	0.98	

Supplementary Table 7: ESI+ mode lipids at Day 7 after IR with fold changes of neutron contribution compared to photons.

		C_D7		0_D7		5_D7		Fold change		15_D7		Fold change		25_D7		Fold change		83_D7		Fold change	
m/z_Ret time	ID	Average	SD	Average	SD	Average	SD	to X-ray (0%)		Average	SD	to X-ray (0%)		Average	SD	to X-ray (0%)		Average	SD	to X-ray (0%)	
666.6184_7.8	CE(18:2)	456944.68	120368.89	313267.04	153091.44	394573.61	165925.95	1.26		417823.43	102354.02	1.33		335622.36	42058.15	1.07		1602376.12	1206590.59	5.12	•
690.6185_7.67	CE(20:4)	730098.27	231380.10	379645.04	237957.02	509044.50	197789.87	1.34		588028.32	132528.55	1.55	•	511827.73	75169.80	1.35		2254112.54	1824578.04	5.94	•
671.5729_7.97	CE(20:5)	3307.64	1743.27	3612.24	1149.03	2957.22	1284.95	0.82		2920.58	1098.45	0.81		2261.53	858.42	0.63	•	72610.84	103506.48	20.10	•
688.6024_7.51	CE(20:5)	86301.08	30860.46	68627.73	57628.83	67923.10	39578.52	0.99		109161.74	33809.38	1.59	•	95116.24	9114.18	1.39		249946.79	114557.20	3.64	•
719.5759_7.99	CE(22:6)	1225.98	1076.28	1983.47	1008.73	1182.28	629.15	0.60		627.85	924.19	0.32	•	1063.63	1003.54	0.54	•	21599.29	35061.94	10.89	•
570.5478_4.71	Cer(d18:0/h17:0)	0.45991	0.01234	0.56835	0.09593	0.63719	0.01181	1.12		0.55752	0.01448	0.98		0.51392	0.01310	0.90		0.64600	0.00611	1.14	
570.5466_5.08	Cer(d18:0/h17:0)	0.00912	0.00045	0.01109	0.00302	0.00859	0.00059	0.77		0.00778	0.00070	0.70		0.00811	0.00050	0.73		0.00929	0.00051	0.84	
750.6474_5.56	SM(d18:1/18:0)	0.02927	0.00165	0.04063	0.01045	0.03309	0.00266	0.81		0.03215	0.00138	0.79		0.03245	0.00111	0.80		0.03358	0.00376	0.83	
759.6369_5.52	SM(d18:1/20:0)	0.02125	0.00400	0.01655	0.00750	0.01418	0.00105	0.86		0.01115	0.00215	0.67	•	0.01251	0.00310	0.76		0.01731	0.00207	1.05	
785.6524_5.59	SM(d18:1/22:1)	0.07508	0.01524	0.05675	0.02314	0.05705	0.00704	1.01		0.04583	0.00953	0.81		0.04484	0.01229	0.79		0.06085	0.00819	1.07	
752.6711_7.71	GlcCer(d18:2/20:1)	0.00428	0.00016	0.00403	0.00032	0.00419	0.00020	1.04		0.00439	0.00029	1.09		0.00434	0.00038	1.08		0.00379	0.00012	0.94	
605.5498_5.23	DG(34:0)	9903.65	1403.83	40210.80	45573.57	52294.74	14232.50	1.30		50631.11	9408.52	1.26		44977.45	5483.53	1.12		36169.60	1842.39	0.90	
610.5403_5.87	DG(34:2)	766182.52	77112.37	725012.89	41230.11	657603.54	53082.38	0.91		614236.72	28518.89	0.85		583021.40	39439.12	0.80		653493.49	72718.17	0.90	
633.543_6.62	DG(35:0)	61634.26	4264.27	66417.66	7448.77	55553.81	3875.92	0.84		51954.70	3406.13	0.78		46408.40	3992.44	0.70		58320.71	9206.18	0.88	
619.5268_6.38	DG(36:3)	555743.02	87139.82	353780.42	19007.23	341483.04	58325.42	0.97		387675.62	39289.98	1.10		422995.73	69879.04	1.20		354504.44	43587.44	1.00	
661.5736_6.92	DG(37:0)	0.00400	0.00034	0.00476	0.00031	0.00421	0.00017	0.88		0.00434	0.00039	0.91		0.00464	0.00028	0.98		0.00389	0.00017	0.82	
637.4824_5.36	DG(38:8)	30099.83	2332.90	26157.16	5377.77	21316.38	3473.67	0.81		18990.96	4959.44	0.73		19370.40	3336.49	0.74		22448.71	2253.39	0.86	
682.5424_5.33	DG(40:8)	93753.72	12876.14	75332.50	12311.93	64150.20	15600.62	0.85		51300.60	7558.97	0.68		67414.30	7493.41	0.89		73173.44	10393.47	0.97	
482.3241_1.06	LPC(15:0)	0.01752	0.00239	0.02176	0.00924	0.02904	0.00406	1.33		0.02829	0.00122	1.30		0.02739	0.00428	1.26		0.02471	0.00356	1.14	
482.3242_1.9	LPC(15:0)	0.01272	0.00341	0.00946	0.00441	0.01502	0.00392	1.59	•	0.01661	0.00139	1.76	•	0.01736	0.00159	1.84	•	0.01602	0.00293	1.69	•
518.3242_0.9	LPC(18:3)	0.02386	0.00406	0.03469	0.01873	0.05129	0.00845	1.48		0.04878	0.00229	1.41		0.04605	0.00788	1.33		0.03864	0.00250	1.11	
550.3871_1.88	LPC(20:1)	0.02487	0.00444	0.02957	0.01630	0.03999	0.00604	1.35		0.03850	0.00531	1.30		0.03422	0.00409	1.16		0.03066	0.00431	1.04	
572.3694_1.88	LPC(22:4)	0.00315	0.00052	0.00414	0.00079	0.00458	0.00046	1.11		0.00463	0.00068	1.12		0.00488	0.00016	1.18		0.00407	0.00069	0.98	
709.5803_4.6	PC(30:0)	0.00145	0.00020	0.00167	0.00023	0.00167	0.00014	1.00		0.00191	0.00015	1.14		0.00197	0.00025	1.18		0.00181	0.00026	1.08	
720.5539_4.9	PC(31:0)	0.00189	0.00032	0.00230	0.00101	0.00304	0.00045	1.32		0.00305	0.00031	1.33		0.00319	0.00065	1.39		0.00293	0.00058	1.28	
716.5230_4.97	PC(31:2)	0.00609	0.00065	0.00515	0.00095	0.00728	0.00151	1.41		0.00949	0.00092	1.84	•	0.00932	0.00100	1.81		0.00734	0.00154	1.42	
716.562_4.70	PC(32:2)	0.00368	0.00050	0.00489	0.00123	0.00602	0.00027	1.23		0.00681	0.00044	1.39		0.00707	0.00103	1.44		0.00619	0.00104	1.27	
730.5381_4.36	PC(32:2)	0.00295	0.00059	0.00333	0.00161	0.00495	0.00082	1.49	•	0.00573	0.00059	1.72	•	0.00550	0.00065	1.65	•	0.00511	0.00124	1.54	•
748.5831_5.33	PC(33:0)	0.00056	0.00014	0.00053	0.00029	0.00078	0.00009	1.46		0.00090	0.00014	1.68	•	0.00092	0.00016	1.72	•	0.00068	0.00011	1.27	
730.5758_4.9	PC(33:1)	0.00048	0.00017	0.00065	0.00032	0.00075	0.00014	1.16		0.00102	0.00014	1.57	•	0.00083	0.00009	1.29	•	0.00077	0.00029	1.19	
746.5689_4.96	PC(33:1)	0.00508	0.00092	0.00459	0.00247	0.00617	0.00099	1.34		0.00683	0.00043	1.49	•	0.00672	0.00080	1.46		0.00607	0.00069	1.32	
728.5605_5.62	PC(33:2)	0.00121	0.00029	0.00101	0.00028	0.00133	0.00040	1.32		0.00164	0.00010	1.63	•	0.00162	0.00020	1.60	•	0.00143	0.00027	1.42	
744.5558_5.41	PC(33:2)	0.00678	0.00157	0.00548	0.00282	0.00945	0.00279	1.72		0.01249	0.00155	2.28	•	0.01238	0.00136	2.26	•	0.00863	0.00167	1.57	•
742.5373_4.36	PC(33:3)	0.00202	0.00021	0.00232	0.00024	0.00253	0.00021	1.09		0.00265	0.00012	1.14		0.00263	0.00036	1.13		0.00245	0.00029	1.06	
740.5237_4.69	PC(33:4)	0.00147	0.00045	0.00144	0.00051	0.00443	0.00163	3.08	•	0.00578	0.00081	4.02	•	0.00527	0.00080	3.67	•	0.00451	0.00259	3.13	•
762.5997_5.55	PC(34:0)	0.00770	0.00147	0.01014	0.00616	0.01034	0.00128	1.02		0.01213	0.00104	1.20		0.01087	0.00081	1.07		0.01114	0.00105	1.10	
742.5691_5.06	PC(34:2)	0.00199	0.00040	0.00230	0.00132	0.00443	0.00081	1.93	•	0.00549	0.00056	2.39	•	0.00505	0.00053	2.20	•	0.00399	0.00078	1.74	•
740.5593_4.6	PC(34:3)	0.00125	0.00018	0.00159	0.00045	0.00215	0.00029	1.35		0.00244	0.00021	1.53	•	0.00242	0.00022	1.52	•	0.00190	0.00026	1.20	
756.5543_4.49	PC(34:3)	0.05571	0.01363	0.05162	0.02766	0.08211	0.01617	1.59	•	0.09336	0.00691	1.81	•	0.09127	0.01212	1.77	•	0.07812	0.01522	1.51	•
738.546_4.25	PC(34:4)	0.00069	0.00011	0.00071	0.00011	0.00099	0.00011	1.39		0.00116	0.00013	1.63	•	0.00121	0.00014	1.69	•	0.00094	0.00013	1.32	
754.5333_4.29	PC(34:4)	0.00244	0.00032	0.00252	0.00076	0.00359	0.00042	1.42		0.00413	0.00016	1.64	•	0.00406	0.00063	1.61	•	0.00362	0.00065	1.44	
772.5851_5.08	PC(35:2)	0.03386	0.00652	0.03215	0.01629	0.05623	0.00808	1.75	•	0.06009	0.00396	1.87	•	0.06003	0.00801	1.87	•	0.05253	0.00537	1.63	•
770.5692_4.70	PC(35:3)	0.01222	0.00256	0.01240	0.00619	0.02379	0.00428	1.92	•	0.02734	0.00297	2.20	•	0.02734	0.00391	2.20	•	0.02053	0.00247	1.66	•
754.5719_5.62	PC(35:4)	0.00493	0.00055	0.00533	0.00088	0.00600	0.00087	1.13		0.00700	0.00064	1.31		0.00756	0.00100	1.42		0.00602	0.00109	1.13	
768.5540_5.36	PC(35:4)	0.00752	0.00206	0.00424	0.00201	0.00735	0.00213	1.74	•	0.00907	0.00090	2.14	•	0.01023	0.00122	2.41	•	0.00810	0.00172	1.91	•
766.5427_5.09	PC(35:5)	0.00084	0.00020	0.00071	0.00032	0.00116	0.00042	1.64	•	0.00141	0.00012	2.00	•	0.00158	0.00021	2.24	•	0.00133	0.00031	1.88	•
764.5231_4.83	PC(35:6)	0.02078	0.00813	0.00991	0.00582	0.01869	0.00607	1.89	•	0.02341	0.00251	2.36	•	0.03222	0.00710	3.25	•	0.01950	0.00446	1.97	•
786.6002_5.30	PC(36:2)	1.78475	0.58892	2.20218	1.35733	3.56345	0.57707	1.62	•	3.93336	0.58773	1.79	•	3.75296	0.38643	1.70	•	3.47608	0.31512	1.58	•
766.57_4.99	PC(36:4)	0.00949	0.00135	0.00935	0.00234	0.01293	0.00144	1.38		0.01458	0.00074	1.56	•	0.01475	0.00091	1.58	•	0.01143	0.00194	1.22	
766.5719_4.78	PC(36:4)	0.00500	0.00111	0.00453	0.00204	0.00716	0.00177	1.58	•	0.00870	0.00097	1.92	•	0.00904	0.00062	1.99	•	0.00754	0.00197	1.66	•
782.56939_4.57	PC(36:4)	0.08602	0.02145	0.10634	0.06640	0.33036	0.07482	3.11	•	0.40122	0.04137	3.77	•	0.36702	0.05771	3.45	•	0.21236	0.03000	2.00	•
782.5694_4.82	PC(36:4)	1.33658	0.58337	0.98068	0.55469	1.69049	0.46788	1.72	•	1.84909	0.15297	1.89	•	1.99603	0.34417	2.04	•	1.75526	0.30468	1.79	•
788.5521_4.99	PC(36:4)	0.00038	0.00013	0.00036	0.00024	0.00071	0.00017	1.99	•	0.00079	0.00006	2.20	•	0.00085	0.00012	2.38	•	0.00069	0.00016	1.92	•
780.5540_4.22	PC(36:5)	0.00861	0.00185	0.00791	0.00443	0.02328	0.00315	2.94	•	0.02542	0.00175	3.21	•	0.02349	0.00467	2.97	•	0.01814	0.00378	2.29	•
781.5896_5.93	PC(36:5)	0.00776	0.00068	0.00750	0.00134	0.00834	0.00085	1.11		0.00993	0.00043	1.32		0.01022	0.00139	1.36		0.00864	0.00076	1.15	
778.5362_4.54	PC(36:6)	0.00167	0.00062	0.00161	0.00093	0.00333	0.00110	2.07	•	0.00393	0.00059	2.44	•	0.00398	0.00056	2.48	•	0.00285	0.00079	1.77	•
798.60152_5.12	PC(37:3)	0.00565	0.00118	0.00555	0.00256	0.00824	0.00149	1.49	•	0.00965	0.00118	1.74	•	0.00887	0.00092	1.60	•	0.00780	0.00089	1.41	
813.6184_5.33	PC(37:4)	0.03054	0.00718	0.02926	0.01653	0.04426	0.00795	1.51	•	0.05096	0.00948	1.74	•	0.04932	0.00773	1.69	•	0.04439	0.00448	1.52	•
794.5709_4.72	PC(37:5)	0.00752	0.00162	0.00720	0.00243	0.01030	0.00158	1.43		0.01234	0.00122	1.72	•	0.01223	0.00166	1.70	•	0.01260	0.00285	1.75	•
790.5744_4.89	PC(38:6)	0.01307	0.00212	0.01505	0.00689	0.02108	0.00475	1.40		0.02421	0.00100	1.61	•	0.02031	0.00418	1.35		0.01912	0.00104	1.27	
806.5698_4.70	PC(38:6)	13.77161	4.81230	11.73420	6.85051	11.55595	2.09356	0.98		9.85537	0.89451	0.84		10.70199	2.45626	0.91		16.46454	0.92749	1.40	
804.552_4.57	PC(38:7)	0.02174	0.00432	0.02278	0.00888	0.03830	0.00703	1.68	•	0.04499	0.00483	1.97	•	0.04193	0.00551	1.84	•	0.03442	0.00483	1.51	•
802.5358_4.49	PC(38:8)	0.00245	0.00065	0.00245	0.00100	0.00391	0.00101	1.59	•	0.00462	0.00088	1.89	•	0.00496	0.00068	2.02	•	0.00487	0.00103	1.99	•
824.6168_5.45	PC(39:4)	0.00653	0.00093	0.00566	0.00232	0.00745	0.00115	1.32		0.00762	0.00067	1.35		0.00794	0.00132	1.40		0.00848	0.00047	1.50	•
830.5662_4.97	PC(40:8)	0.00770	0.00094	0.00680	0.00404	0.00787	0.00105	1.16		0.00959	0.00136	1.41		0.00888	0.00093	1.31		0.01090	0.00077	1.60	•
830.5694_4.39	PC(40:8)	0.01671	0.00384	0.01482	0.00841	0.02422	0.00304	1.63	•	0.02502	0.00165	1.69	•	0.02363	0.00393	1.59	•	0.02618	0.00269	1.77	•
847.6036_4.55	PC(40:8)	0.00326	0.00013	0.00364	0.00029	0.00393	0.00032	1.08		0.00407	0.00026	1.12		0.00396	0.00042	1.09		0.00395	0.00034	1.09	
828.5589_4.1	PC(40:9)	0.00121	0.00035	0.00155	0.00037	0.00164	0.00015	1.06		0.00204	0.00027	1.32		0.00186	0.00013	1.21		0.00197	0.00035	1.28	
873.7503_7.15	PC(42:1)	0.00508	0.00058	0.00509	0.00101	0.00541	0.00146	1.06		0.00751	0.00099	1.47		0.00772	0.00086	1.52	•	0.00624	0.00176	1.23	
852.6819_6.16	PC(42:4)	0.00305	0.00038	0.00425	0.00085	0.00384	0.00040	0.90		0.00405	0.00046	0.95		0.00425	0.00094	1.00		0.00375	0.00033	0.88	
822.5994_4.67	PC(42:5)	0.00542	0.00123	0.00630	0.00169	0.00895	0.00166	1.42		0.00959	0.00061	1.52	•	0.00962	0.00169	1.53	•	0.00828	0.00109	1.31	
852.5592_4.46	PC(42:5)	0.00451	0.00094	0.00530	0.00255	0.00774	0.00141	1.46		0.00867	0.00079	1.63	•	0.00848	0.00132	1.60	•	0.00832	0.00083	1.57	•
846.6366_5.72	PC(42:6)	0.00096	0.00018	0.00119	0.00060	0.00103	0.00011	0.87		0.00082	0.00016	0.69		0.00101	0.00023	0.85		0.00136	0.00021	1.15	
858.6002_4.83	PC(42:8)	0.00135	0.00029	0.00114	0.00069	0.00192	0.00036	1.68	•	0.00233	0.00066	2.03	•	0.00203	0.00046	1.77	•	0.00148	0.00026	1.30	
880.5906_4.91	PC(42:8)	0.00523	0.00063	0.00849	0.00472	0.01301	0.00251	1.53	•	0.01409	0.00168	1.66	•	0.01214	0.00205	1.43		0.01161	0.00109	1.37	
856.5836_4.47	PC(42:9)	0.00100	0.00028	0.00109	0.00053	0.00166	0.00030	1.52	•	0.00204	0.00022	1.86	•	0.00194	0.00023	1.78	•	0.00182	0.00014	1.66	•
854.5698_4.31	PC(42:10)	0.00314	0.00089	0.00235	0.00147	0.00391	0.00059	1.67	•	0.00397	0.00016	1.69	•	0.00392	0.00087	1.67	•	0.00478	0.00088	2.04	•
904.5912_4.88	PC(44:10)	0.04932	0.00847	0.06070	0.03937	0.05628	0.01004	0.93		0.04945	0.00332	0.81		0.04295	0.00839	0.71		0.07776	0.00493	1.28	
902.5751_4.6	PC(44:11)	0.00087	0.00027	0.00168	0.00107	0.00207	0.00056	1.24		0.00241	0.00044	1.43		0.00211	0.00049	1.26		0.00261	0.00033	1.56	•
902.5758_4.47	PC(44:11)	0.00154	0.00060	0.00092	0.00065	0.00179	0.00038	1.94	•	0.00199	0.00024	2.16	•	0.00195	0.00047	2.11	•	0.00151	0.00013	1.63	•
878.5758_4.53	PC(44:12)	0.00124	0.00033	0.00159	0.00080	0.00237	0.00050	1.49	•	0.00268	0.00036	1.68	•	0.00249	0.00052	1.56	•	0.00245	0.00026	1.54	•
935.7295_6.47	PC(46:6)	0.00600	0.00026	0.00704	0.00082	0.00641	0.00053	0.91		0.00734	0.00102	1.04		0.00749	0.00096	1.06		0.00648	0.00039	0.92	

Supplementary Table 8: ESI+ mode lipids at Day 1 with IR fold changes compared to control

m/z_Ret time	ID	C_D1 Average	0_D1 Average	Fold change to Control		5_D1 Average	Fold change to Control		15_D1 Average	Fold change to Control		25_D1 Average	Fold change to Control		83_D1 Average	Fold change to Control	
666.6184_7.8	CE(18:2)	417580.61	578318.43	1.38		667761.12	1.60	●	588924.87	1.41		1186368.91	2.84	●	584241.36	1.40	
690.6185_7.67	CE(20:4)	593469.68	723371.02	1.22		897870.62	1.51	●	704063.21	1.19		1798903.91	3.03	●	724667.61	1.22	
671.5729_7.97	CE(20:5)	3766.19	4107.61	1.09		4496.47	1.19		4089.26	1.09		44189.15	11.73	●	4718.04	1.25	
688.6024_7.51	CE(20:5)	107699.38	148245.70	1.38		131654.44	1.22		123603.66	1.15		213726.43	1.98	●	115866.16	1.08	
719.5759_7.99	CE(22:6)	648.53	1046.09	1.61	●	1748.47	2.70	●	1861.91	2.87	●	12488.77	19.26	●	1651.04	2.55	●
570.5478_4.71	Cer(d18:0/h17:0)	0.47632	0.48555	1.02		0.43755	0.92		0.51359	1.08		0.56172	1.18		0.39282	0.82	
570.5466_5.08	Cer(d18:0/h17:0)	0.00891	0.00860	0.97		0.01028	1.15		0.00877	0.99		0.00807	0.91		0.00988	1.11	
750.6474_5.56	SM(d18:1/18:0)	0.02923	0.03734	1.28		0.03600	1.23		0.03396	1.16		0.03088	1.06		0.03797	1.30	
759.6369_5.52	SM(d18:1/20:0)	0.01766	0.02279	1.29		0.01997	1.13		0.01678	0.95		0.01511	0.86		0.01980	1.12	
785.6524_5.59	SM(d18:1/22:1)	0.06827	0.07930	1.16		0.05624	0.82		0.05891	0.86		0.04907	0.72		0.05937	0.87	
752.6711_7.71	GlcCer(d18:2/20:1)	0.00415	0.00414	1.00		0.00421	1.01		0.00464	1.12		0.00422	1.02		0.00446	1.07	
605.5498_5.23	DG(34:0)	23427.47	25344.34	1.08		29385.98	1.25		33344.98	1.42		36857.98	1.57	●	27525.91	1.17	
610.5403_5.87	DG(34:2)	727875.15	596816.61	0.82		716532.39	0.98		677802.49	0.93		657609.87	0.90		648172.93	0.89	
633.543_6.62	DG(35:0)	51083.32	49755.38	0.97		57274.53	1.12		50984.32	1.00		51631.71	1.01		55324.41	1.08	
619.5268_6.38	DG(36:3)	541210.69	285191.77	0.53	●	339046.02	0.63	●	308508.07	0.57	●	292413.26	0.54	●	314505.44	0.58	●
661.5736_6.92	DG(37:0)	0.00418	0.00436	1.04		0.00437	1.04		0.00507	1.21		0.00433	1.04		0.00455	1.09	
637.4824_5.36	DG(38:8)	26052.92	20020.14	0.77		26396.71	1.01		21644.98	0.83		24408.18	0.94		23159.86	0.89	
682.5424_5.33	DG(40:8)	103991.57	69972.88	0.67	●	78577.92	0.76		66922.49	0.64	●	56785.81	0.55	●	77183.43	0.74	
482.3241_1.06	LPC(15:0)	0.02386	0.03042	1.28		0.02465	1.03		0.02726	1.14		0.02561	1.07		0.02657	1.11	
482.3242_1.9	LPC(15:0)	0.01605	0.01704	1.06		0.01354	0.84		0.01510	0.94		0.01531	0.95		0.01367	0.85	
518.3242_0.9	LPC(18:3)	0.03215	0.04543	1.41		0.03773	1.17		0.04752	1.48		0.04804	1.49		0.04298	1.34	
550.3871_1.88	LPC(20:1)	0.03320	0.04560	1.37		0.03737	1.13		0.04065	1.22		0.03670	1.11		0.03746	1.13	
572.3694_1.88	LPC(22:4)	0.00380	0.00556	1.46		0.00508	1.34		0.00547	1.44		0.00464	1.22		0.00500	1.32	
709.5803_4.6	PC(30:0)	0.00183	0.00204	1.12		0.00185	1.01		0.00193	1.06		0.00199	1.09		0.00196	1.07	
720.5539_4.9	PC(31:0)	0.00231	0.00237	1.03		0.00248	1.08		0.00288	1.25		0.00320	1.39		0.00280	1.21	
716.5230_4.97	PC(31:2)	0.00779	0.00810	1.04		0.00635	0.81		0.00691	0.89		0.00690	0.89		0.00620	0.80	
716.562_4.70	PC(32:2)	0.00485	0.00574	1.18		0.00609	1.26		0.00663	1.37		0.00697	1.44		0.00624	1.28	
730.5381_4.36	PC(32:2)	0.00421	0.00667	1.58		0.00396	0.94		0.00511	1.21		0.00564	1.34		0.00587	1.39	
748.5831_5.33	PC(33:0)	0.00065	0.00071	1.09		0.00055	0.84		0.00065	1.00		0.00078	1.20		0.00065	0.99	
730.5758_4.9	PC(33:1)	0.00068	0.00073	1.07		0.00064	0.95		0.00068	1.00		0.00080	1.19		0.00071	1.06	
746.5689_4.96	PC(33:1)	0.00728	0.00759	1.04		0.00522	0.72		0.00625	0.86		0.00617	0.85		0.00630	0.86	
728.5605_5.62	PC(33:2)	0.00130	0.00136	1.05		0.00118	0.91		0.00112	0.86		0.00118	0.90		0.00112	0.86	
744.5558_5.41	PC(33:2)	0.01059	0.01062	1.00		0.00992	0.94		0.00983	0.93		0.01035	0.98		0.00968	0.91	
742.5373_4.36	PC(33:3)	0.00221	0.00252	1.14		0.00262	1.18		0.00275	1.25		0.00265	1.20		0.00290	1.31	
740.5237_4.69	PC(33:4)	0.00319	0.00457	1.43		0.00382	1.20		0.00530	1.66	●	0.00548	1.72		0.00469	1.47	
762.5997_5.55	PC(34:0)	0.00883	0.01145	1.30		0.01078	1.22		0.01196	1.35		0.01240	1.40		0.01303	1.48	
742.5691_5.06	PC(34:2)	0.00320	0.00418	1.31		0.00290	0.91		0.00377	1.18		0.00360	1.13		0.00305	0.95	
740.5593_4.6	PC(34:3)	0.00174	0.00243	1.40		0.00235	1.36		0.00242	1.39		0.00249	1.43		0.00221	1.27	
756.5543_4.49	PC(34:3)	0.07544	0.10045	1.33		0.05945	0.79		0.07944	1.05		0.08368	1.11		0.08179	1.08	
738.546_4.25	PC(34:4)	0.00088	0.00105	1.19		0.00093	1.06		0.00093	1.05		0.00095	1.08		0.00094	1.07	
754.5333_4.29	PC(34:4)	0.00313	0.00428	1.36		0.00277	0.89		0.00342	1.09		0.00370	1.18		0.00382	1.22	
772.5851_5.08	PC(35:2)	0.05168	0.05856	1.13		0.04719	0.91		0.05827	1.13		0.06062	1.17		0.05709	1.10	
770.5692_4.70	PC(35:3)	0.02050	0.02625	1.28		0.01965	0.96		0.02513	1.23		0.02540	1.24		0.02376	1.16	
754.5719_5.62	PC(35:4)	0.00507	0.00537	1.06		0.00579	1.14		0.00527	1.04		0.00532	1.05		0.00564	1.11	
768.5540_5.36	PC(35:4)	0.00947	0.00975	1.03		0.00833	0.88		0.00711	0.75		0.00750	0.79		0.00901	0.95	
766.5427_5.09	PC(35:5)	0.00152	0.00148	0.98		0.00125	0.82		0.00122	0.80		0.00129	0.85		0.00145	0.96	
764.5231_4.83	PC(35:6)	0.02714	0.03144	1.16		0.01672	0.62	●	0.01661	0.61	●	0.01624	0.60	●	0.02357	0.87	
786.6002_5.30	PC(36:2)	2.84450	3.20032	1.13		2.55958	0.90		3.35955	1.18		3.73882	1.31		2.98701	1.05	
766.57_4.99	PC(36:4)	0.01202	0.01245	1.04		0.01104	0.92		0.01201	1.00		0.01122	0.93		0.01170	0.97	
766.5719_4.78	PC(36:4)	0.00753	0.01034	1.37		0.00688	0.91		0.00882	1.17		0.00826	1.10		0.00941	1.25	
782.56939_4.57	PC(36:4)	0.16983	0.27152	1.60	●	0.17590	1.04		0.28261	1.66	●	0.34853	2.05	●	0.26843	1.58	●
782.5694_4.82	PC(36:4)	1.67834	2.35094	1.40		1.34471	0.80		1.70500	1.02		1.97907	1.18		2.14849	1.28	
788.5521_4.99	PC(36:4)	0.00060	0.00069	1.15		0.00029	0.49	●	0.00035	0.60	●	0.00044	0.74		0.00035	0.60	●
780.5540_4.22	PC(36:5)	0.01384	0.02439	1.76		0.01285	0.93		0.02035	1.47		0.02356	1.70	●	0.02238	1.62	●
781.5896_5.93	PC(36:5)	0.00805	0.00777	0.97		0.00776	0.96		0.00739	0.92		0.00781	0.97		0.00787	0.98	
778.5362_4.54	PC(36:6)	0.00280	0.00422	1.51	●	0.00199	0.71		0.00295	1.05		0.00352	1.26		0.00293	1.05	
798.60152_5.12	PC(37:3)	0.00887	0.01059	1.19		0.00805	0.91		0.01070	1.21		0.01095	1.23		0.00974	1.10	
813.6184_5.33	PC(37:4)	0.04112	0.04938	1.20		0.03936	0.96		0.04337	1.05		0.04505	1.10		0.04573	1.11	
794.5709_4.72	PC(37:5)	0.01161	0.01450	1.25		0.00974	0.84		0.01193	1.03		0.01242	1.07		0.01394	1.20	
790.5744_4.89	PC(38:6)	0.01703	0.02321	1.36		0.01599	0.94		0.02106	1.24		0.02130	1.25		0.01982	1.16	
806.5698_4.70	PC(38:6)	15.96005	20.84641	1.31		11.65760	0.73		13.52152	0.85		12.28556	0.77		16.93467	1.06	
804.552_4.57	PC(38:7)	0.03121	0.04268	1.37		0.02494	0.80		0.03606	1.16		0.04106	1.32		0.03761	1.21	
802.5358_4.49	PC(38:8)	0.00417	0.00560	1.34		0.00277	0.67	●	0.00417	1.00		0.00485	1.16		0.00520	1.25	
824.6168_5.45	PC(39:4)	0.00780	0.00934	1.20		0.00872	1.12		0.00848	1.09		0.00879	1.13		0.01053	1.35	
830.5662_4.97	PC(40:8)	0.01044	0.01077	1.03		0.00745	0.71		0.00883	0.85		0.00914	0.87		0.01025	0.98	
830.5694_4.39	PC(40:8)	0.02277	0.03179	1.40		0.02052	0.90		0.02622	1.15		0.02699	1.19		0.02999	1.32	
847.6036_4.55	PC(40:8)	0.00343	0.00381	1.11		0.00382	1.12		0.00406	1.18		0.00366	1.07		0.00423	1.23	
828.5589_4.1	PC(40:9)	0.00149	0.00246	1.65	●	0.00185	1.24		0.00220	1.47		0.00201	1.34		0.00254	1.70	
873.7503_7.15	PC(42:1)	0.00588	0.00592	1.01		0.00636	1.08		0.00617	1.05		0.00699	1.19		0.00626	1.07	
852.6819_6.16	PC(42:4)	0.00329	0.00405	1.23		0.00412	1.25		0.00391	1.19		0.00376	1.14		0.00410	1.25	
822.5994_4.67	PC(42:5)	0.00798	0.00987	1.24		0.00863	1.08		0.00963	1.21		0.00855	1.07		0.00902	1.13	
852.5592_4.46	PC(42:5)	0.00615	0.00915	1.49		0.00573	0.93		0.00700	1.14		0.00707	1.15		0.00765	1.24	
846.6366_5.72	PC(42:6)	0.00110	0.00124	1.13		0.00136	1.24		0.00119	1.08		0.00115	1.04		0.00139	1.26	
858.6002_4.83	PC(42:8)	0.00256	0.00305	1.19		0.00233	0.91		0.00267	1.04		0.00230	0.90		0.00307	1.20	
880.5906_4.91	PC(42:8)	0.00818	0.00983	1.20		0.00817	1.00		0.01055	1.29		0.01051	1.29		0.00777	0.95	
856.5836_4.47	PC(42:9)	0.00160	0.00232	1.45		0.00159	1.00		0.00207	1.30		0.00200	1.25		0.00215	1.35	
854.5698_4.31	PC(42:10)	0.00383	0.00561	1.47		0.00333	0.87		0.00408	1.07		0.00462	1.21		0.00559	1.46	
904.5912_4.88	PC(44:10)	0.04943	0.06321	1.28		0.06150	1.24		0.06696	1.35		0.06209	1.26		0.07856	1.59	●
902.5751_4.6	PC(44:11)	0.00188	0.00312	1.66	●	0.00215	1.15		0.00273	1.45		0.00243	1.29		0.00292	1.55	●
902.5758_4.47	PC(44:11)	0.00237	0.00305	1.28		0.00202	0.85		0.00201	0.85		0.00183	0.77		0.00254	1.07	
878.5758_4.53	PC(44:12)	0.00203	0.00323	1.59	●	0.00240	1.18		0.00296	1.46		0.00247	1.22		0.00286	1.41	
935.7295_6.47	PC(46:6)	0.00653	0.00680	1.04		0.00682	1.04		0.00675	1.03		0.00643	0.99		0.00668	1.02	

Supplementary Table 9: ESI+ mode lipids at Day 7 with IR fold changes compared to control

m/z_Ret time	ID	C_D7 Average	0_D7 Average	Fold change to Control		5_D7 Average	Fold change to Control		15_D7 Average	Fold change to Control		25_D7 Average	Fold change to Control		83_D7 Average	Fold change to Control	
666.6184_7.8	CE(18:2)	456944.68	313267.04	0.69		394573.61	0.86		417823.43	0.91		335622.36	0.73		1602376.12	3.51	•
690.6185_7.67	CE(20:4)	730098.27	379645.04	0.52	•	509044.50	0.70		588028.32	0.81		511827.73	0.70		2254112.54	3.09	•
671.5729_7.97	CE(20:5)	3307.64	3612.24	1.09		2957.22	0.89		2920.58	0.88		2261.53	0.68		72610.84	21.95	•
688.6024_7.51	CE(20:5)	86301.08	68627.73	0.80		67923.10	0.79		109161.74	1.26		95116.24	1.10		249946.79	2.90	•
719.5759_7.99	CE(22:6)	1225.98	1983.47	1.62	•	1182.28	0.96		627.85	0.51	•	1063.63	0.87		21599.29	17.62	•
570.5478_4.71	Cer(d18:0/h17:0)	0.45991	0.56835	1.24		0.63719	1.39		0.55752	1.21		0.51392	1.12		0.64600	1.40	
570.5466_5.08	Cer(d18:0/h17:0)	0.00912	0.01109	1.22		0.00859	0.94		0.00778	0.85		0.00811	0.89		0.00929	1.02	
750.6474_5.56	SM(d18:1/18:0)	0.02927	0.04063	1.39		0.03309	1.13		0.03215	1.10		0.03245	1.11		0.03358	1.15	
759.6369_5.52	SM(d18:1/20:0)	0.02125	0.01655	0.78		0.01418	0.67	•	0.01115	0.52	•	0.01251	0.59	•	0.01731	0.81	
785.6524_5.59	SM(d18:1/22:1)	0.07508	0.05675	0.76		0.05705	0.76		0.04583	0.61	•	0.04484	0.60	•	0.06085	0.81	
752.6711_7.71	GlcCer(d18:2/20:1)	0.00428	0.00403	0.94		0.00419	0.98		0.00439	1.03		0.00434	1.01		0.00379	0.89	
605.5498_5.23	DG(34:0)	9903.65	40210.80	4.06	•	52294.74	5.28	•	50631.11	5.11	•	44977.45	4.54	•	36169.60	3.65	•
610.5403_5.87	DG(34:2)	766182.52	725012.89	0.95		657603.54	0.86		614236.72	0.80		583021.40	0.76		653493.49	0.85	
633.543_6.62	DG(35:0)	61634.26	66417.66	1.08		55553.81	0.90		51954.70	0.84		46408.40	0.75		58320.71	0.95	
619.5268_6.38	DG(36:3)	555743.02	353780.42	0.64	•	341483.04	0.61	•	387675.62	0.70		422995.73	0.76		354504.44	0.64	•
661.5736_6.92	DG(37:0)	0.00400	0.00476	1.19		0.00421	1.05		0.00434	1.08		0.00464	1.16		0.00389	0.97	
637.4824_5.36	DG(38:8)	30099.83	26157.16	0.87		21316.38	0.71		18990.96	0.63	•	19370.40	0.64	•	22448.71	0.75	
682.5424_5.33	DG(40:8)	93753.72	75332.50	0.80		64150.20	0.68		51300.60	0.55	•	67414.30	0.72		73173.44	0.78	
482.3241_1.06	LPC(15:0)	0.01752	0.02176	1.24		0.02904	1.66	•	0.02829	1.61	•	0.02739	1.56	•	0.02471	1.41	
482.3242_1.9	LPC(15:0)	0.01272	0.00946	0.74		0.01502	1.18		0.01661	1.31		0.01736	1.37		0.01602	1.26	
518.3242_0.9	LPC(18:3)	0.02386	0.03469	1.45		0.05129	2.15	•	0.04878	2.04	•	0.04605	1.93	•	0.03864	1.62	•
550.3871_1.88	LPC(20:1)	0.02487	0.02957	1.19		0.03999	1.61	•	0.03850	1.55	•	0.03422	1.38		0.03066	1.23	
572.3694_1.88	LPC(22:4)	0.00315	0.00414	1.31		0.00458	1.46		0.00463	1.47		0.00488	1.55	•	0.00407	1.29	
709.5803_4.6	PC(30:0)	0.00145	0.00167	1.15		0.00167	1.15		0.00191	1.32		0.00197	1.36		0.00181	1.25	
720.5539_4.9	PC(31:0)	0.00189	0.00230	1.22		0.00304	1.61	•	0.00305	1.62	•	0.00319	1.69	•	0.00293	1.55	•
716.5230_4.97	PC(31:2)	0.00609	0.00515	0.85		0.00728	1.19		0.00949	1.56	•	0.00932	1.53	•	0.00734	1.20	
716.562_4.70	PC(32:2)	0.00368	0.00489	1.33		0.00602	1.64	•	0.00681	1.85	•	0.00707	1.92	•	0.00619	1.68	•
730.5381_4.36	PC(32:2)	0.00295	0.00333	1.13		0.00495	1.68	•	0.00573	1.94	•	0.00550	1.86	•	0.00511	1.73	
748.5831_5.33	PC(33:0)	0.00056	0.00053	0.95		0.00078	1.40		0.00090	1.61	•	0.00092	1.65	•	0.00068	1.21	
730.5758_4.9	PC(33:1)	0.00048	0.00065	1.35		0.00075	1.57	•	0.00102	2.12	•	0.00083	1.74	•	0.00077	1.61	•
746.5689_4.96	PC(33:1)	0.00508	0.00459	0.90		0.00617	1.22		0.00683	1.35		0.00672	1.32		0.00607	1.20	
728.5605_5.62	PC(33:2)	0.00121	0.00101	0.83		0.00133	1.09		0.00164	1.35		0.00162	1.33		0.00143	1.18	
744.5558_5.41	PC(33:2)	0.00678	0.00548	0.81		0.00945	1.39		0.01249	1.84	•	0.01238	1.83		0.00863	1.27	
742.5373_4.36	PC(33:3)	0.00202	0.00232	1.15		0.00253	1.25		0.00265	1.31		0.00263	1.30		0.00245	1.22	
740.5237_4.69	PC(33:4)	0.00147	0.00144	0.98		0.00443	3.01	•	0.00578	3.92	•	0.00527	3.58	•	0.00451	3.06	•
762.5997_5.55	PC(34:0)	0.00770	0.01014	1.32		0.01034	1.34		0.01213	1.57	•	0.01087	1.41		0.01114	1.45	
742.5691_5.06	PC(34:2)	0.00199	0.00230	1.16		0.00443	2.23	•	0.00549	2.76	•	0.00505	2.54	•	0.00399	2.01	•
740.5593_4.6	PC(34:3)	0.00125	0.00159	1.27		0.00215	1.72	•	0.00244	1.95	•	0.00242	1.93	•	0.00190	1.52	•
756.5543_4.49	PC(34:3)	0.05571	0.05162	0.93		0.08211	1.47		0.09336	1.68	•	0.09127	1.64	•	0.07812	1.40	
738.546_4.25	PC(34:4)	0.00069	0.00071	1.03		0.00099	1.43		0.00116	1.68	•	0.00121	1.74	•	0.00094	1.36	
754.5333_4.29	PC(34:4)	0.00244	0.00252	1.03		0.00359	1.47		0.00413	1.69	•	0.00406	1.66	•	0.00362	1.49	
772.5851_5.08	PC(35:2)	0.03386	0.03215	0.95		0.05623	1.66	•	0.06009	1.77	•	0.06003	1.77	•	0.05253	1.55	•
770.5692_4.70	PC(35:3)	0.01222	0.01240	1.02		0.02379	1.95	•	0.02734	2.24	•	0.02734	2.24	•	0.02053	1.68	•
754.5719_5.62	PC(35:4)	0.00493	0.00533	1.08		0.00600	1.22		0.00700	1.42		0.00756	1.53	•	0.00602	1.22	
768.5540_5.36	PC(35:4)	0.00752	0.00424	0.56	•	0.00735	0.98		0.00907	1.21		0.01023	1.36		0.00810	1.08	
766.5427_5.09	PC(35:5)	0.00084	0.00071	0.84		0.00116	1.37		0.00141	1.67	•	0.00158	1.87	•	0.00133	1.57	•
764.5231_4.83	PC(35:6)	0.02078	0.00991	0.48	•	0.01869	0.90		0.02341	1.13		0.03222	1.55	•	0.01950	0.94	
786.6002_5.30	PC(36:2)	1.78475	2.20218	1.23		3.56345	2.00	•	3.93336	2.20	•	3.75296	2.10	•	3.47608	1.95	•
766.57_4.99	PC(36:4)	0.00949	0.00935	0.99		0.01293	1.36		0.01458	1.54	•	0.01475	1.55	•	0.01143	1.20	
766.5719_4.78	PC(36:4)	0.00500	0.00453	0.91		0.00716	1.43		0.00870	1.74	•	0.00904	1.81	•	0.00754	1.51	•
782.56939_4.57	PC(36:4)	0.08602	0.10634	1.24		0.33036	3.84	•	0.40122	4.66	•	0.36702	4.27	•	0.21236	2.47	•
782.5694_4.82	PC(36:4)	1.33658	0.98068	0.73		1.69049	1.26		1.84909	1.38		1.99603	1.49		1.75526	1.31	
788.5521_4.99	PC(36:4)	0.00038	0.00036	0.94		0.00071	1.87		0.00079	2.06	•	0.00085	2.23	•	0.00069	1.80	•
780.5540_4.22	PC(36:5)	0.00861	0.00791	0.92		0.02328	2.70	•	0.02542	2.95	•	0.02349	2.73	•	0.01814	2.11	•
781.5896_5.93	PC(36:5)	0.00776	0.00750	0.97		0.00834	1.08		0.00993	1.28		0.01022	1.32		0.00864	1.11	
778.5362_4.54	PC(36:6)	0.00167	0.00161	0.96		0.00333	2.00	•	0.00393	2.36	•	0.00398	2.39	•	0.00285	1.71	•
798.60152_5.12	PC(37:3)	0.00565	0.00555	0.98		0.00824	1.46		0.00965	1.71	•	0.00887	1.57	•	0.00780	1.38	
813.6184_5.33	PC(37:4)	0.03054	0.02926	0.96		0.04426	1.45		0.05096	1.67	•	0.04932	1.62	•	0.04439	1.45	
794.5709_4.72	PC(37:5)	0.00752	0.00720	0.96		0.01030	1.37		0.01234	1.64	•	0.01223	1.63	•	0.01260	1.68	•
790.5744_4.89	PC(38:6)	0.01307	0.01505	1.15		0.02108	1.61	•	0.02421	1.85	•	0.02031	1.55	•	0.01912	1.46	
806.5698_4.70	PC(38:6)	13.77161	11.73420	0.85		11.55595	0.84		9.85537	0.72		10.70199	0.78		16.46454	1.20	
804.552_4.57	PC(38:7)	0.02174	0.02278	1.05		0.03830	1.76	•	0.04499	2.07	•	0.04193	1.93		0.03442	1.58	•
802.5358_4.49	PC(38:8)	0.00245	0.00245	1.00		0.00391	1.59	•	0.00462	1.88	•	0.00496	2.02	•	0.00487	1.98	•
824.6168_5.45	PC(39:4)	0.00653	0.00566	0.87		0.00745	1.14		0.00762	1.17		0.00794	1.22		0.00848	1.30	
830.5662_4.97	PC(40:8)	0.00770	0.00680	0.88		0.00787	1.02		0.00959	1.25		0.00888	1.15		0.01090	1.41	
830.5694_4.39	PC(40:8)	0.01671	0.01482	0.89		0.02422	1.45		0.02502	1.50	•	0.02363	1.41		0.02618	1.57	•
847.6036_4.55	PC(40:8)	0.00326	0.00364	1.12		0.00393	1.20		0.00407	1.25		0.00396	1.21		0.00395	1.21	
828.5589_4.1	PC(40:9)	0.00121	0.00155	1.28		0.00164	1.36		0.00204	1.69	•	0.00186	1.55	•	0.00197	1.64	•
873.7503_7.15	PC(42:1)	0.00508	0.00509	1.00		0.00541	1.06		0.00751	1.48		0.00772	1.52	•	0.00624	1.23	
852.6819_6.16	PC(42:4)	0.00305	0.00425	1.39		0.00384	1.26		0.00405	1.33		0.00425	1.39		0.00375	1.23	
822.5994_4.67	PC(42:5)	0.00542	0.00630	1.16		0.00895	1.65	•	0.00959	1.77	•	0.00962	1.77	•	0.00828	1.53	•
852.5592_4.46	PC(42:5)	0.00451	0.00530	1.18		0.00774	1.72	•	0.00867	1.92	•	0.00848	1.88	•	0.00832	1.85	•
846.6366_5.72	PC(42:6)	0.00096	0.00119	1.24		0.00103	1.08		0.00082	0.85		0.00101	1.05		0.00136	1.42	
858.6002_4.83	PC(42:8)	0.00135	0.00114	0.85		0.00192	1.42		0.00233	1.73	•	0.00203	1.50	•	0.00148	1.10	
880.5906_4.91	PC(42:8)	0.00523	0.00849	1.62	•	0.01301	2.49	•	0.01409	2.70	•	0.01214	2.32	•	0.01161	2.22	•
856.5836_4.47	PC(42:9)	0.00100	0.00109	1.10		0.00166	1.67	•	0.00204	2.04	•	0.00194	1.95	•	0.00182	1.82	•
854.5698_4.31	PC(42:10)	0.00314	0.00235	0.75		0.00391	1.24		0.00397	1.26		0.00392	1.25		0.00478	1.52	•
904.5912_4.88	PC(44:10)	0.04932	0.06070	1.23		0.05628	1.14		0.04945	1.00		0.04295	0.87		0.07776	1.58	•
902.5751_4.6	PC(44:11)	0.00087	0.00168	1.94		0.00207	2.39	•	0.00241	2.78	•	0.00211	2.44	•	0.00261	3.01	•
902.5758_4.47	PC(44:11)	0.00154	0.00092	0.60	•	0.00179	1.16		0.00199	1.29		0.00195	1.26		0.00151	0.98	
878.5758_4.53	PC(44:12)	0.00124	0.00159	1.29		0.00237	1.92	•	0.00268	2.17	•	0.00249	2.01	•	0.00245	1.98	•
935.7295_6.47	PC(46:6)	0.00600	0.00704	1.17		0.00641	1.07		0.00734	1.22		0.00749	1.25		0.00648	1.08	

Supplementary Table 10: ESI- lipids at Day 1 with fold changes of neutron contribution compared to photons.

m/z_Ret time	ID	C_D1		0_D1		5_D1		Fold change		15_D1		Fold change		25_D1		Fold change		83_D1		Fold change	
		Average	SD	Average	SD	Average	SD	to X-ray (0%)		Average	SD	to X-ray (0%)		Average	SD	to X-ray (0%)		Average	SD	to X-ray (0%)	
816.6101_5.48	LPE(20:2)	0.00761	0.00106	0.01581	0.00434	0.01203	0.00280	0.76		0.01048	0.00448	0.66	•	0.01526	0.00187	0.96		0.01154	0.00102	0.73	
848.5427_4.12	LPE(20:5)	0.02525	0.00374	0.03466	0.00831	0.01440	0.00794	0.42	•	0.02398	0.00298	0.69		0.02845	0.00399	0.82		0.02728	0.00700	0.79	
552.3088_0.97	LPE(24:0)	0.56085	0.05138	0.77887	0.09628	0.44786	0.14932	0.58	•	0.52323	0.10882	0.67		0.42828	0.07808	0.55	•	0.64692	0.13113	0.83	
836.5369_4.39	LPE(24:6)	0.00289	0.00093	0.00517	0.00114	0.00201	0.00143	0.39	•	0.00219	0.00087	0.42	•	0.00212	0.00046	0.41	•	0.00361	0.00128	0.70	
876.5744_4.69	PS(35:0)	0.11352	0.01123	0.15018	0.01446	0.09699	0.02167	0.65	•	0.11060	0.01165	0.74		0.09536	0.01680	0.63		0.12486	0.01844	0.83	
788.5792_5.06	PS(35:2)	0.01303	0.00294	0.02441	0.00677	0.01606	0.00447	0.66	•	0.01689	0.00322	0.69		0.02025	0.00208	0.83		0.01741	0.00228	0.71	
564.4057_1.04	PS(36:1)	0.04696	0.00950	0.26155	0.05807	0.09568	0.07313	0.37	•	0.14856	0.02855	0.57	•	0.12940	0.03309	0.49	•	0.15894	0.04788	0.61	•
840.6098_5.44	PS(38:0)	0.00798	0.00123	0.01482	0.00356	0.01099	0.00441	0.74		0.01367	0.00389	0.92		0.01603	0.00134	1.08		0.01294	0.00264	0.87	
772.5085_4.61	PS(38:1)	0.00013	0.00009	0.00143	0.00056	0.00139	0.00076	0.97		0.00199	0.00116	1.39		0.00089	0.00036	0.62	•	0.00140	0.00027	0.98	
850.5591_4.64	PS(38:4)	1.06631	0.10571	1.10082	0.10423	0.71036	0.17025	0.65	•	0.84502	0.12815	0.77		0.81499	0.12103	0.74		0.93054	0.12460	0.85	
826.5583_4.51	PS(39:1)	0.30203	0.08076	0.48978	0.08330	0.33569	0.14415	0.69		0.50082	0.05608	1.02		0.56804	0.03985	1.16		0.45085	0.04508	0.92	
504.3088_1.04	PS(40:2)	2.55461	0.37471	3.99549	0.37494	2.76957	1.13285	0.69		3.52140	0.28464	0.88		3.42185	0.45827	0.86		3.36180	0.50245	0.84	
812.5482_4.78	PS(40:5)	0.00131	0.00054	0.00474	0.00106	0.00280	0.00107	0.59	•	0.00299	0.00172	0.63	•	0.00373	0.00112	0.79		0.00387	0.00182	0.82	
832.5489_4.99	PS(41:1)	0.00188	0.00125	0.00288	0.00142	0.00370	0.00236	1.28		0.00507	0.00131	1.76	•	0.00675	0.00145	2.35	•	0.00306	0.00109	1.06	
498.2606_0.9	PS(41:4)	0.00201	0.00290	0.04351	0.01469	0.01745	0.00965	0.40	•	0.02342	0.00457	0.54	•	0.01647	0.00671	0.38		0.02254	0.01730	0.52	•
824.5428_4.17	PS(41:5)	0.02107	0.00657	0.04706	0.00927	0.02241	0.01023	0.48	•	0.03874	0.00635	0.82		0.04631	0.00526	0.98		0.04188	0.00932	0.89	
776.5433_4.69	PS(42:2)	0.01945	0.00333	0.03626	0.01078	0.01843	0.00640	0.51	•	0.02287	0.00264	0.63	•	0.02388	0.00233	0.66		0.03070	0.01005	0.85	
848.5431_4.36	PS(43:6)	0.00524	0.00077	0.00601	0.00113	0.00262	0.00156	0.44	•	0.00379	0.00142	0.63	•	0.00329	0.00053	0.55	•	0.00438	0.00141	0.73	
874.5584_4.32	PS(36:0)	0.02875	0.00241	0.04867	0.00285	0.02660	0.00938	0.55	•	0.03451	0.00576	0.71		0.03695	0.00547	0.76		0.04098	0.00840	0.84	
852.574_4.79	PS(36:0)	0.14948	0.01535	0.16121	0.01128	0.10263	0.01578	0.64	•	0.11137	0.01061	0.69		0.11872	0.01011	0.74		0.14346	0.02860	0.89	
834.5624_4.83	PS(36:0)	0.00446	0.00041	0.00748	0.00143	0.00576	0.00224	0.77		0.00493	0.00094	0.66	•	0.00417	0.00065	0.56	•	0.00594	0.00135	0.79	
826.5591_4.75	PS(38:3)	1.16325	0.16107	1.56064	0.10022	1.02967	0.24892	0.66	•	1.17412	0.14563	0.75		1.24918	0.12473	0.80		1.49187	0.30948	0.96	
812.5793_5.01	PS(38:3)	0.02062	0.00259	0.03824	0.00667	0.02805	0.00863	0.73		0.03131	0.00606	0.82		0.03447	0.00300	0.90		0.03252	0.00582	0.85	
838.5945_5.05	PS(38:3)	0.02137	0.00186	0.03757	0.00611	0.02816	0.00729	0.75		0.03569	0.00686	0.95		0.03796	0.00229	1.01		0.03211	0.00597	0.85	
906.6172_5.48	PS(38:3)	0.00430	0.00131	0.00423	0.00109	0.00122	0.00126	0.29	•	0.00225	0.00091	0.53	•	0.00172	0.00091	0.41	•	0.00213	0.00138	0.50	•

Supplementary Table 11: ESI- lipids at Day 7 with fold changes of neutron contribution compared to photons.

m/z_Ret time	ID	C_D7		0_D7		5_D7		Fold change		15_D7		Fold change		25_D7		Fold change		83_D7		Fold change	
		Average	SD	Average	SD	Average	SD	to X-ray (0%)		Average	SD	to X-ray (0%)		Average	SD	to X-ray (0%)		Average	SD	to X-ray (0%)	
816.6101_5.48	LPE(20:2)	0.00743	0.00261	0.01349	0.00461	0.01363	0.00371	1.01		0.01107	0.00281	0.82		0.01313	0.00285	0.97		0.01234	0.00158	0.92	
848.5427_4.12	LPE(20:5)	0.01418	0.00613	0.01848	0.00357	0.02797	0.00661	1.51	•	0.03063	0.00510	1.66	•	0.02789	0.00521	1.51	•	0.02853	0.00503	1.54	•
552.3088_0.97	LPE(24:0)	0.41243	0.09998	0.79446	0.07823	0.50429	0.07791	0.63	•	0.44256	0.04195	0.56	•	0.40278	0.08116	0.51	•	0.55492	0.08785	0.70	
836.5369_4.39	LPE(24:6)	0.00196	0.00082	0.00119	0.00080	0.00180	0.00054	1.51	•	0.00152	0.00029	1.27		0.00223	0.00114	1.87	•	0.00226	0.00070	1.90	•
876.5744_4.69	PS(35:0)	0.08776	0.01951	0.08870	0.01490	0.08550	0.01201	0.96		0.08299	0.01273	0.94		0.08263	0.01132	0.93		0.09116	0.00602	1.03	
788.5792_5.06	PS(35:2)	0.00897	0.00216	0.01370	0.00449	0.01591	0.00375	1.16		0.01503	0.00308	1.10		0.01467	0.00208	1.07		0.01377	0.00229	1.01	
564.4057_1.04	PS(36:1)	0.01448	0.01030	0.16219	0.09482	0.14339	0.04781	0.88		0.14485	0.02722	0.89		0.10410	0.02940	0.64	•	0.12070	0.04533	0.74	
840.6098_5.44	PS(38:0)	0.00607	0.00174	0.00732	0.00244	0.00844	0.00238	1.15		0.00863	0.00349	1.18		0.00892	0.00197	1.22		0.00781	0.00119	1.07	
772.5085_4.61	PS(38:1)	0.00016	0.00008	0.00033	0.00030	0.00011	0.00006	0.33	•	0.00014	0.00007	0.41	•	0.00022	0.00010	0.65	•	0.00020	0.00006	0.60	•
850.5591_4.64	PS(38:4)	1.00306	0.24831	0.95166	0.11115	0.82299	0.11081	0.86		0.73827	0.03612	0.78		0.79161	0.14669	0.83		1.02615	0.05883	1.08	
826.5583_4.51	PS(39:1)	0.19147	0.02712	0.26631	0.07216	0.51632	0.08146	1.94	•	0.56675	0.05673	2.13	•	0.53557	0.05852	2.01	•	0.35828	0.03156	1.35	
504.3088_1.04	PS(40:2)	1.69919	0.31977	3.59236	1.06610	3.52898	0.58461	0.98		3.48883	0.34220	0.97		3.07946	0.45176	0.86		3.10162	0.49713	0.86	
812.5482_4.78	PS(40:5)	0.00150	0.00113	0.00181	0.00162	0.00119	0.00080	0.66	•	0.00183	0.00099	1.01		0.00142	0.00080	0.79		0.00196	0.00135	1.08	
832.5489_4.99	PS(41:1)	0.00190	0.00141	0.00613	0.00316	0.00773	0.00258	1.26		0.00368	0.00092	0.60	•	0.00480	0.00259	0.78		0.00491	0.00218	0.80	
498.2606_0.9	PS(41:4)	0.00000	0.00000	0.00869	0.00426	0.00596	0.00595	0.69		0.02302	0.00443	2.65	•	0.01218	0.00431	1.40		0.01288	0.00509	1.48	
824.5428_4.17	PS(41:5)	0.01186	0.00347	0.01376	0.00350	0.04360	0.00807	3.17	•	0.04405	0.00556	3.20	•	0.04148	0.00822	3.01	•	0.02918	0.00620	2.12	•
776.5433_4.69	PS(42:2)	0.01571	0.00455	0.02208	0.00378	0.01790	0.00491	0.81		0.01945	0.00331	0.88		0.01964	0.00236	0.89		0.02642	0.01460	1.20	
848.5431_4.36	PS(43:6)	0.00380	0.00204	0.00460	0.00046	0.00318	0.00081	0.69		0.00295	0.00072	0.64	•	0.00334	0.00151	0.73		0.00552	0.00044	1.20	
874.5584_4.32	PS(36:0)	0.01981	0.00617	0.02026	0.00334	0.03379	0.00500	1.67	•	0.03393	0.00302	1.67	•	0.03187	0.00694	1.57	•	0.03443	0.00474	1.70	•
852.574_4.79	PS(36:0)	0.11951	0.02348	0.11517	0.01417	0.11952	0.01603	1.04		0.12228	0.01789	1.06		0.12381	0.02089	1.08		0.13251	0.00523	1.15	
834.5624_4.83	PS(36:0)	0.00375	0.00099	0.00566	0.00143	0.00499	0.00153	0.88		0.00512	0.00040	0.90		0.00479	0.00074	0.85		0.00558	0.00086	0.99	
826.5591_4.75	PS(38:3)	1.01850	0.24904	0.91217	0.07129	1.18042	0.17120	1.29		1.15661	0.07915	1.27		1.26673	0.20559	1.39		1.23185	0.11041	1.35	
812.5793_5.01	PS(38:3)	0.01592	0.00360	0.01472	0.00324	0.01938	0.00476	1.32		0.02062	0.00650	1.40		0.02041	0.00291	1.39		0.01818	0.00554	1.23	
838.5945_5.05	PS(38:3)	0.01599	0.00350	0.01523	0.00174	0.01602	0.00424	1.05		0.01707	0.00644	1.12		0.01508	0.00220	0.99		0.01517	0.00376	1.00	
906.6172_5.48	PS(38:3)	0.00242	0.00140	0.00207	0.00062	0.00392	0.00263	1.90	•	0.00235	0.00089	1.14		0.00170	0.00110	0.82		0.00203	0.00106	0.98	

Supplementary Table 12: ESI- lipids at Day 1 with IR fold changes compared to control

m/z_Ret time	ID	C_D1 Average	0_D1 Average	Fold change to Control		5_D1 Average	Fold change to Control		15_D1 Average	Fold change to Control		25_D1 Average	Fold change to Control		83_D1 Average	Fold change to Control	
816.6101_5.48	LPE(20:2)	0.00761	0.01581	2.08	●	0.01203	1.58	●	0.01048	1.38		0.01526	2.00	●	0.01154	1.52	●
848.5427_4.12	LPE(20:5)	0.02525	0.03466	1.37		0.01440	0.57	●	0.02398	0.95		0.02845	1.13		0.02728	1.08	
552.3088_0.97	LPE(24:0)	0.56085	0.77887	1.39		0.44786	0.80		0.52323	0.93		0.42828	0.76		0.64692	1.15	
836.5369_4.39	LPE(24:6)	0.00289	0.00517	1.79	●	0.00201	0.70		0.00219	0.76		0.00212	0.73		0.00361	1.25	
876.5744_4.69	PS(35:0)	0.11352	0.15018	1.32		0.09699	0.85		0.11060	0.97		0.09536	0.84		0.12486	1.10	
788.5792_5.06	PS(35:2)	0.01303	0.02441	1.87	●	0.01606	1.23		0.01689	1.30		0.02025	1.55		0.01741	1.34	
564.4057_1.04	PS(36:1)	0.04696	0.26155	5.57	●	0.09568	2.04	●	0.14856	3.16	●	0.12940	2.76		0.15894	3.38	●
840.6098_5.44	PS(38:0)	0.00798	0.01482	1.86	●	0.01099	1.38		0.01367	1.71		0.01603	2.01		0.01294	1.62	●
772.5085_4.61	PS(38:1)	0.00013	0.00143	10.63	●	0.00139	10.33	●	0.00199	14.80	●	0.00089	6.60	●	0.00140	10.42	●
850.5591_4.64	PS(38:4)	1.06631	1.10082	1.03		0.71036	0.67	●	0.84502	0.79		0.81499	0.76		0.93054	0.87	
826.5583_4.51	PS(39:1)	0.30203	0.48978	1.62	●	0.33569	1.11		0.50082	1.66	●	0.56804	1.88	●	0.45085	1.49	
504.3088_1.04	PS(40:2)	2.55461	3.99549	1.56	●	2.76957	1.08		3.52140	1.38		3.42185	1.34		3.36180	1.32	
812.5482_4.78	PS(40:5)	0.00131	0.00474	3.62	●	0.00280	2.14	●	0.00299	2.29	●	0.00373	2.86	●	0.00387	2.96	●
832.5489_4.99	PS(41:1)	0.00188	0.00288	1.53	●	0.00370	1.97	●	0.00507	2.70	●	0.00675	3.60	●	0.00306	1.63	
498.2606_0.9	PS(41:4)	0.00201	0.04351	21.64	●	0.01745	8.68	●	0.02342	11.65	●	0.01647	8.19	●	0.02254	11.21	
824.5428_4.17	PS(41:5)	0.02107	0.04706	2.23	●	0.02241	1.06		0.03874	1.84	●	0.04631	2.20	●	0.04188	1.99	
776.5433_4.69	PS(42:2)	0.01945	0.03626	1.86	●	0.01843	0.95		0.02287	1.18		0.02388	1.23		0.03070	1.58	
848.5431_4.36	PS(43:6)	0.00524	0.00601	1.15		0.00262	0.50	●	0.00379	0.72		0.00329	0.63	●	0.00438	0.84	
874.5584_4.32	PS(36:0)	0.02875	0.04867	1.69	●	0.02660	0.93		0.03451	1.20		0.03695	1.29		0.04098	1.43	
852.574_4.79	PS(36:0)	0.14948	0.16121	1.08		0.10263	0.69		0.11137	0.75		0.11872	0.79		0.14346	0.96	
834.5624_4.83	PS(36:0)	0.00446	0.00748	1.68	●	0.00576	1.29		0.00493	1.11		0.00417	0.94		0.00594	1.33	
826.5591_4.75	PS(38:3)	1.16325	1.56064	1.34		1.02967	0.89		1.17412	1.01		1.24918	1.07		1.49187	1.28	
812.5793_5.01	PS(38:3)	0.02062	0.03824	1.85	●	0.02805	1.36		0.03131	1.52	●	0.03447	1.67	●	0.03252	1.58	●
838.5945_5.05	PS(38:3)	0.02137	0.03757	1.76	●	0.02816	1.32		0.03569	1.67	●	0.03796	1.78	●	0.03211	1.50	●
906.6172_5.48	PS(38:3)	0.00430	0.00423	0.99		0.00122	0.28	●	0.00225	0.52	●	0.00172	0.40	●	0.00213	0.50	●

Supplementary Table 13: ESI- lipids at Day 7 with IR fold changes compared to control

m/z_Ret time	ID	C_D7 Average	0_D7 Average	Fold change to Control		5_D7 Average	Fold change to Control		15_D7 Average	Fold change to Control		25_D7 Average	Fold change to Control		83_D7 Average	Fold change to Control	
816.6101_5.48	LPE(20:2)	0.00743	0.01349	1.81	●	0.01363	1.83	●	0.01107	1.49		0.01313	1.77	●	0.01234	1.66	●
848.5427_4.12	LPE(20:5)	0.01418	0.01848	1.30		0.02797	1.97	●	0.03063	2.16	●	0.02789	1.97	●	0.02853	2.01	●
552.3088_0.97	LPE(24:0)	0.41243	0.79446	1.93	●	0.50429	1.22		0.44256	1.07		0.40278	0.98		0.55492	1.35	
836.5369_4.39	LPE(24:6)	0.00196	0.00119	0.61	●	0.00180	0.92		0.00152	0.77		0.00223	1.14		0.00226	1.16	
876.5744_4.69	PS(35:0)	0.08776	0.08870	1.01		0.08550	0.97		0.08299	0.95		0.08263	0.94		0.09116	1.04	
788.5792_5.06	PS(35:2)	0.00897	0.01370	1.53	●	0.01591	1.77	●	0.01503	1.67	●	0.01467	1.64	●	0.01377	1.53	
564.4057_1.04	PS(36:1)	0.01448	0.16219	11.20	●	0.14339	9.90	●	0.14485	10.00	●	0.10410	7.19	●	0.12070	8.33	●
840.6098_5.44	PS(38:0)	0.00607	0.00732	1.21		0.00844	1.39		0.00863	1.42		0.00892	1.47	●	0.00781	1.29	
772.5085_4.61	PS(38:1)	0.00016	0.00033	2.15	●	0.00011	0.72		0.00014	0.88		0.00022	1.41	●	0.00020	1.28	
850.5591_4.64	PS(38:4)	1.00306	0.95166	0.95		0.82299	0.82		0.73827	0.74		0.79161	0.79		1.02615	1.02	
826.5583_4.51	PS(39:1)	0.19147	0.26631	1.39		0.51632	2.70	●	0.56675	2.96	●	0.53557	2.80	●	0.35828	1.87	●
504.3088_1.04	PS(40:2)	1.69919	3.59236	2.11	●	3.52898	2.08	●	3.48883	2.05	●	3.07946	1.81	●	3.10162	1.83	●
812.5482_4.78	PS(40:5)	0.00150	0.00181	1.21		0.00119	0.80		0.00183	1.22		0.00142	0.95		0.00196	1.31	
832.5489_4.99	PS(41:1)	0.00190	0.00613	3.23	●	0.00773	4.08	●	0.00368	1.94	●	0.00480	2.53		0.00491	2.59	●
498.2606_0.9	PS(41:4)	0.00000	0.00869	-		0.00596	-		0.02302	-		0.01218	-		0.01288	-	
824.5428_4.17	PS(41:5)	0.01186	0.01376	1.16		0.04360	3.68	●	0.04405	3.71	●	0.04148	3.50	●	0.02918	2.46	●
776.5433_4.69	PS(42:2)	0.01571	0.02208	1.41		0.01790	1.14		0.01945	1.24		0.01964	1.25		0.02642	1.68	●
848.5431_4.36	PS(43:6)	0.00380	0.00460	1.21		0.00318	0.84		0.00295	0.78		0.00334	0.88		0.00552	1.45	
874.5584_4.32	PS(36:0)	0.01981	0.02026	1.02		0.03379	1.71	●	0.03393	1.71	●	0.03187	1.61	●	0.03443	1.74	●
852.574_4.79	PS(36:0)	0.11951	0.11517	0.96		0.11952	1.00		0.12228	1.02		0.12381	1.04		0.13251	1.11	
834.5624_4.83	PS(36:0)	0.00375	0.00566	1.51	●	0.00499	1.33		0.00512	1.36		0.00479	1.28		0.00558	1.49	
826.5591_4.75	PS(38:3)	1.01850	0.91217	0.90		1.18042	1.16		1.15661	1.14		1.26673	1.24		1.23185	1.21	
812.5793_5.01	PS(38:3)	0.01592	0.01472	0.92		0.01938	1.22		0.02062	1.30		0.02041	1.28		0.01818	1.14	
838.5945_5.05	PS(38:3)	0.01599	0.01523	0.95		0.01602	1.00		0.01707	1.07		0.01508	0.94		0.01517	0.95	
906.6172_5.48	PS(38:3)	0.00242	0.00207	0.86		0.00392	1.62	●	0.00235	0.97		0.00170	0.70		0.00203	0.84	
